# Supplementary material for: Health and sustainability of glaciers in High Mountain Asia
Source: Nat Commun. 2021 May 17;12:2868. doi: 10.1038/s41467-021-23073-4 (PMC8129093; doi:10.1038/s41467-021-23073-4)
Supplement: Supplementary file 1 — Supplementary Information [file 41467_2021_23073_MOESM1_ESM.pdf]

# Supplementary Information for Health and sustainability of glaciers in High Mountain Asia

Evan Miles (evan.miles@wsl.ch)[1], Michael McCarthy[1,2], Amaury Dehecq[1,3], Marin Kneib[1,4], Stefan Fugger[1,4], Francesca Pellicciotti[1,5]

[1]Swiss Federal Research Institute WSL, 8906 Birmensdorf, Switzerland
[2]British Antarctic Survey, Natural Environment Research Council, Madingley Road, Cambridge, UK
[3]Laboratory of Hydraulics, Hydrology and Glaciology, ETH Zurich, 8093 Zurich, Switzerland
[4]Institute of Environmental Engineering, ETH Zurich, 8093 Zurich, Switzerland
[5]Department of Geography, Northumbria University, Newcastle, NE1 7RU, UK

# Contents

Supplementary Notes ..... 2
Supplementary Note 1: Comparison to SMB measurements ..... 2
Supplementary Note 2: Comparison to results of Bisset et al (2020) ..... 3
Supplementary Note 3: Comparison to ELA estimates ..... 3
Supplementary Note 4: Sensitivity to ice thickness model ..... 3
Supplementary Tables ..... 5
Supplementary Figures ..... 11
Supplementary References ..... 91

# SUPPLEMENTARY NOTES

## SUPPLEMENTARY NOTE 1: COMPARISON TO SMB MEASUREMENTS

Relatively few in-situ measurements of glacier mass balance have been reported for High Mountain Asia [1–3]. We first compiled a database of all known and available glaciological mass balance measurements for High Asian glaciers during the 2000-2016 study period (Supplementary Table 1). Measurements were sourced from the World Glacier Monitoring Service [4] or compiled from published studies. Where possible, data were sourced directly from open archives, while observations for many glaciers were collected by manually digitizing data from figures within peer-reviewed literature. We were unable to source records for some glaciers, which are described but not depicted in scientific literature. After limiting the dataset to the study period and where SMB observations coincided with our needed inputs, we were left with 35 glaciers varying in size, location, and characteristics (Supplementary Table 1, Supplementary Figure 24).

A direct comparison between our results and these measurements is problematic due to differences in observation period and spatial scale, as well as limitations with the underlying datasets. In terms of spatial scale, our results are fully distributed across the study glaciers, whereas point mass balance measurements tend to have a bias towards accessible locations that may or may not be representative of their entire elevation band. In addition, there was a strong bias to lower elevations of glaciers: very few sites contained any measurements of accumulation at all. As there can be considerable local heterogeneity in SMB, we calculate the range of SMB values within each elevation band. With regards to temporal scale, our results are effective for the 2000-2016 period, but few sites include measurements encompassing at least half of this period. The few sites with at least 10 years of observation also show considerable interannual variability in SMB.

Given the difficulties of this comparison, we apply the SMB reconstruction method to these glaciers to first assess the method's performance qualitatively. We focus on three aspects describing the pattern and accuracy of derived SMB: coherence of the SMB pattern, SMB altitudinal gradient, and apparent bias in SMB magnitude. In addition, we compare the altitudinal and spatial pattern of derived SMB to each glacier's modelled thickness and measured surface velocity to consider most likely causes of poorer results (Supplementary Figures 25-60).

The results from our method are often satisfactory, but differ substantially from measurements in many cases. In particular, although our method generally reproduces the patterns of observed mass balances for glaciers which exhibit a clear surface velocity pattern, it often struggles for glaciers which exhibit low or undulating surface velocities. These problems have been previously acknowledged due to the chip size and offset settings of the ITS_LIVE JPL auto-RIFT processing chain and its use of moderate resolution Landsat imagery [5,6]. Some glaciers were also represented poorly in the Randolph Glacier Inventory 6.0 (RGI6.0) [7,8] on which the ice thickness estimates are based [9], leading to incomparable results.

Despite the disagreement in spatial and temporal scales between the field measurements of surface mass balance and our results, we also perform a quantitative comparison by asking whether our results are consistent with the field measurements. For nearly all sites, the only position information for the field measurements is an elevation. Considering the possibility of a bias between field elevation values and the ASTER GDEM v2 which we use, we therefore sampled our results based on a window of +/- 50 m around the elevation for each reported surface mass balance measurement. We then determined the minimum bias between our results for this window and the reported surface mass balance measurement. From our database of 2088 reported values of surface mass balance, we find a mean bias of 0.16 m w.e. $a^{-1}$, and a median absolute deviation of 0.025 m w.e. $a^{-1}$ (Supplementary Table 1). In all, we find that 79% of reported surface mass balance measurements correspond to within 0.2 m w.e. $a^{-1}$ of our results. Acknowledging that (1) many glacier outlines contain tributaries with distinct mass balance regimes for the same elevation, (2) glacier surface mass balance patterns exhibit strong interannual variability, we consider this result to be a very positive indicator in terms of the method's performance. We additionally compare the equilibrium line altitudes computed based on our results to those estimated based on the field measurements for each glacier (Supplementary Table 1), and find close correspondence, with an R2 of 0.91 and a slope of 1.017 (Supplementary Figure 86).

Based on these comparisons, we limit our analyses to larger glaciers (>2km$^2$ in area) which are more likely to show a clear velocity signal. We also remove surging glaciers from consideration for further processing, which we identify based on the RGI6.0 attributes and with data from [6]. We additionally identify glaciers with erratic surface lowering or mass balance patterns, also indicative of surging or lower quality source data. In particular,

we limit our glaciers for further analysis to those that satisfy the following conditions: the altitudinal *dH/dt* profile has a standard deviation of less than 3 m $a^{-1}$ and the *dH/dt* profile has a nonnegative correlation with elevation. We consider these characteristics to be indicative of surging behaviour. Finally, we only retain glaciers with the following criteria, which we consider to be indicative of higher quality input data and results: the optimized ELA has an Accuracy of at least 0.5, the detrended SMB profile has a standard deviation of less than 3 m w.e. $a^{-1}$; and the mean SMB uncertainty is less than 3 m w.e. $a^{-1}$.

## SUPPLEMENTARY NOTE 2: COMPARISON TO RESULTS OF BISSET ET AL (2020)

We additionally compare our results to those of [10] (hereafter, B2020), who examined a population of 25 glaciers across High Mountain Asia using a continuity approach similar to our own but with distinct *dH/dt* data and manual processing of glacier segments. Their analysis was restricted to larger glaciers generally comparable to those included in our analysis, and thus provides a good assessment of whether our results are 1) reasonable in terms of spatial pattern and 2) consistent with prior estimates. Crucially, that study did not use field measurements for validation, but compared the altitudinal mass balance gradients in the clean ice ablation area to those reported in past studies.

The results of this comparison are presented in Supplementary Figures 61-85. Our results are very consistent with those of B2020. Differences are apparent in the segmentation approach, which often leads to a clearer mass balance gradient in the B2020 results as glaciers may be segmented to avoid erroneous input data. However, this is mostly due to small glacier segments in our analysis, which can occasionally reveal important mass balance differences along tributaries but give the appearance of an erratic mass balance profile. It is important to note that B2020 focused only on the main glacier trunks, and in these areas our estimates agree closely. Finally, we note that our uncertainty estimates are generally also in agreement with those of B2020.

## SUPPLEMENTARY NOTE 3: COMPARISON TO ELA ESTIMATES

Second, we assembled independent ELA estimates for glaciers within the region. Numerous previous studies have reported ELAs for individual glaciers based on field measurements, but these estimates correspond almost exclusively to the glaciers listed above for the SMB validation. Instead, we focus on independent estimates, which have primarily used the on-glacier snowline altitude at the end of the ablation season to indicate the annual ELA. While these metrics are not precisely the same, they tend to correspond closely [11]. Results from these studies generally are compiled for several years within our study period, so we expect the mean value to be reasonably comparable to our 16-year effective ELA. We therefore compare our ELA estimates to those of [12], who provide a value for each of 287 glaciers across the main Himalayan Arc, on a glacier-specific basis (Supplementary Figure 87). We also compare the results of selected studies of seasonal snowline elevations [13–17] to our ELA estimates for the corresponding area (Supplementary Figure 88). We additionally compare our results to the ELAs estimated by [18] based on debris-covered area extent (Supplementary Figure 89). These past studies collectively represent a small fraction of the glacier area in our study, but are well distributed across the entire region. These three comparisons demonstrate a close correspondence of ELA values between our results and published estimates.

## SUPPLEMENTARY NOTE 4: SENSITIVITY TO ICE THICKNESS MODEL

While we are confident in the applicability of the continuity approach to reconstruct SMB estimates based on past applications [19–27], there is some circularity to the approach based on the modelled ice thickness distributions of [9]. Some of the contributing thickness models are based on the flux reconstruction method, and assume a climatic-basal mass balance gradient to determine ice fluxes in the first place. This is the case for [28], and the method of [29] implements a similar flux-reconstruction approach, but updates the thickness field based on velocity observations (this is probably the most relevant for our approach). While this circularity could be problematic in terms of the estimated pattern and value of SMB, the consensus results [9] are presently the most robust estimates of ice thickness for mountain glaciers globally. Past implementations of the continuity approach have ignored this circularity problem entirely and derived indicative mass balance patterns [26], even in the case of debris-covered glaciers [10].

It is important to note that the consensus ice thickness [9] that we use is not based exclusively on this flux-reconstruction approach - the method of [30] uses an entirely independent approach based on basal shear stress estimation, which may partially mitigate against the circularity. In addition, we have validated the SMB patterns and derived ELAs using all available data, and most results are satisfactory. For our purposes, we consider the

consensus ice thickness results to be the best ice thickness dataset currently available, and have represented its uncertainty in our results. There is certainly room for improvement of ice thickness models to better account for relevant processes, including distinct tributaries and the effects of debris cover. Future efforts should revisit the SMB patterns and integrate the continuity equation and velocity observations systematically into a globally-applicable model, but this is out of our current scope.

Nonetheless, to assess the severity of this circularity problem for our results, we test the sensitivity of our method to the five different bed models. We thus apply our approach to each ice thickness dataset contributing to the consensus estimate. We pay particular attention to differences between Models 1 and 2, which correspond to fundamentally different approaches to model glacier thickness. Below are depictions of regional (Supplementary Figure 90) and subregional (Supplementary Figures 91-92) glacier mass balance profiles from each method, along with deviations in the derived ELA, AAR, and balance ratio values (Supplementary Figures 93-95). It is important to note that Models 3-5 have only partial coverage within our domain, so mass balance profiles are not comparable in all cases. Accounting for the variable coverage of the distinct models, the results are quite consistent at the regional and subregional scale in terms of mass balance profiles. In addition, it is clear that significant deviations in ELA and AAR only occur for a minority of glaciers. Moreover, the deviations from the consensus thickness results for models 1 and 2 do not have opposite signals, which indicates no systematic difference. Based on these results, we are quite confident that our use of the consensus ice thickness is not compromised by the potential circularity of contributing thickness models.

## SUPPLEMENTARY TABLES

Supplementary Table 1. Glaciers in High Mountain Asia for which we could access measurements of surface mass balance, and qualitative summary of our results.

| N | RGIID | Name | Country | Lat. | Long. | Area (km²) | *N* yrs | Sources | Obs. Years | Qualitative performance | Abl. Grad m w.e. (m a.s.l.)$^{-1}$ | Field ELA m a.s.l. | Our ELA m a.s.l. | Median abs. deviation SMB (m w.e. a$^{-1}$) | % deviation < 0.2 m w.e. a$^{-1}$ |
|---|---|---|---|---|---|---|---|---|---|---|---|---|---|---|---|
| 1 | 13.06361 | Kara-Batkak | Kyrgyz. | 42.140 | 78.273 | 2.046 | 4 | WGMS | 2014-2017 | Poor; no observed velocity | 0.0041 | 4082 | 3926 | 0.016 | 78% |
| 2 | 13.06974 | Batysh Sook | Kyrgyz. | 41.787 | 77.749 | 0.954 | 4 | WGMS, Kenzhebaev et al. (2017) | 2011-2014 | Good fit, no observed velocity | 0.0058 | 4273 | 4209 | 0.005 | 98% |
| 3 | 13.07064 | Glacier N354 | Kyrgyz. | 41.793 | 78.164 | 6.537 | 6 | WGMS, Hoelzle et al., (2017) | 2011-2016 | Good fit, little observed velocity | 0.0068 | 4222 | 4360 | 0.002 | 92% |
| 4 | 13.08054 | Bordu (North) | Kyrgyz. | 41.813 | 78.175 | 5.557 | 2 | WGMS | 2016-2017 | Good fit, no observed velocity | 0.0039 | 4459 | 4231 | 0.005 | 100% |
| 5 | 13.08055 | Sary Tor | Kyrgyz. | 41.827 | 78.181 | 2.925 | 3 | WGMS, Hoelzle et al., (2017) | 2015-2017 | Good fit, no observed velocity | 0.0044 | 4511 | 4716 | 0.005 | 100% |
| 6 | 13.08624 | Ts. Tuyuksuyskiy | Kyrgyz. | 43.044 | 77.081 | 2.838 | 17 | WGMS | 2000-2016 | Poor; little observed velocity | 0.0064 | 3830 | 3765 | 0.008 | 98% |
| 7 | 13.10093 | Glacier N599 | Kyrgyz. | 42.793 | 76.867 | 2.234 | 2 | WGMS | 2015-2016 | No *dH/dt* data | --- | --- | --- | --- | --- |
| 8 | 13.11609 | Golubin | Kyrgyz. | 42.454 | 74.498 | 4.827 | 6 | WGMS, Hoelzle et al., (2017) | 2011-2016 | Poor, likely velocity underestimation | 0.0091 | 3777 | 3835 | 0.014 | 79% |
| 9 | 13.18096 | Abramov | Kyrgyz. | 39.620 | 71.560 | 21.345 | 5 | WGMS, Hoelzle et al., (2017) | 2012-2016 | Good fit | 0.0084 | 4191 | 4146 | 0.017 | 87% |
| 10 | 13.24602 | Xiaodongkemadi | China | 33.082 | 92.063 | 15.97 | 10 | Yao et al., (2012)***; Liang, Cuo, & Liu, (2018); R. Wang, Liu, Shangguan, Radić, & Zhang, (2019) | unknown | No *dH* data | --- | --- | --- | --- | --- |
| 11 | 13.28839 | Gurenhekou | China | 30.193 | 90.447 | 1.333 | 6 | Yao et al., (2012)***; Yu et al., 2013) | 2004-2010 | Good, but no velocity, no accumulation | 0.0088 | 5788 | 5978 | 0.023 | 80% |
| 12 | 13.32330 | Qiyi | China | 39.237 | 97.755 | 2.531 | 10 | Liang et al., (2018); Wang et al., (2019); Wu, He, Jiang, & Wang, (2016) | unknown | No velocity data | --- | --- | --- | --- | --- |
| 13 | 13.41891 | Muztag Ata | China | 38.230 | 75.050 | 1.087 † | 5 | Wang et al., 2019; Yao et al., (2012)***; Zhu, Yao, Yang, Xu, Wu, Wang, et al., (2018) | 2002, 2006-2010 | Good fit, little observed velocity | N/A | 5480 | 5532 | 0.025 | 81% |
| 14 | 13.45336 | Urumqi N1 | China | 43.118 | 86.811 | 0.458 †† | 15 | WGMS | 2000, 2001, 2004-2016 | No velocity data | --- | --- | --- | --- | --- |
| 15 | 13.49645 | Zhongxi | China | 30.868 | 91.443 | 2.579 | 3 | Yao et al., (2012)*** | 2007-2010 | No *dH/dt* data | --- | --- | --- | --- | --- |

†Split into multiple polygons in RGI. ††Multiple glaciers share one polygon in RGI. *Continuity approach. **Idealised SMB profile. ***SMB contours. N/A indicates that a value could not be determined from the data. 'Deb' indicates that ablation measurements are only available for the debris-covered area, so no ablation gradient is reported.

Supplementary Table 1 (continued). Glaciers in High Mountain Asia for which we could access measurements of surface mass balance, and summary of our results.

| N | RGIID | Name | Country | Lat. | Long. | Area (km²) | *N* yrs | Sources | Obs. Years | Qualitative performance | Abl. Grad m w.e. (m a.s.l.)$^{-1}$ | Field ELA m a.s.l. | Our ELA m a.s.l. | Median abs. deviation SMB (m w.e. a$^{-1}$) | % deviation < 0.2 m w.e. a$^{-1}$ |
|---|---|---|---|---|---|---|---|---|---|---|---|---|---|---|---|
| 16 | 13.49754 | Zhadang | China | 30.472 | 90.639 | 1.506 | 2 | Yu et al., (2013); Zhu, Yao, Yang, Xu, Wu, & Wang, (2018) | 2005-2007 | OK, but no velocity, no accumulation | 0.0081 | 5797 | 5965 | 0.017 | 85% |
| 17 | 14.11566 | Naradu | India | 31.286 | 78.431 | 3.017 | 7 | Koul & Ganjoo, (2010) | 2000-2002 | OK fit, little observed velocity | 0.003 | 4950 | 5282 | 0.047 | 82% |
| 18 | 14.14383 | Stok | India | 33.977 | 77.45 | 0.782 | 5 | Soheb et al., (2020) | 2014-2019 | No velocity data | | | --- | --- | --- |
| 19 | 14.15536 | Hamtah | India | 32.227 | 77.517 | 3.434 | 1 | Laha et al., (2017)** | 2008 | Very good fit | 0.0032 | 4784 | 4522 | 0.030 | 88% |
| 20 | 14.15990 | Chhota Shigri | India | 32.280 | 77.520 | 16.764 †† | 12 | Azam et al., (2016); Wagnon et al., (2007) | 2002-2013 | Good, some velocity gaps | 0.0054 | 5107 | 4767 | 0.076 | 71% |
| 21 | 14.16041 | Sutri Dhaka | India | 33.400 | 77.500 | 20.384 | 2 | Pratap et al., (2019); Sharma et al., (2016) | 2015-2016 | Poor; velocity gaps and underestimation | 0.0063 | 5505 | 4959 | 0.086 | 59% |
| 22 | 15.02291 | Ganju La | Bhutan | 27.940 | 89.950 | 0.329 † | 3 | Tshering & Fujita, (2016) | 2003, 2012-2013 | No velocity data | --- | --- | --- | --- | --- |
| 23 | 15.03416 | Pokalde | Nepal | 27.927 | 86.831 | 0.107 | 6 | Sherpa et al., (2017); Wagnon et al., (2013) | 2010, 2012-2014 | Good fit, no observed velocity | 0.0157 | 5606 | 5730 | 0.051 | 85% |
| 24 | 15.03448 | Trambau | Nepal | 27.880 | 86.530 | 28.574 †† | 3 | Sunako, Fujita, Sakai, & Kayastha, (2019) | 2016-2019 | Very good, slight offset | 0.0063 | 5816 | 5838 | 0.035 | 82% |
| 25 | 15.03507 | AX010 | Nepal | 27.716 | 86.556 | 0.421 | 4 | Fujita, Kadota, Rana, Kayastha, & Ageta, (2001); Fujita & Nuimura, (2011); Wang et al. (2019) | 1995-1999 | Poor; no observed velocity | 0.0143 | 5207 | N/A | 0.063 | 70% |
| 26 | 15.03586 | Mera | Nepal | 27.700 | 86.900 | 1.436 †† | 8 | WGMS, Sherpa et al., (2017); Wagnon et al., (2013) | 2007-2010, 2012-2013 | Good fit, little observed velocity | 0.0059 | 5446 | 5854 | 0.040 | 72% |
| 27 | 15.03733 | Khumbu | Nepal | 27.975 | 86.903 | 19.097 | * | Nuimura et al., (2011) | 1995-2004 | Very good, but velocity gaps | Deb | N/A | 5318 | 0.050 | 79% |
| 28 | 15.03734 | Changri Nup | Nepal | 27.980 | 86.770 | 12.451 †† | 5 | Sherpa et al., (2017); Vincent et al., (2016) | 2010-2015 | Very good fit | 0.0096 | 5608 | 5557 | 0.007 | 92% |
| 29 | 15.03954 | Yala | Nepal | 28.236 | 85.618 | 2.107 | 4 | WGMS, Acharya & Kayastha, (2018); Baral et al., (2014) | 2012-2015 | Underestimation; no observed velocity | 0.0118 | 5434 | 5552 | 0.007 | 90% |
| 30 | 15.04045 | Lirung | Nepal | 28.239 | 85.556 | 1.46 † | 1, * | Own data | 2000-2010; 2013 | Good pattern, slight underestimation | Deb | N/A | 4550 | 0.025 | 92% |
| 31 | 15.04847 | Rikha Samba | Nepal | 28.827 | 83.490 | 6.504 †† | 2 | Gurung et al., (2016) | 2011-2012 | Good; likely velocity underestimation | 0.007 | 5870 | N/A | 0.047 | 82% |

†Split into multiple polygons in RGI. ††Multiple glaciers share one polygon in RGI. *Continuity approach. **Idealised SMB profile. ***SMB contours. N/A indicates that a value could not be determined from the data. 'Deb' indicates that ablation measurements are only available for the debris-covered area, so no ablation gradient is reported.

Supplementary Table 1 (continued). Glaciers in High Mountain Asia for which we could access measurements of surface mass balance, and summary of our results.

| N | RGIID | Name | Country | Lat. | Long. | Area ($km^2$) | *N* yrs | Sources | Obs. Years | Qualitative performance | Abl. Grad m w.e. (m a.s.l.)$^{-1}$ | Field ELA m a.s.l. | Our ELA m a.s.l. | Mean bias field vs our SMB (m w.e. $a^{-1}$) | % deviation < 0.5 m w.e. $a^{-1}$ |
|---|---|---|---|---|---|---|---|---|---|---|---|---|---|---|---|
| 32 | 15.06777 | Dunagiri | India | 30.558 | 79.894 | 2.4 | 1** | Laha et al., (2017)** | 1986** | Good pattern; suspect MB profile | 0.0027 | 4913 | 4656 | 0.180 | 51% |
| 33 | 15.07122 | Satopanth | India | 30.790 | 79.414 | 49.314 †† | 2 | Singh, Banerjee, Nainwal, & Shankar, (2019) | 20142016 | Very good fit | Deb | N/A | 4641 | 0.015 | 100% |
| 34 | 15.07143 | Chorabari | India | 30.793 | 79.041 | 3.199 | 7 | WGMS, Dobhal, Mehta, & Srivastava, (2013) | 2011-2012 | Poor; little observed velocity | 0.0029 | 5094 | 4617 | 0.422 | 47% |
| 35 | 15.07605 | Dokriani | India | 30.843 | 78.825 | 6.518 | 4 | Pratap, Dobhal, Mehta, & Bhambri, (2015) | 2009-2012 | Poor; little observed velocity | 0.0036 | 5122 | 4933 | 0.629 | 34% |
| 36 | 15.07645 | Baishui Glacier N1 | China | 27.104 | 100.187 | 1.281 | 4 | Yu, He, Li, Wang, & Niu, (2015) | 2008, 2011-2012 | No velocity data | --- | --- | --- | | |
| 37 | 15.09026 | Naimona'nyi | China | 30.455 | 81.323 | 7.348 | 5 | Yao et al., (2012)***; Zhao et al., (2016) | 2005-2010 | OK fit, little observed velocity | 0.0032 | 6186 | 6039 | 0.009 | 81% |
| 38 | 15.10263 | Kangwure | China | 28.470 | 85.816 | 1.872 † | 3 | Yao et al., (2012)*** | 2005, 2009-2010 | OK fit, little observed velocity | 0.0085 | 5979 | N/A | 0.013 | 83% |
| 39 | 15.11758 | 24K | China | 29.752 | 95.729 | 6.083 | 1 | Wei, Tandong, Baiqing, & Hang, (2010) | 2007 | Very good, slight offset | 0.019 | 4318 | 4192 | 0.056 | 79% |
| 40 | 15.11962 | Parlung 12 | China | 29.303 | 96.902 | 0.23 | 5 | Yao et al., (2012)*** | 2005-2010 | OK, no observed velocity | 0.007 | 5438 | 5358 | 0.020 | 76% |
| 41 | 15.11963 | Parlung 10 | China | 29.286 | 96.904 | 4.433 | 4 | Yao et al., (2012)*** | 2005-2009 | Good pattern, slight underestimation | 0.0086 | 5408 | 5379 | 0.016 | 81% |
| 42 | 15.11973 | Parlung N94 | China | 29.386 | 96.976 | 11.858 | 11 | WGMS | 2006-2016 | Highly variable; erratic velocity pattern | 0.011 | 5287 | 5149 | 0.251 | 40% |
| 43 | 15.12707 | Parlung 390 | China | 29.357 | 97.019 | 0.372 | 4 | Yao et al., (2012)*** | 2006-2010 | OK fit, little observed velocity | 0.0078 | 5443 | N/A | 0.030 | 97% |

†Split into multiple polygons in RGI. ††Multiple glaciers share one polygon in RGI. *Continuity approach. **Idealised SMB profile. ***SMB contours. N/A indicates that a value could not be determined from the data. 'Deb' indicates that ablation measurements are only available for the debris-covered area, so no ablation gradient is reported.

Supplementary Table 2. Assessment of bias of glacier subset in terms of geodetic mass balance and interpretation in terms of impact on AAR based on relationship between geodetic mass balance and AAR (Supplementary Figure 12). We note that the subset geodetic biases are less that the mass balance uncertainty for all regions, and infer AAR uncertainty of a similar magnitude. We also calculate the regional average specific mass balance based on our glacier subset, and compare it to that derived by the geodetic data alone for the same glaciers [63].

| | **Biases of subset to whole region** | | **Mean geodetic mass balance from dH/dt** | | | **Subregional mean SMB** | |
|---|---|---|---|---|---|---|---|
| ***Region*** | Geodetic MB bias (m w.e./a) | Apparent AAR bias (-) | Subset (m w.e./a) | All >2km$^2$ (m w.e./a) | All (m w.e./a) | Subset mean SMB (m w.e. /a) | Difference to subset geodetic MB (m w.e. /a) |
| *Inner TP* | 0.04 | 0.03 | -0.10 ± 0.03 | -0.08 | -0.14±0.07 | -0.21 ± 0.04 | -0.11 ± 0.05 |
| *Kunlun* | 0.02 | 0.02 | 0.16 ± 0.04 | 0.14 | 0.14±0.08 | 0.02 ± 0.06 | -0.14 ± 0.07 |
| *Pamir Alai* | -0.02 | -0.02 | -0.06 ± 0.07 | -0.07 | -0.04±0.07 | -0.17 ± 0.12 | -0.11 ± 0.14 |
| *Nyainqentangla* | 0.13 | 0.11 | -0.49 ± 0.08 | -0.52 | -0.62±0.23 | -0.68 ± 0.13 | -0.19 ± 0.15 |
| *Everest* | 0.00 | 0.00 | -0.33 ± 004 | -0.30 | -0.33±0.20 | -0.43 ± 0.09 | -0.10 ± 0.10 |
| *Bhutan* | 0.05 | 0.04 | -0.37 ± 0.05 | -0.37 | -0.42±0.20 | -0.46 ± 0.18 | -0.09 ± 0.19 |
| *West Nepal* | 0.09 | 0.07 | -0.23 ± 0.22 | -0.33 | -0.34±0.09 | -0.46 ± 0.23 | -0.23 ± 0.32 |
| *Karakoram* | 0.02 | 0.01 | -0.01 ± 0.04 | -0.04 | -0.03±0.07 | -0.13 ± 0.08 | -0.12 ± 0.09 |
| *Spiti Lahaul* | -0.05 | 0.04 | -0.42 ± 0.12 | -0.39 | -0.37±0.09 | -0.51 ± 0.15 | -0.09 ± 0.19 |
| *Pamir* | -0.02 | -0.02 | -0.10 ± 0.03 | -0.10 | -0.08±0.07 | -0.17 ± 0.07 | -0.07 ± 0.08 |
| *Hindu Kush* | -0.01 | 0.01 | -0.13 ± 0.08 | -0.11 | -0.12±0.07 | -0.21 ± 0.27 | -0.08 ± 0.28 |
| *Tien Shan* | 0.07 | 0.05 | -0.21 ± 0.04 | -0.20 | -0.28±0.20 | -0.34 ± 0.08 | -0.13 ± 0.10 |
| ***All*** | **0.02** | **0.03** | **-0.16 ± 0.02** | **-0.14** | **-0.18±0.04** | **-0.28 ± 0.03** | **-0.12 ± 0.04** |

Supplementary Table 3. Summary of the results for each subregion, including processed glacier area (Area) and percent of regional area of glaciers over 2 km$^2$, processed number ($N$) and percent of regional glaciers over 2 km$^2$, area-weighted mean and standard deviation of ELAs and AARs, percentage of analysed glaciers with low or high AARs, modelled volume change by 2100 (Implied $\Delta V$) and uncertainty, and the ablation balance ratio (Balance Ratio) and uncertainty. Note that these values correspond to the subset of glaciers which we have processed for the region.

| | **Area** | | **N** | | **ELA** | **ELA std** | **AAR** | **AAR std** | **AAR <0.2** | **AAR >0.7** | **Implied $\Delta V$** | **Implied $\Delta V$ unc** | **TotAbl** | **TotAbl unc** |
|---|---|---|---|---|---|---|---|---|---|---|---|---|---|---|
| ***Region*** | km$^2$ | % | - | % | m a.s.l. | m a.s.l. | - | - | % | % | % | % | Gt a$^{-1}$ | Gt a$^{-1}$ |
| *Inner TP* | 5189 | 64% | 764 | 58% | 5700 | 360 | 0.40 | 0.31 | 36% | 21% | -16% | 2% | 43% | 8% |
| *Kunlun* | 5116 | 64% | 585 | 67% | 5464 | 428 | 0.77 | 0.26 | 8% | 74% | 2.1% | 0.2% | 114% | 24% |
| *Pamir Alai* | 446 | 53% | 96 | 63% | 4115 | 399 | 0.54 | 0.33 | 32% | 27% | -17% | 2% | 58% | 9% |
| *Nyainqentangla* | 2608 | 59% | 417 | 67% | 5350 | 418 | 0.26 | 0.25 | 50% | 7% | -57% | 15% | 38% | 6% |
| *Bhutan* | 1054 | 69% | 163 | 70% | 5839 | 447 | 0.41 | 0.26 | 36% | 7% | -36% | 4% | 41% | 4% |
| *West Nepal* | 2091 | 69% | 343 | 79% | 5581 | 438 | 0.40 | 0.26 | 37% | 12% | -38% | 6% | 39% | 6% |
| *Karakoram* | 5862 | 34% | 877 | 70% | 5395 | 546 | 0.57 | 0.34 | 31% | 37% | -11% | 1% | 71% | 12% |
| *Spiti Lahaul* | 3381 | 76% | 543 | 76% | 5200 | 483 | 0.38 | 0.26 | 41% | 8% | -40% | 5% | 38% | 5% |
| *Pamir* | 1022 | 22% | 251 | 45% | 4930 | 383 | 0.47 | 0.28 | 27% | 19% | -17% | 1% | 56% | 8% |
| *Hindu Kush* | 1999 | 66% | 316 | 68% | 4837 | 408 | 0.55 | 0.29 | 23% | 28% | -16% | 1% | 66% | 6% |
| *Tien Shan* | 5028 | 68% | 768 | 79% | 4262 | 395 | 0.43 | 0.30 | 42% | 17% | -31% | 5% | 44% | 8% |
| ***All*** | **36656** | **56%** | **5527** | **68%** | **5283** | **678** | **0.51** | **0.32** | **41%** | **31%** | **-23%** | **1%** | **50%** | **3%** |

Supplementary Table 4. Summary of outcomes for each of the principal drainage basins in High Mountain Asia, following the AOP basins. Note that most results report values from our subset of glaciers, as in Supplementary Table 3, while Balance and Total Ablation values are extended to represent the entire river basin (Methods).

| | **Area** | | **N** | | **ELA** | **ELA std** | **AAR** | **AAR std** | **AAR <0.2** | **AAR >0.7** | **Implied ΔV** | **Implied ΔV unc** | **BalAbl** | **BalAbl unc** | **TotAbl** | **TotAbl unc** |
|---|---|---|---|---|---|---|---|---|---|---|---|---|---|---|---|---|
| ***Basin*** | $km^2$ | % | - | % | m a.s.l. | m a.s.l. | - | - | % | % | % | % | % | % | $Gt\ a^{-1}$ | $Gt\ a^{-1}$ |
| *Lake Balkash* | 1343 | 47% | 256 | 88% | 4057 | 280 | 0.37 | 0.27 | 47% | 8% | -42% | 7% | 43% | 10% | 2.06 | 0.27 |
| *Syr Darya* | 682 | 29% | 145 | 70% | 4210 | 319 | 0.48 | 0.33 | 42% | 21% | -27% | 3% | 59% | 17% | 1.54 | 0.26 |
| *Gobi Interior* | 714 | 31% | 118 | 61% | 4248 | 487 | 0.39 | 0.29 | 53% | 12% | -38% | 7% | 45% | 11% | 2.05 | 0.27 |
| *Amu Darya* | 2153 | 20% | 471 | 53% | 4885 | 492 | 0.51 | 0.30 | 30% | 23% | -20% | 2% | 66% | 40% | 5.45 | 1.95 |
| *Tarim Interior* | 11826 | 43% | 1540 | 69% | 5258 | 574 | 0.66 | 0.32 | 20% | 48% | -12% | 1% | 78% | 48% | 10.74 | 4.12 |
| *Tibetan Plateau Interior* | 1066 | 40% | 143 | 48% | 5934 | 230 | 0.42 | 0.31 | 44% | 14% | -27% | 2% | 47% | 13% | 1.28 | 0.18 |
| *Yangtze* | 983 | 45% | 111 | 55% | 5739 | 206 | 0.45 | 0.30 | 44% | 13% | -25% | 4% | 55% | 15% | 1.40 | 0.21 |
| *Indus* | 9283 | 34% | 1398 | 72% | 5243 | 579 | 0.50 | 0.31 | 35% | 22% | -26% | 3% | 65% | 23% | 17.8 | 3.64 |
| *Ganges - Bramaputra* | 8119 | 44% | 1248 | 72% | 5701 | 507 | 0.37 | 0.27 | 42% | 11% | -43% | 8% | 48% | 9% | 18.3 | 1.62 |
| *Salween* | 431 | 34% | 61 | 69% | 5549 | 295 | 0.32 | 0.25 | 52% | 8% | -60% | 13% | 40% | 12% | 1.48 | 0.25 |
| *Mekong* | 49 | 21% | 11 | 61% | 5331 | 305 | 0.40 | 0.33 | 55% | 18% | -45% | 12% | 49% | 25% | 0.20 | 0.059 |

Supplementary Table 5. Comparison of our results for balance portion of glacier ablation to previous studies. Illi combines Balkhash and Gobi in our results. Bahmaputra and Ganges combined in our results. Amu Darya and Syr Darya combined as Aral in [64]. Chu/Issyk-Kul from [64] is within Syr Darya in our results.

| | **Our results** | | | **Shean et al (2020)** [65] | | | **Pritchard (2019)** [64] | | | **Gardelle et al (2013)** [14] | | |
|---|---|---|---|---|---|---|---|---|---|---|---|---|
| *Period* | *2000-2016* | | | *2000-2018* | | | *drought year, 1951-2007* | | | *1999-2011* | | |
| *Imbalance* | *Brun et al (2017)* [63] | | | *Shean et al (2020)* | | | *Brun et al (2017)* | | | *Gardelle et al (2013)* | | |
| *Balance* | *SMB inversion* | | | *Rounce et al (2020)* [66] | | | *Updated Kaser (2010)* | | | *Kaser (2010)* [67] | | |
| | Total (Gt) | Imbalance (Gt) | Balance ratio | Total (Gt) | Imbalance (Gt) | Balance ratio | Total (Gt) | Imbalance (Gt) | Balance ratio | Total (Gt) | Imbalance (Gt) | Balance ratio |
| *Amu Darya* | 5.5 | 1.8 | 66% | 10.4 | 1.32 | 88% | 7.7* | 1.2 | 84% | - | - | - |
| *Brahmaputra* | 18.3 | 9.5 | 48% | 22.5 | 5.23 | 77% | 5.6 | 4.9 | 10% | 5.7 | 4.6 | 18% |
| *Ganges* | | | | 11.6 | 3.26 | 71% | 3.3 | 2.6 | 20% | 4.7 | 3.3 | 31% |
| *Ili* | 4.1 | 2.3 | 44% | 3.9 | 2.00 | 47% | 1.7 | 1.1 | 40% | - | - | - |
| *Indus* | 17.8 | 6.2 | 65% | 26.0 | 4.55 | 82% | 14.9 | 4.2 | 71% | 6.6 | 3.3 | 51% |
| *Inner TP* | 1.3 | 0.7 | 47% | 3.3 | 1.12 | 60% | - | - | - | - | - | - |
| *Inner TP ext* | - | - | - | 2.1 | 0.70 | 65% | - | - | - | - | - | - |
| *Mekong* | 0.2 | 0.1 | 49% | 0.3 | 0.11 | 62% | - | - | - | - | - | - |
| *Salween* | 1.5 | 0.9 | 40% | 2.0 | 0.75 | 63% | - | - | - | - | - | - |
| *Syr Darya* | 1.5 | 0.6 | 59% | 2.6 | 0.43 | 84% | * | * | * | - | - | - |
| *Tarim* | 10.7 | 2.4 | 78% | 11.4 | 2.40 | 74% | 3.6 | 0 | 100% | - | - | - |
| *Yangtze* | 1.4 | 0.6 | 55% | 2.6 | 0.76 | 70% | - | - | - | - | - | - |
| ***Total*** | **62.3** | **25.2** | **60%** | **98.7** | **22.6** | **81%** | **36.9** | **14.0** | **62%** | **-** | **-** | **-** |

Supplementary Table 6. Density assumptions for the *dH/dt* signal in Equation 1. Each assumed density has an uncertainty $\sigma_\rho$ of approximately 60 kg m-3 [68].

| | Emergence > 0 ($\nabla \cdot \mathbf{q} < 0$) | Emergence <= 0 ($\nabla \cdot \mathbf{q} >= 0$) |
|---|---|---|
| *dH/dt* >= 0 | Dynamic input, possibly mixed signal; if abs(*dH/dt*)>abs($\nabla \cdot \mathbf{q}$), implies accumulation: $\rho_{dH}$ = 600 kg $m^{-3}$ if abs(*dH/dt*)<abs($\nabla \cdot \mathbf{q}$), implies ablation and/or mixed signal: $\rho_{dH}$ = 850 kg $m^{-3}$ | Annual accumulation; $\rho_{dH}$= 600 kg $m^{-3}$ |
| *dH/dt* < 0 | Annual ablation; $\rho_{dH}$ = 900 kg $m^{-3}$ | Dynamic losses, possibly also mixed signal; if abs(*dH/dt*)>abs($\nabla \cdot \mathbf{q}$), implies net ablation: $\rho_{dH}$ = 900 kg $m^{-3}$ If abs(*dH/dt*)<abs($\nabla \cdot \mathbf{q}$), implies mixed ablation and accumulation: $\rho_{dH}$= 850 kg $m^{-3}$ |

## SUPPLEMENTARY FIGURES

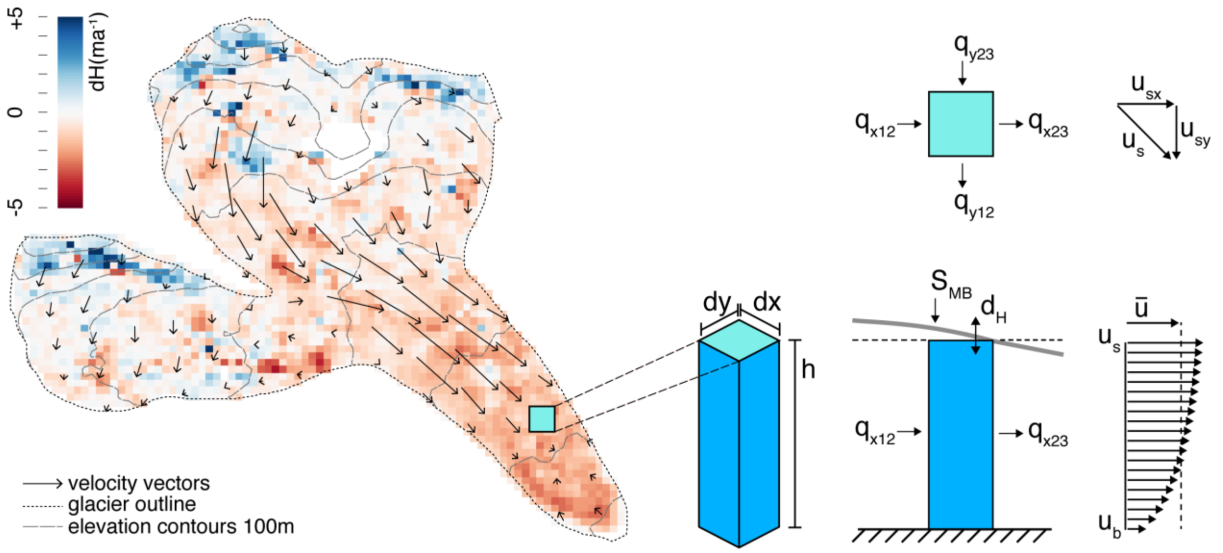


Supplementary Figure 1. Depiction of principal steps in workflow for calculating ice flux and specific mass balance on a pixel basis according to Equation 1. The glacier depicted is Glacier 354 (reoriented).

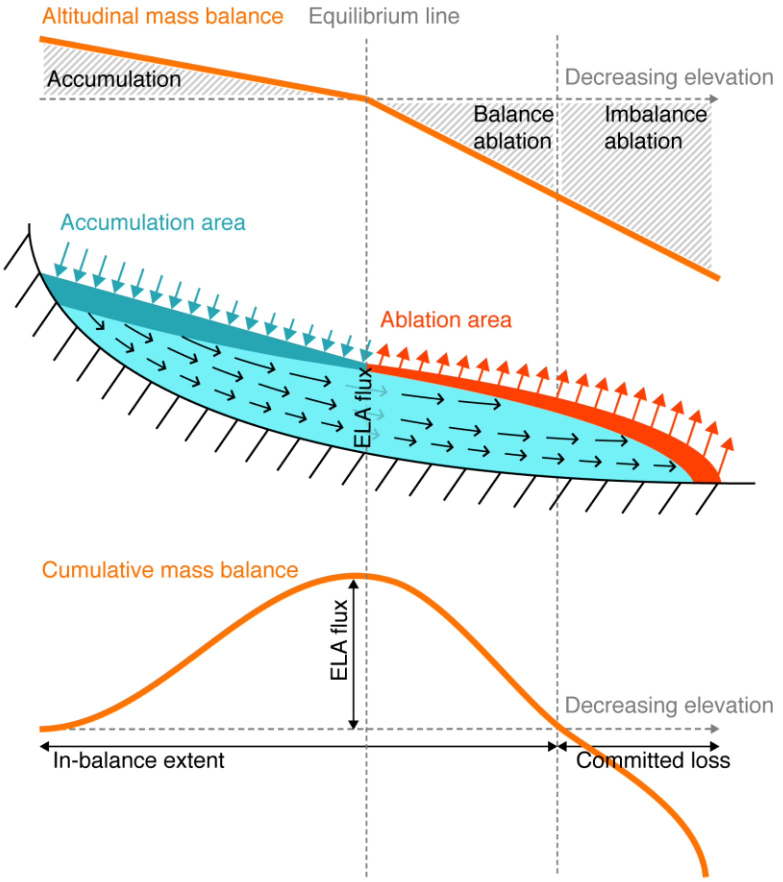


Supplementary Figure 2. Depiction of balance ablation calculation. Note that the 'committed loss' idealised here depicts the area of the glacier unsupported by accumulation in the contemporary mass balance regime, which corresponds to the source area of 'imbalance ablation'. Our calculation of implied mass change additionally accounts for changes in glacier geometry in response to the mass budget's imbalance.

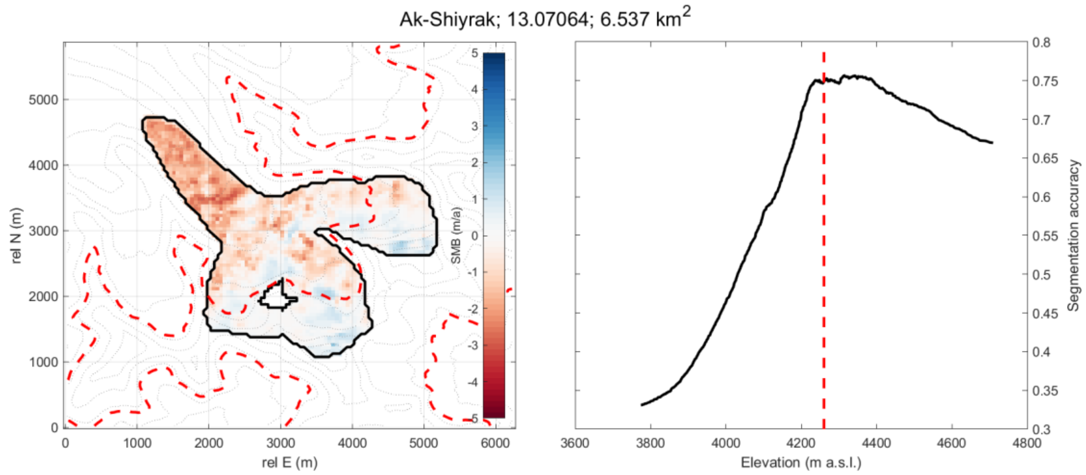


Supplementary Figure 3. ELA determination for Glacier 354 (RGIID 13.07064), showing gridded mass balance data and 100 m elevation contours (left) and the segmentation accuracy with respect to elevation (right). For both plots the derived ELA is shown as a dashed red line. For this glacier we derive an ELA of 4260 m a.s.l. and an AAR of 0.38.

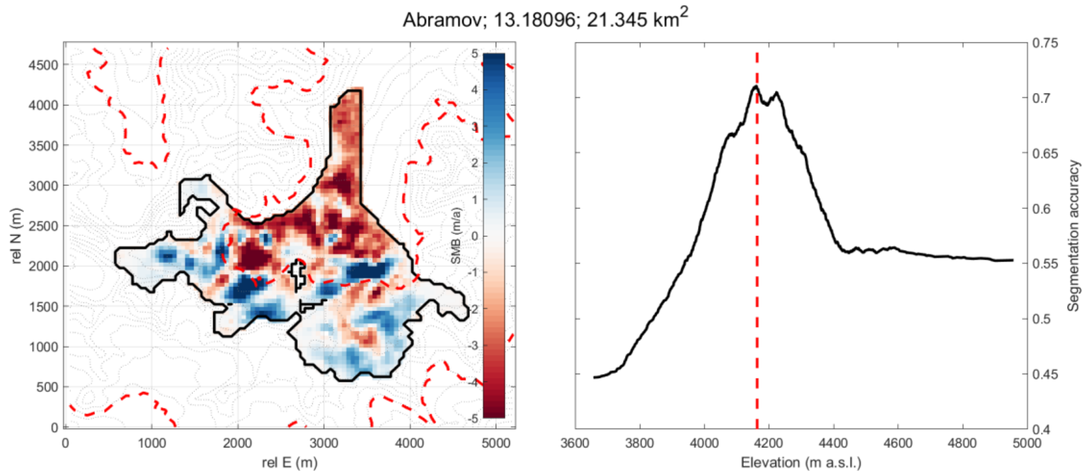


Supplementary Figure 4. ELA determination for Abramov Glacier (RGIID 13.18096), showing gridded mass balance data and 100 m elevation contours (left) and the segmentation accuracy with respect to elevation (right). For both plots the derived ELA is shown as a dashed red line. For this glacier we derive an ELA of 4163 and an AAR of 0.59.

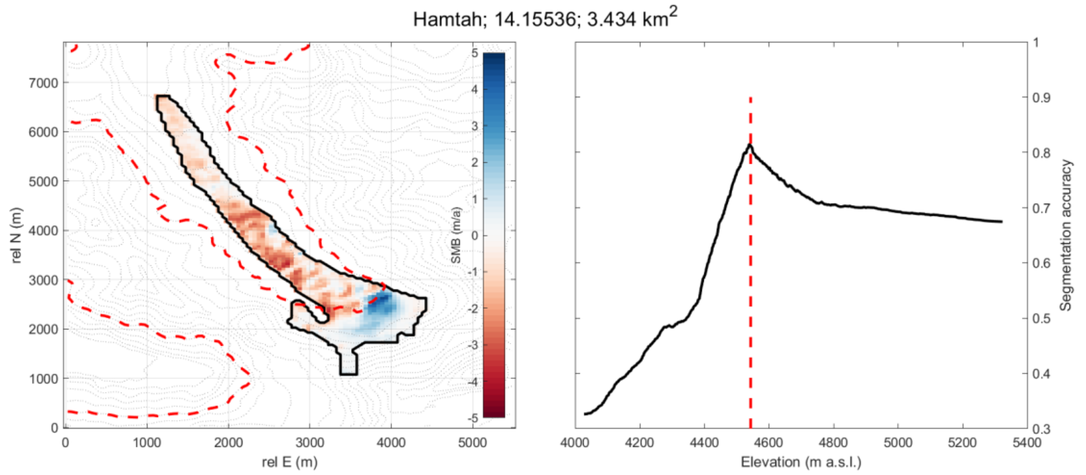


Supplementary Figure 5. ELA determination for Hamtah Glacier (RGIID 14.15536), showing gridded mass balance data and 100 m elevation contours (left) and the segmentation accuracy with respect to elevation (right). For both plots the derived ELA is shown as a dashed red line. For this glacier we derive an ELA of 4543 m a.s.l. and an AAR of 0.35.

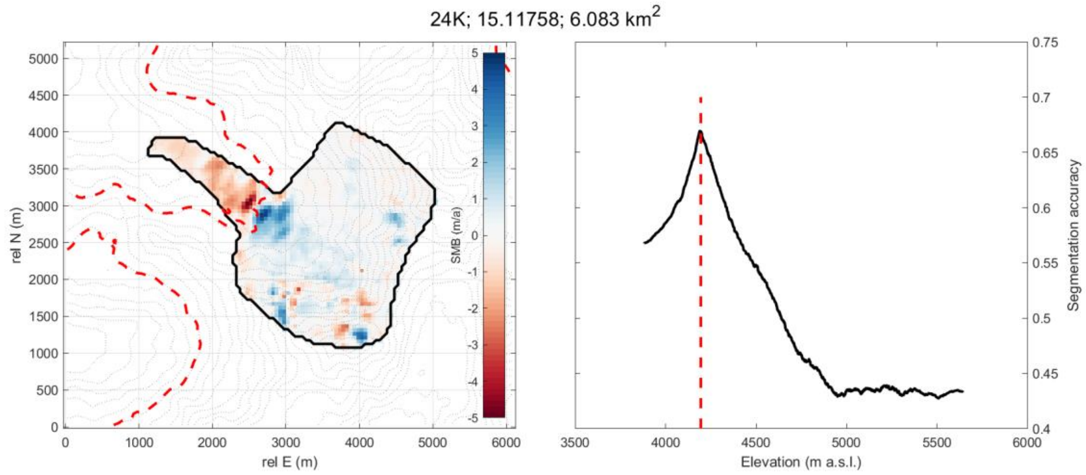


Supplementary Figure 6. ELA determination for 24K Glacier (RGIID 15.11758), showing gridded mass balance data and 100 m elevation contours (left) and the segmentation accuracy with respect to elevation (right). For both plots the derived ELA is shown as a dashed red line. For this glacier we derive an ELA of 4194 and an AAR of 0.87.

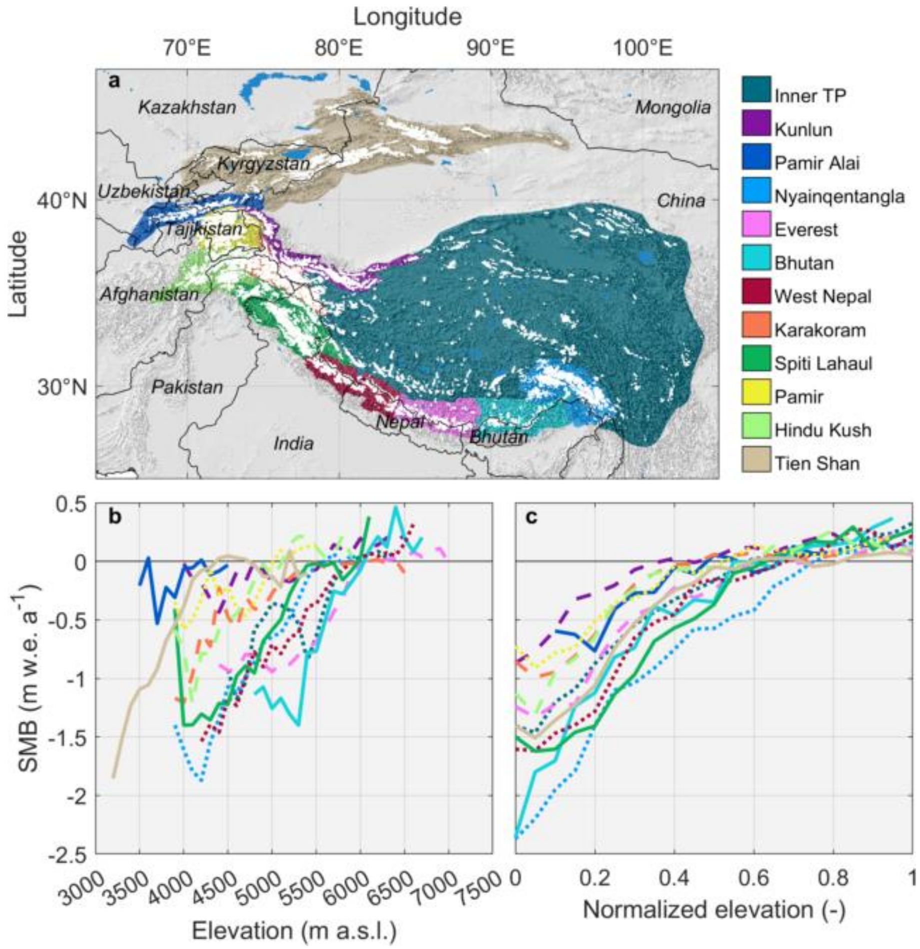


Supplementary Figure 7. Context of glaciers in High Mountain Asia (a), along with mean specific mass balance (SMB) profiles for the 2000-2016 period for each subregion, shown relative to elevation (b) and normalized to each glacier's elevation range (c). The profiles are area-weighted averages for all elevations bins with glacier area > 10 km2. Line styles are varied only to differentiate between the lines. Uncertainty and *dH/dt* signals are shown for each subregion in Supplementary Figures 11-12. Glaciers are shown in white, with a background hillshade of the GTOPO30 dataset sourced from the USGS (https://doi.org/10.5066/F7DF6PQS).

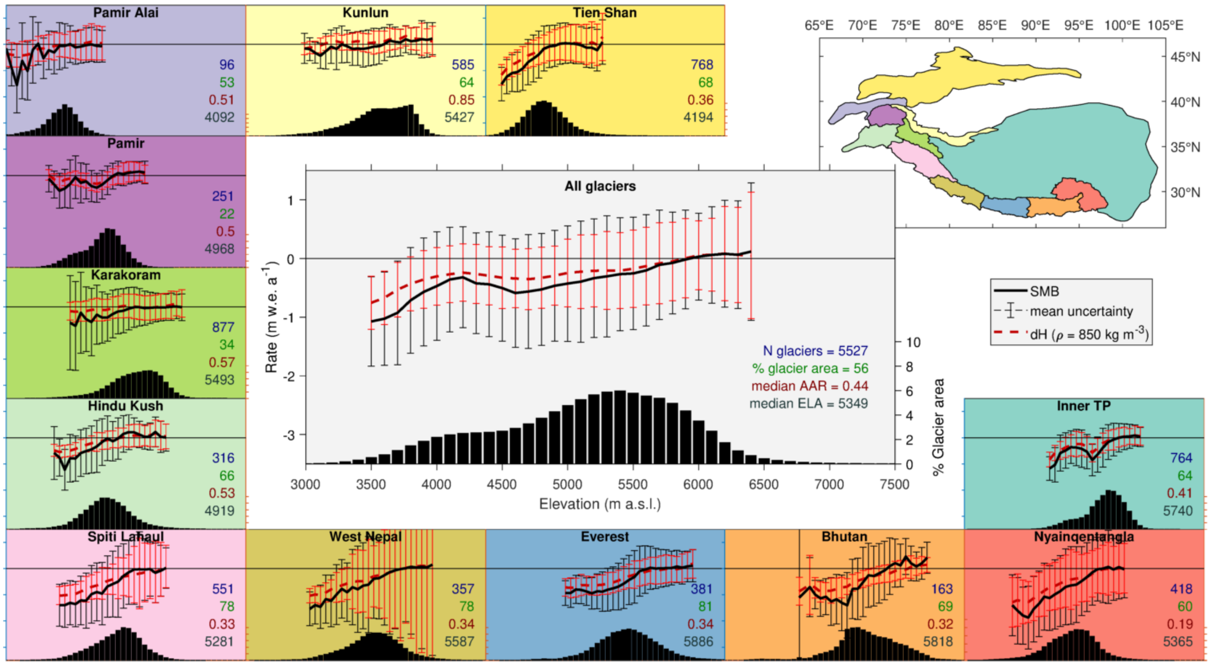


Supplementary Figure 8. Regional and subregional specific mass balance versus elevation. Note that the error bars correspond to the weighted mean uncertainty, not the uncertainty of the mean. The profiles are area-weighted averages for all elevations bins with glacier area > 10 km$^2$.

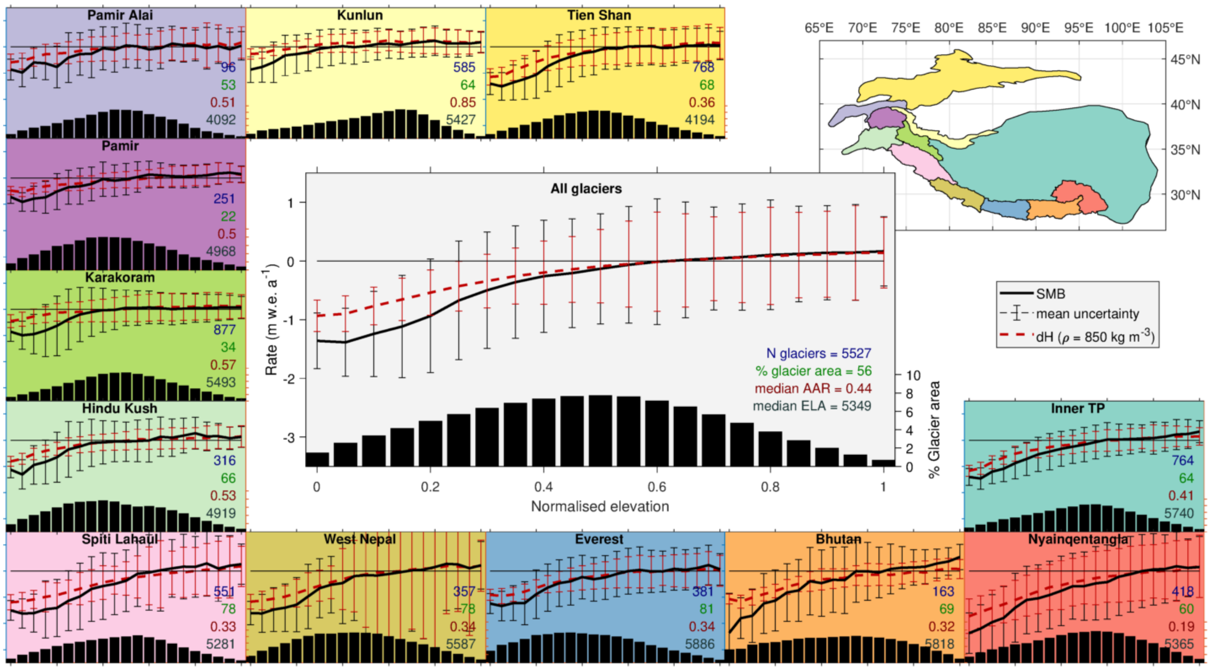


Supplementary Figure 9. Regional and subregional specific mass balance versus normalized elevation. Note that the error bars correspond to the weighted mean uncertainty, not the uncertainty of the mean. The profiles are area-weighted averages for all elevations bins with glacier area > 10 km$^2$.

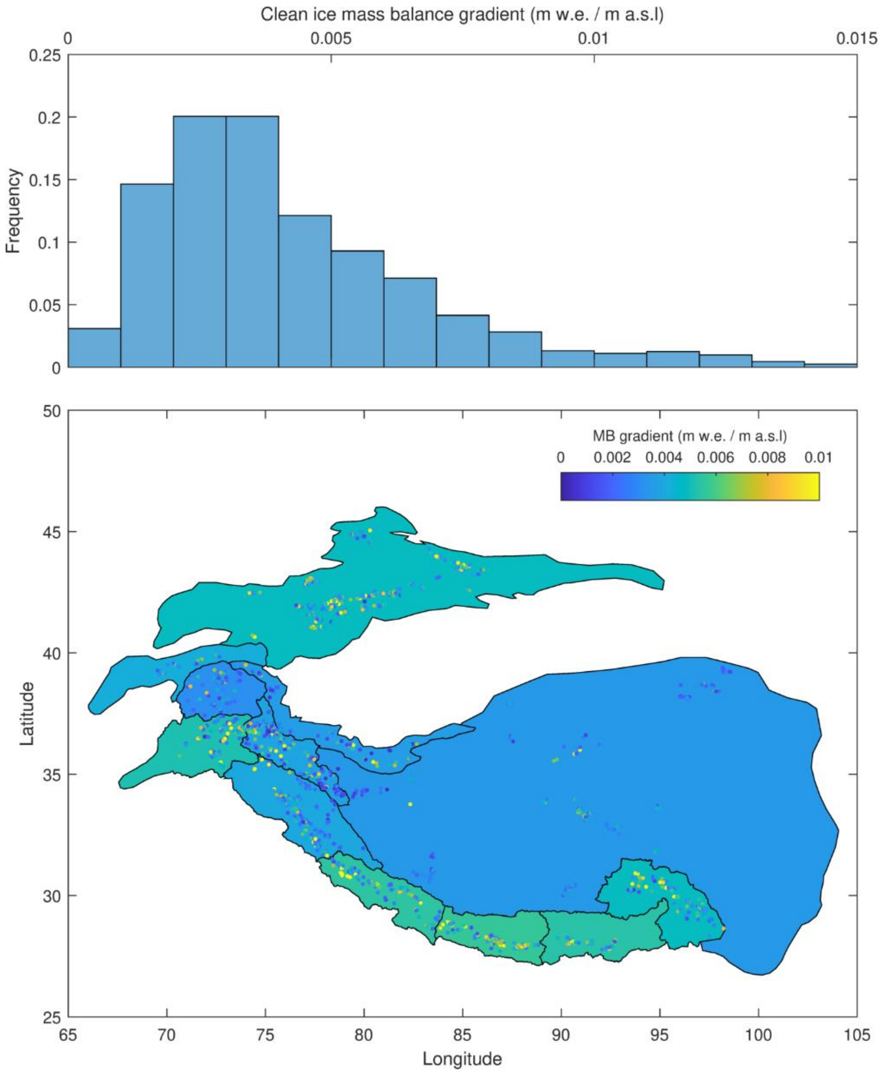


Supplementary Figure 10. Distribution of derived clean-ice ablation-area mass balance gradients, showing results for individual glaciers with $R^2$>0.8. The mean ablation area mass balance gradient for these glaciers is 0.0043 m w.e. (m a.s.l.)$^{-1}$, and the area-weighted mean is 0.0055 m w.e. (m a.s.l.)$^{-1}$. We find considerable variability between adjacent glaciers due to each glacier's unique setting. At the regional scale, the values follow a lognormal distribution with μ = -2.45 and σ = 0.29. Also shown are the subregional mean values, which show slightly higher ablation gradients in the Himalayas, Tien Shan, and Hindu Kush ranges. Note that our results closely match those of [10] which conform to field-derived ablation gradients.

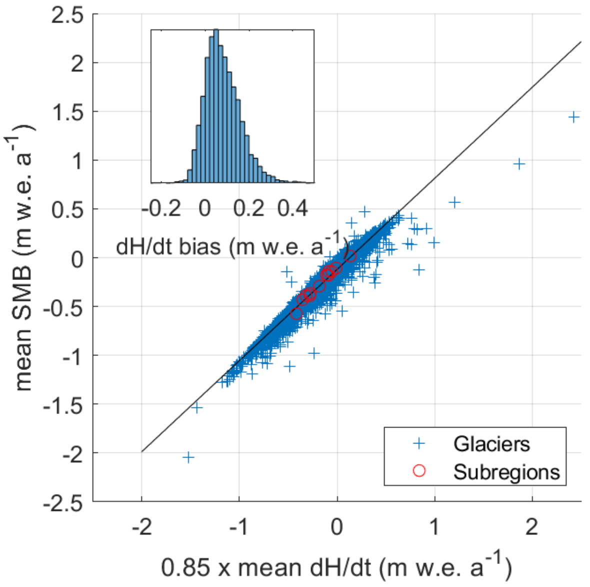


Supplementary Figure 11. Comparison of glacier-wide mass balances computed using thinning data and a density of 850 kg $km^3$ as in [63] and our results accounting for ice dynamics and density differences in accumulation and ablation areas (Supplementary Table 6). The median bias is of the *dH/dt* result is +0.07 m w.e.$a^{-1}$ due to an overestimation of mass accumulation. This comparison is also performed subregionally (see Figure 1).

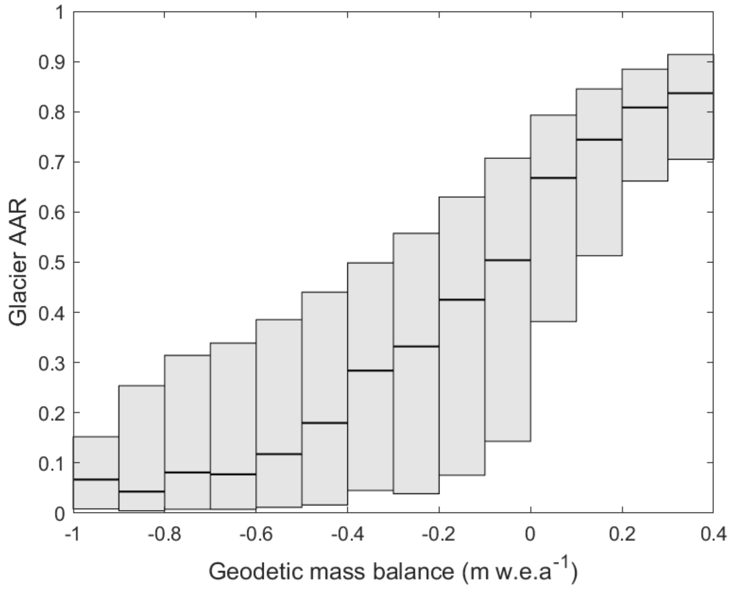


Supplementary Figure 12. Boxplot depicting the relationship of mean geodetic mass balance (calculated with the mean SMB from our results) to Accumulation Area Ratio (AAR) for our subset of analysed glaciers. Boxes correspond to the 25th and 75th centile values in each range, while the black line depicts the median.

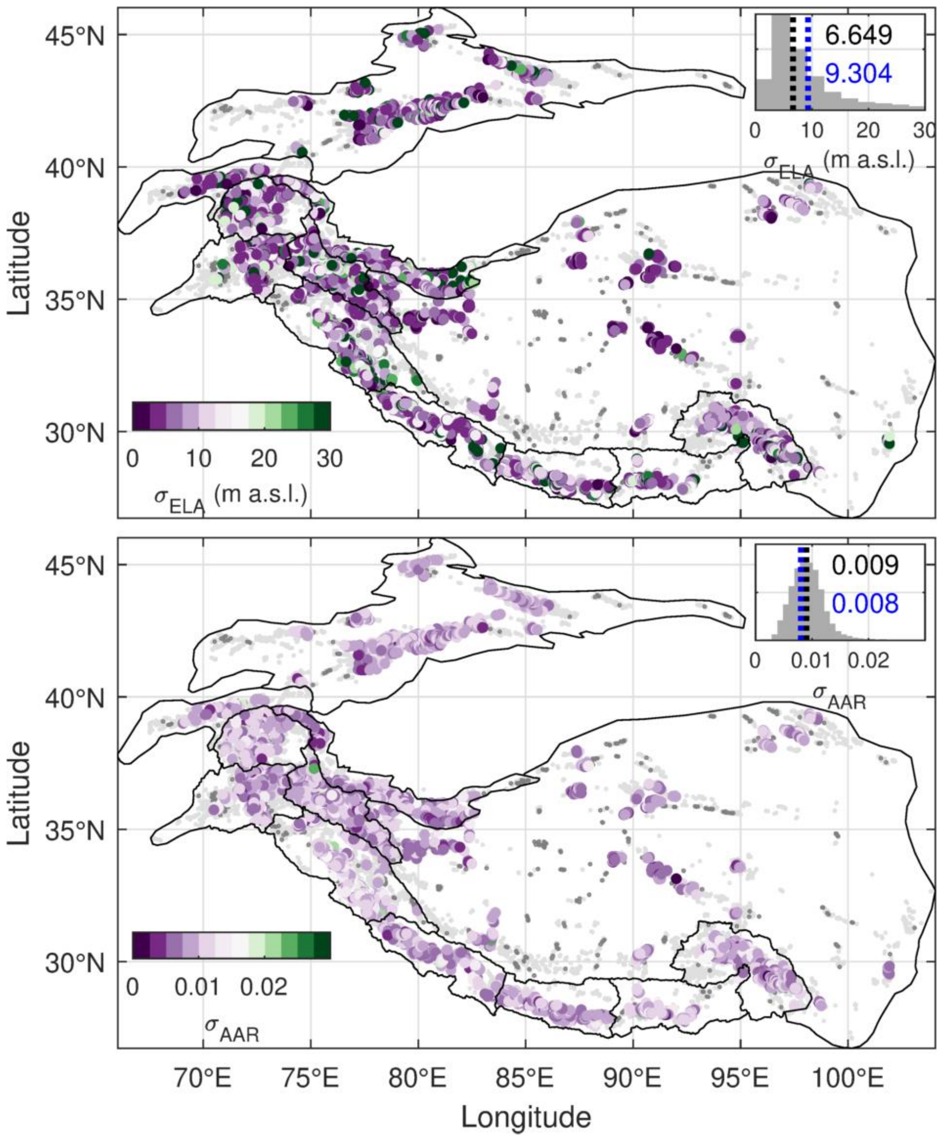


Supplementary Figure 13. Glacier-specific uncertainty of ELAs and AARs derived from the Monte Carlo analysis, also indicating regional median (black) and area-weighted mean (blue) values of each. Surprisingly little change occurs with perturbed input data (see Methods). This is likely an uncertainty underestimation for both parameters as it assumes that SMB errors are uncorrelated across the glacier surface.

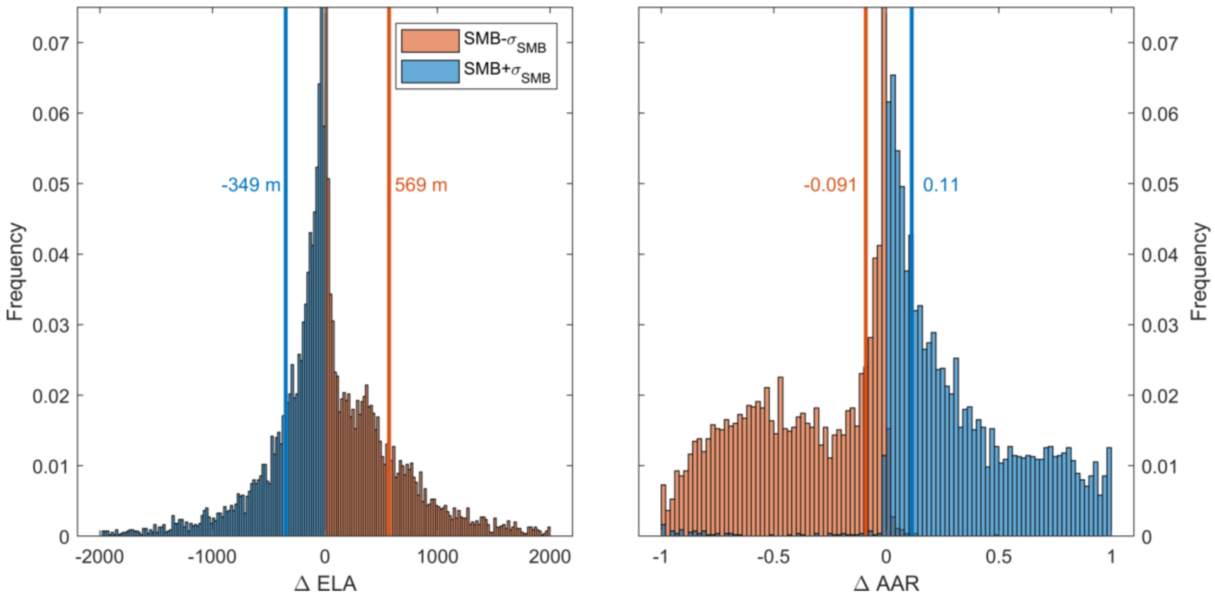


Supplementary Figure 14. Distribution of ELA and AAR uncertainties based on a systematic 1-sigma increase or decrease in SMB, also indicating the 68th centile values. This uncertainty is likely to be conservative, as it assumes that SMB uncertainty is perfectly correlated across the entire glacier. This ELA uncertainty is not symmetrical, as uncertainty in accumulation areas is higher than in ablation areas. Thus, if there were a positive systematic bias in our SMB results (i.e. if the real SMB = SMB - $\sigma_{SMB}$), extensive accumulation areas would be converted to ablation areas. If there were a negative bias in our SMB results (i.e. if the real SMB = SMB + $\sigma_{SMB}$), a small portion of ablation areas would be converted to accumulation areas.

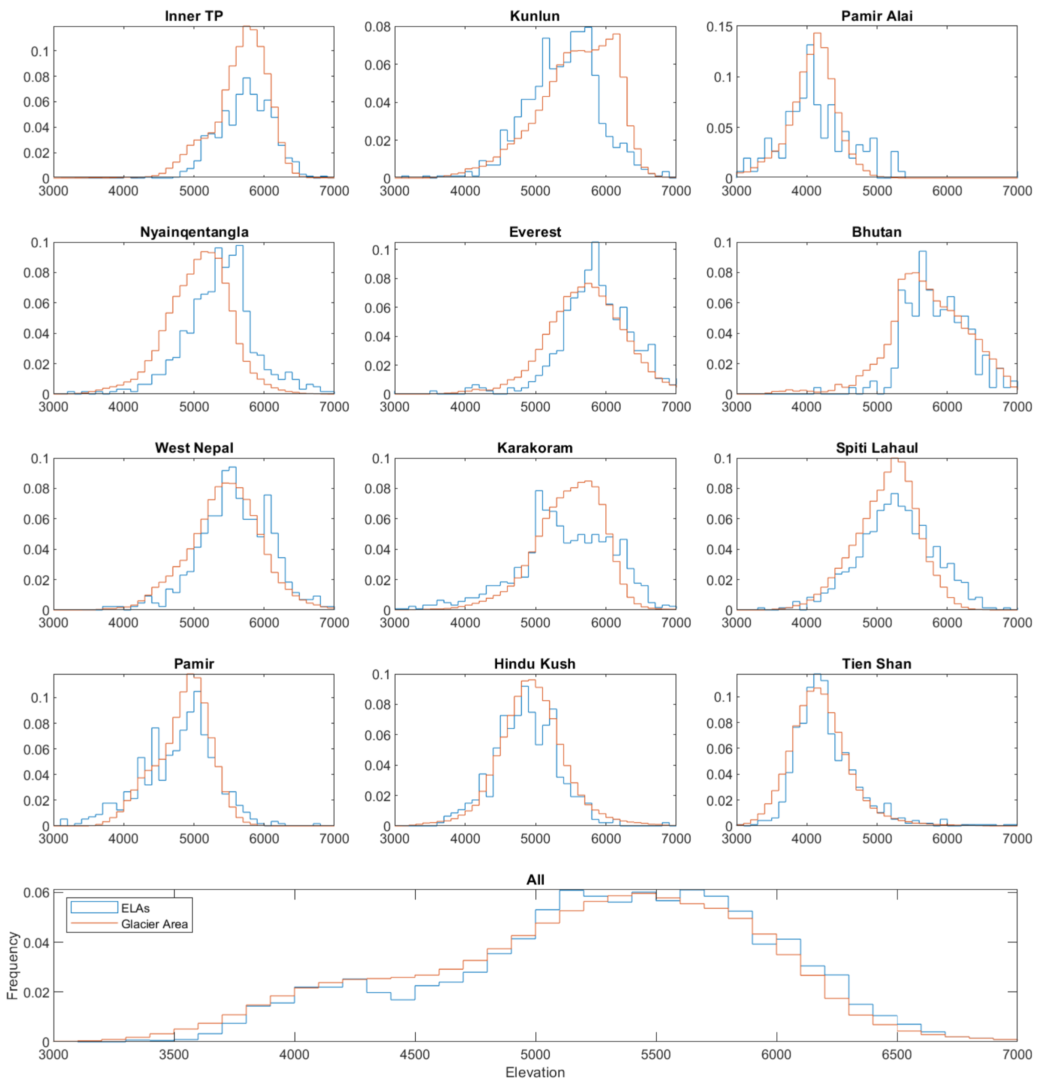


Supplementary Figure 15. Distribution of ELA values relative to glacier hypsometry for each subregion. ELAs generally follow glacier hypsometry, but Pamir, Pamir Alai, Kunlun, and Karakoram subregions show a bias to lower elevations, while Nyainqentangla and Bhutan subregions show a bias to higher elevations.

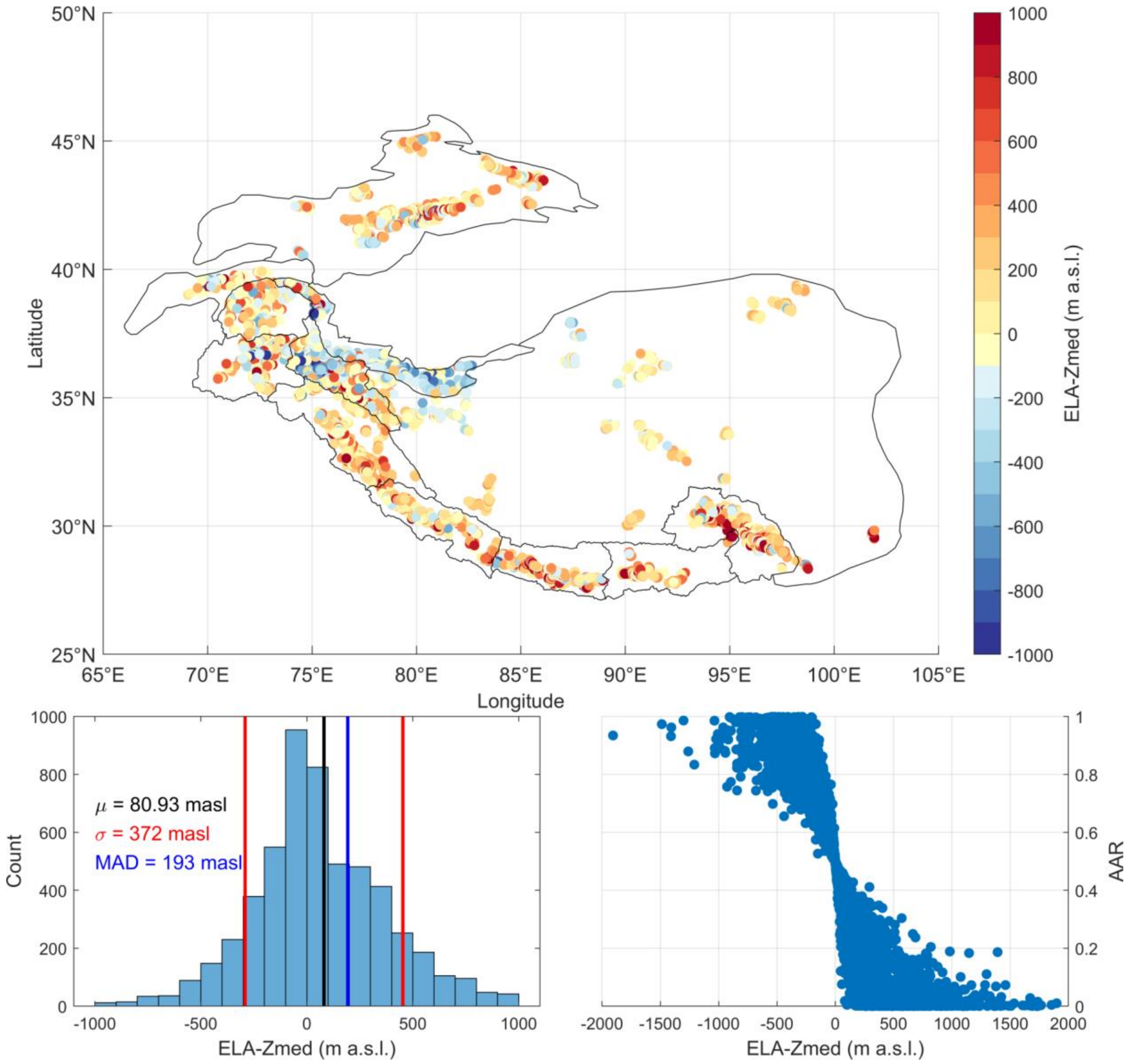


Supplementary Figure 16.The deviation of ELA to median glacier elevation (Zmed) across High Mountain Asia shows a slight bias and skew to higher ELAs but high variability; the resulting median absolute deviation (MAD) is 197 m. The observed pattern is intimately related to AAR.

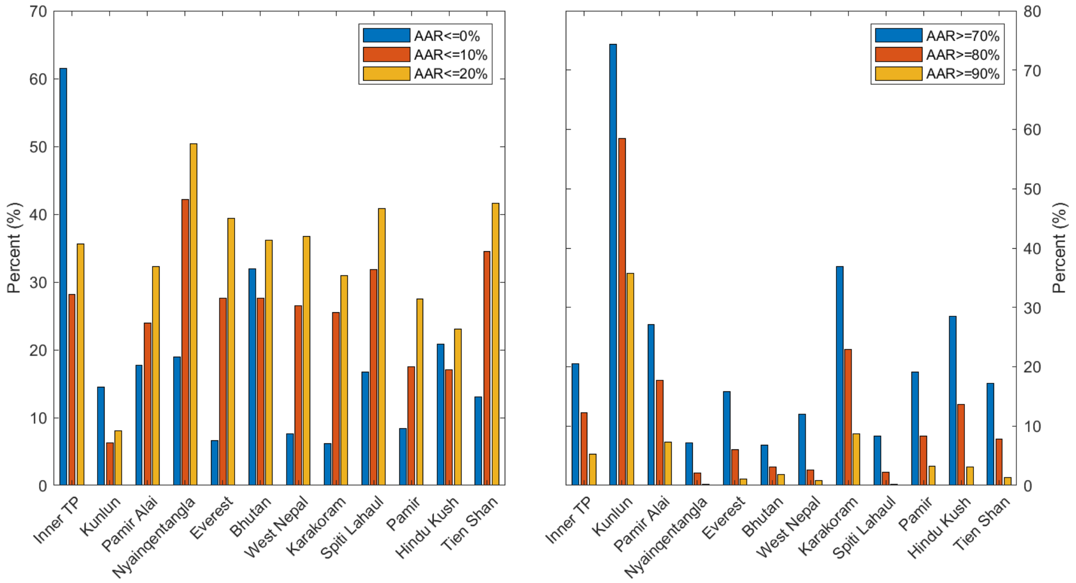


Supplementary Figure 17. Frequency of glaciers with low (left) and high (right) AARs in each subregion.

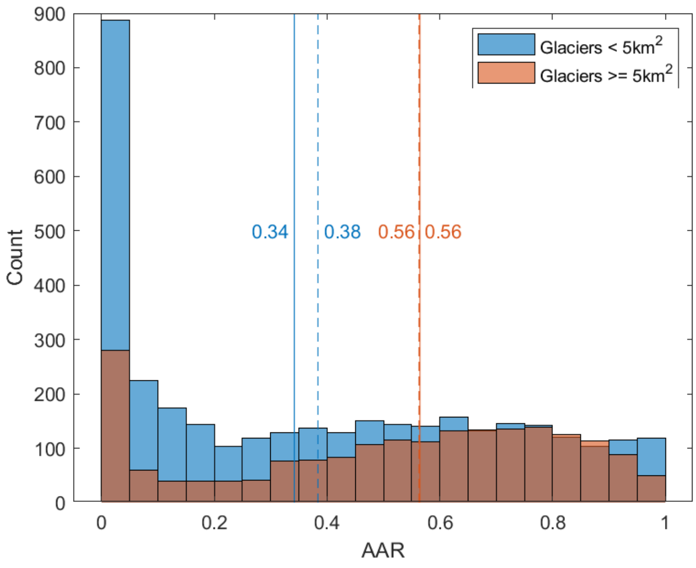


Supplementary Figure 18. Histograms of AAR with respect to glacier size, distinguishing between glaciers larger and smaller than 5 km$^2$. The solid line indicates the population median AAR, while the dashed line indicates the area-weighted mean.

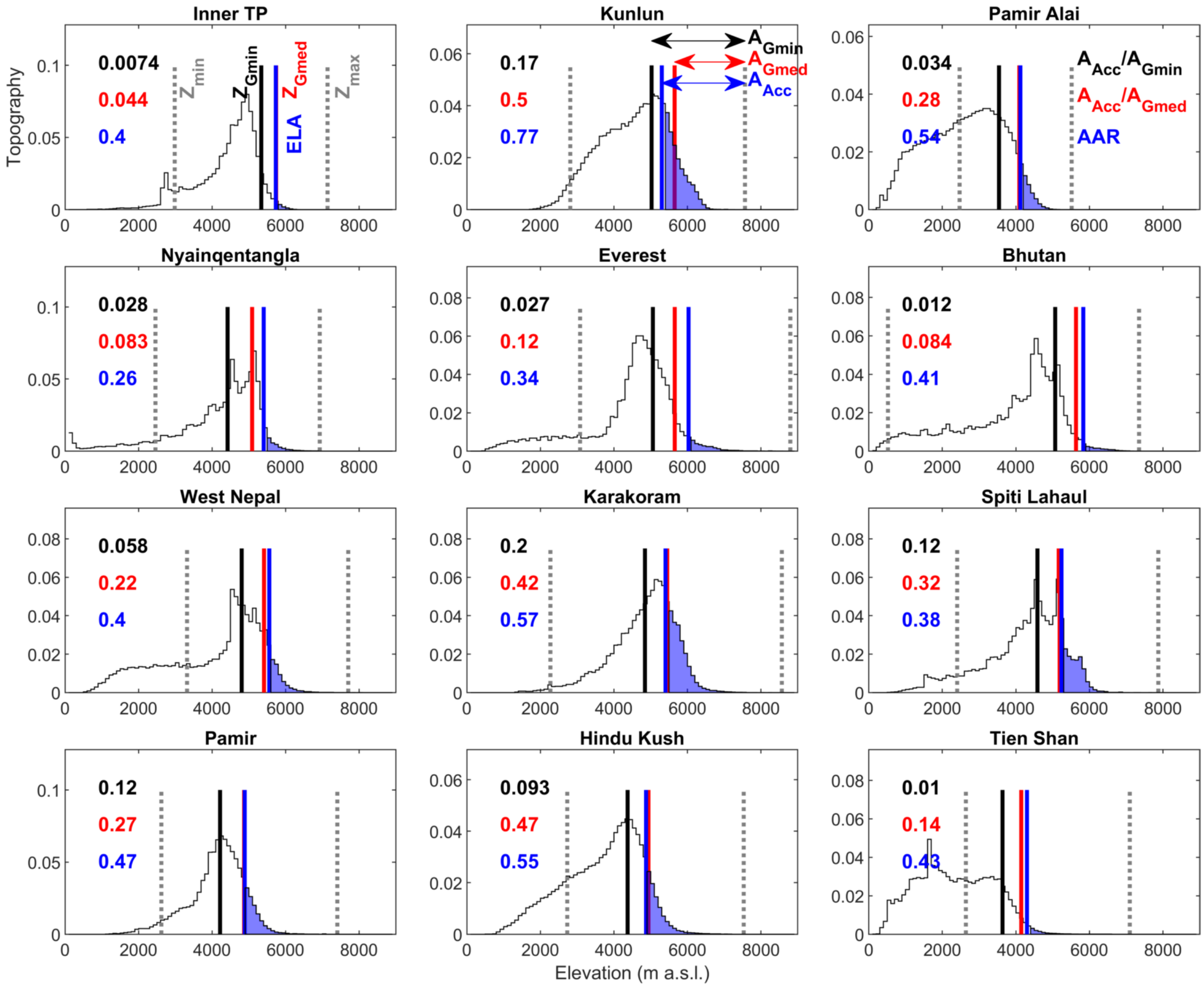


Supplementary Figure 19. Topographic availability for glaciers across HMA, based on hypsometry of each subregion represented in the GTOPO30, showing key elevations for glaciers: minimum and maximum glacier elevations (grey dashed lines), median of glacier minimum elevations (black line), median of glacier median elevations (red line), and ELA (blue line). The blue shaded area corresponds to topography above the regional ELA, and is compared to the total regional area above the median-minimum and median-median glacier elevations (black and red values, respectively). The value in blue is the regional AAR.

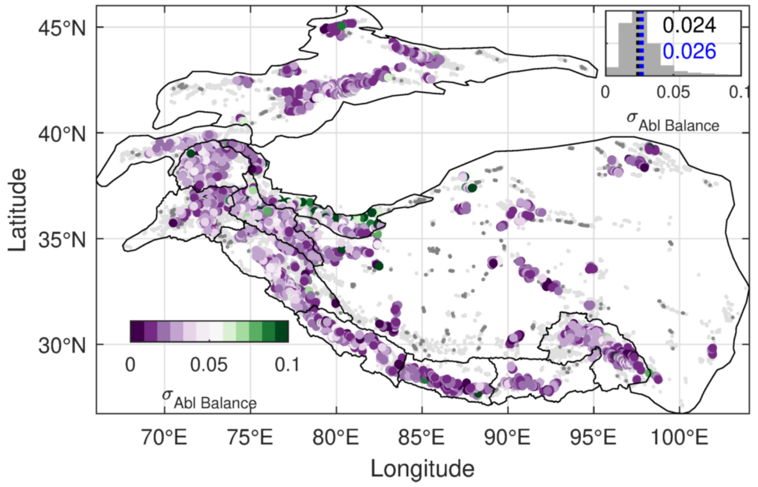


Supplementary Figure 20. Glacier-specific uncertainty of ablation balance ratio derived from the Monte Carlo analysis, also indicating regional median (black) and area-weighted mean (blue) values of each. As with ELA and AAR, this uncertainty estimate may not fully represent systematic errors.

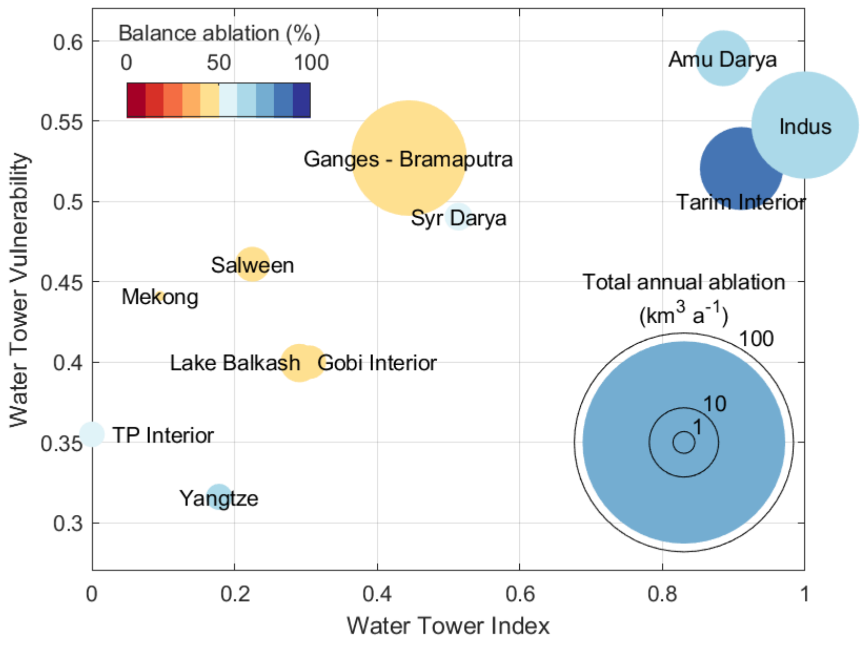


Supplementary Figure 21. Importance of glacier melt in the context of mountain water tower importance (index) and vulnerability [69], depicting river basins by the total annual volume of glacier ablation, and the in-balance portion of ablation which is compensated annually by glacier accumulation.

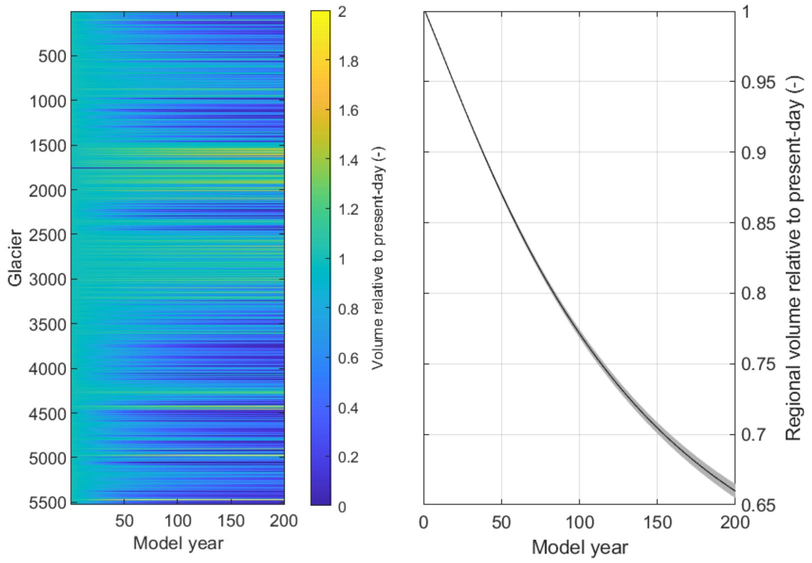


Supplementary Figure 22. Results of baseline scenario of glacier volume change according to observed mass balance regime (Methods) for 5527 individual glaciers and for the total regional modelled volume.

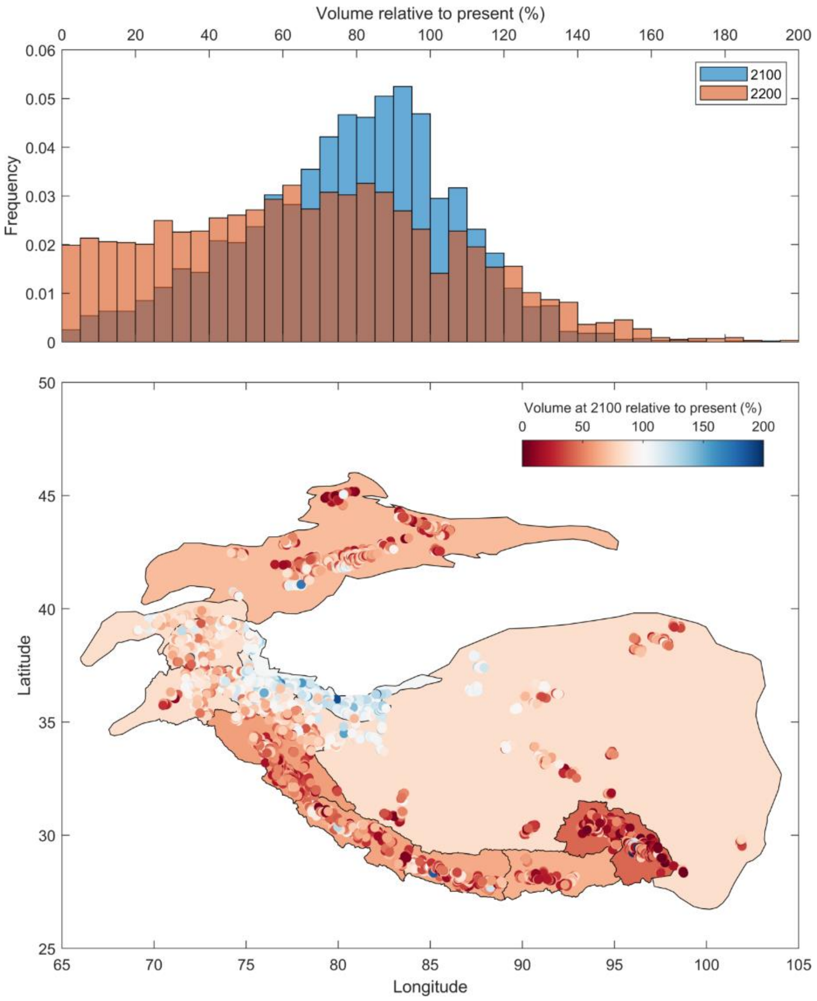


Supplementary Figure 23. Distribution of glacier-specific volume changes by 2100 and 2200 under the recent mass-balance regimes, as well as spatial coherence of subregional volumetric change by 2100 (Supplementary Tables 3-4).

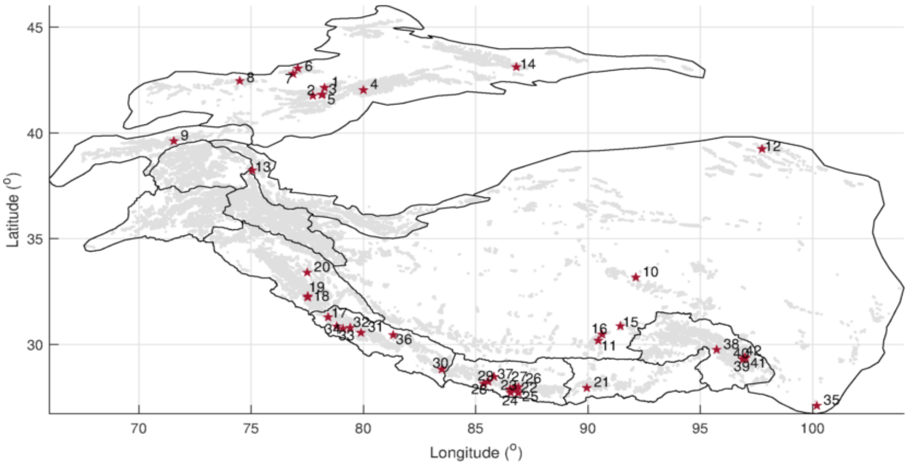


Supplementary Figure 24. Sites for which glaciological mass balance measurements are available encompassing at least one entire year. Numbers correspond to Supplementary Table 1.

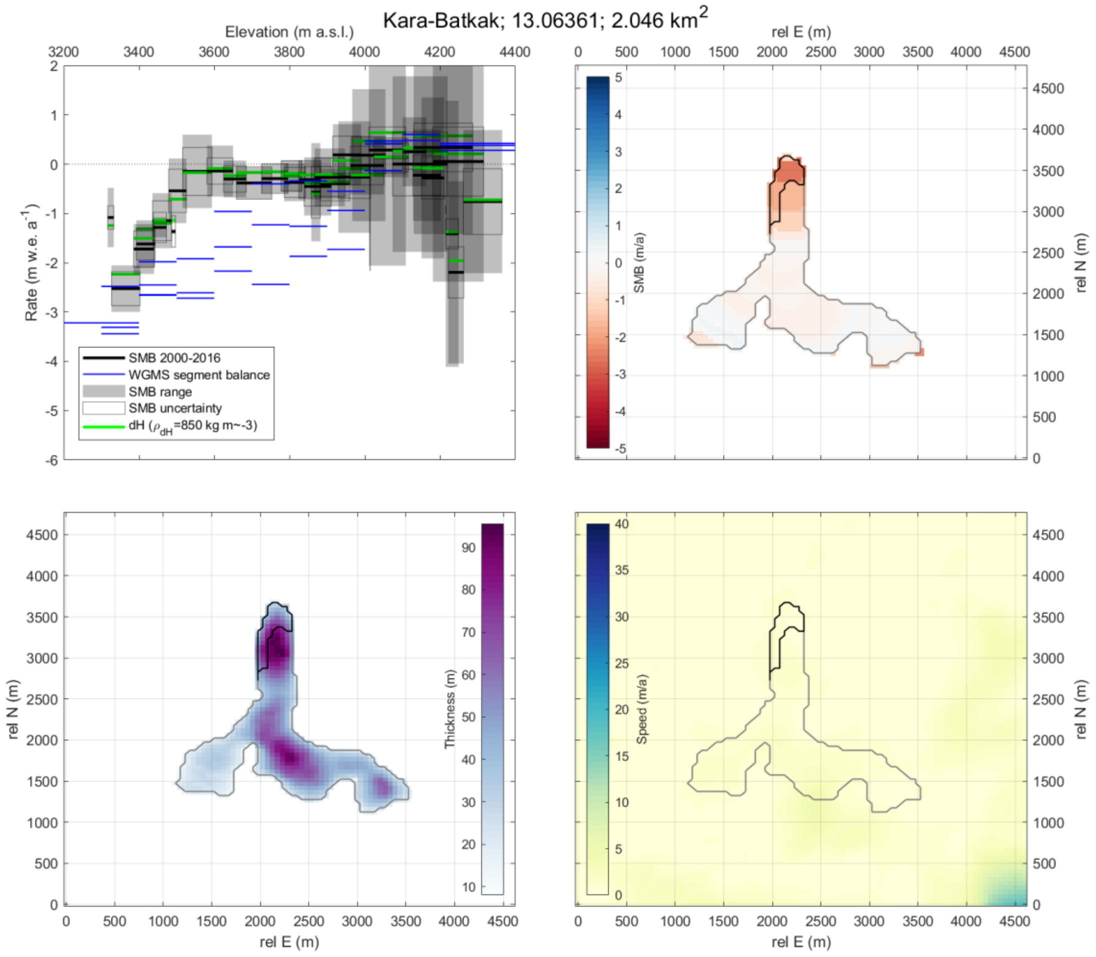


Supplementary Figure 25. Results for glacier 13.06361, as well as inputs and reference mass balance measurements. For this glacier, little ice motion is observable in the satellite observations (lower right), so our method models near-zero ice flux, and our results closely match the surface elevation changes but not the reference mass balance measurements (upper left).

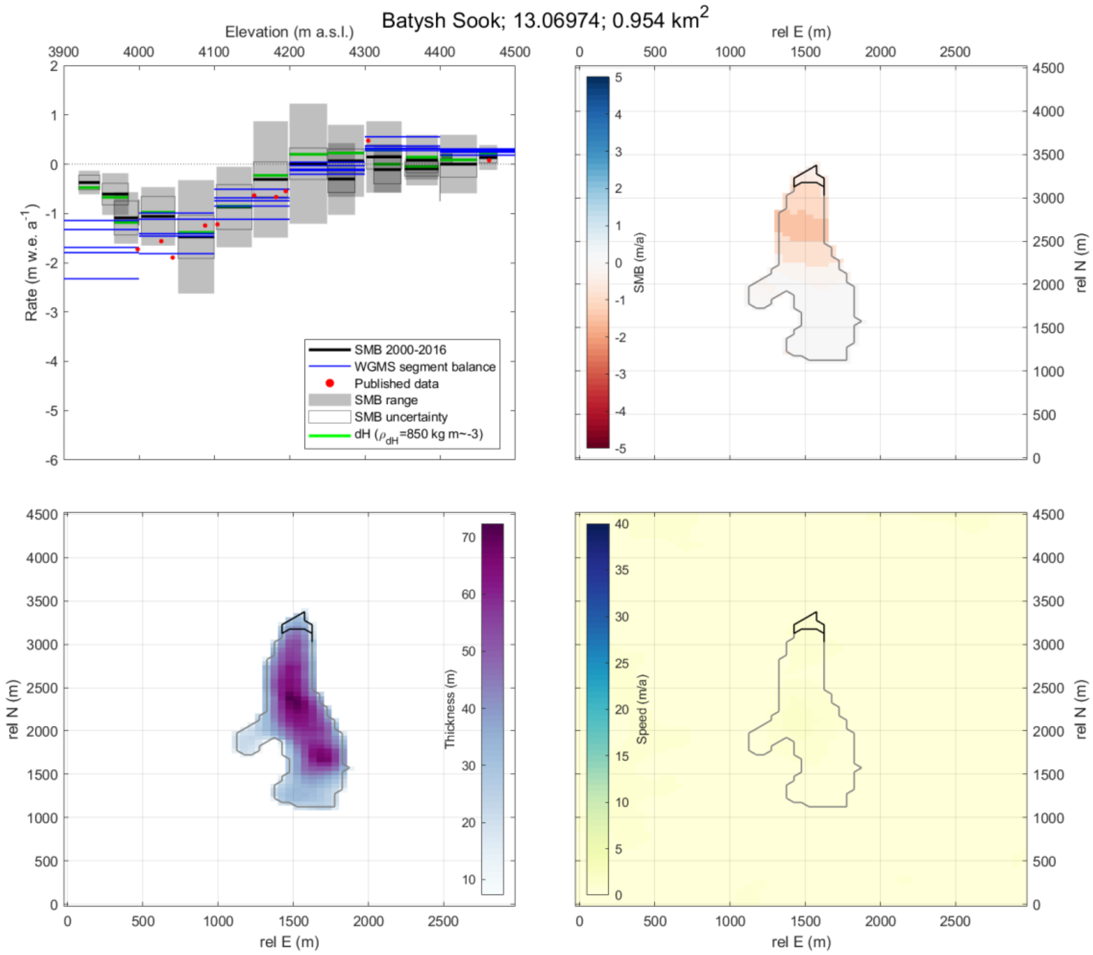


Supplementary Figure 26. Results for glacier 13.06974, as well as inputs and reference mass balance measurements. For this glacier, little ice motion is observable in the satellite observations (lower right), so our method models near-zero ice flux. Our results closely match the surface elevation changes and the reference mass balance measurements (upper left), suggesting that the low flux is correct in this case.

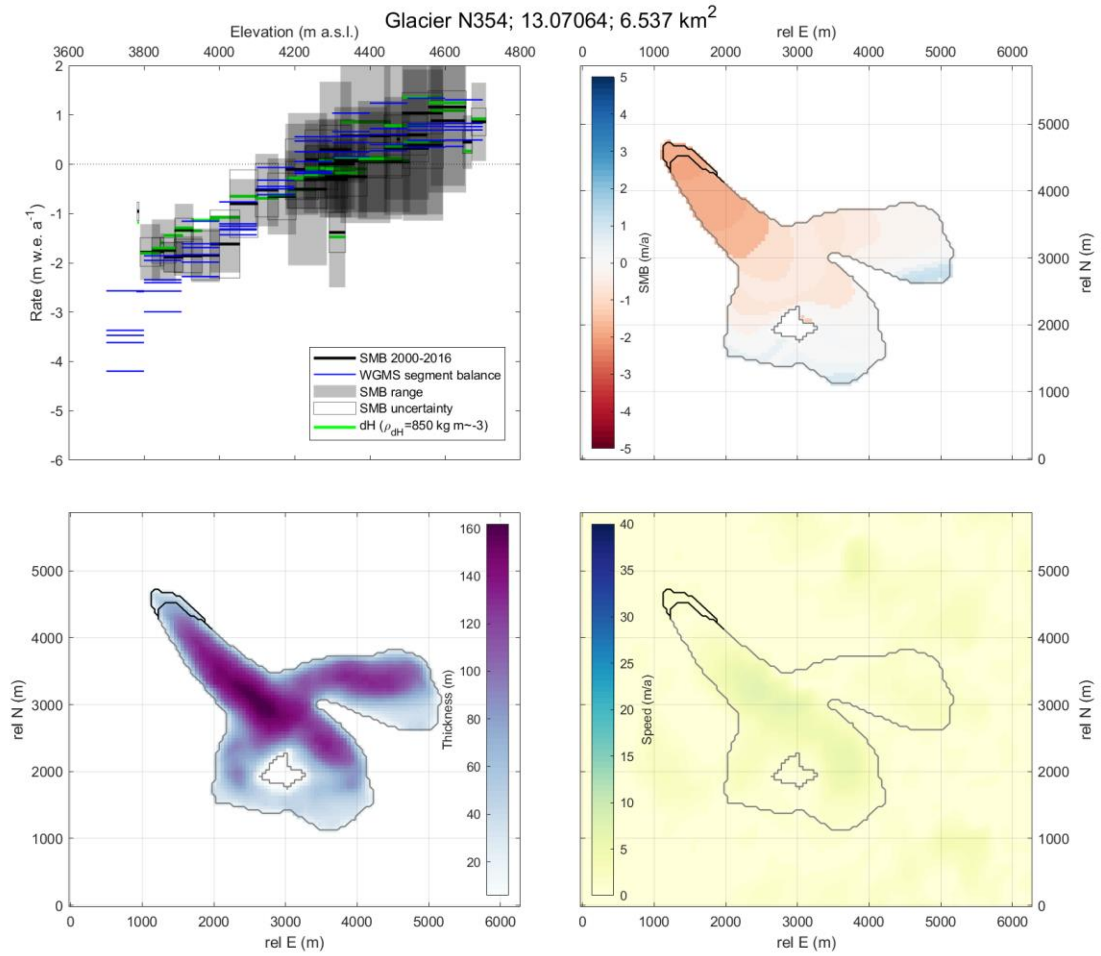


Supplementary Figure 27. Results for glacier 13.07064, as well as inputs and reference mass balance measurements. For this glacier, little ice motion is observable in the satellite observations (lower right), so our method models near-zero ice flux. Our results closely match the surface elevation changes and the reference mass balance measurements (upper left), suggesting that the low flux is correct in this case. The disagreement at lower elevations corresponds to the mapped debris-covered area (black lines, lower right) but this is most likely due to glacier retreat.

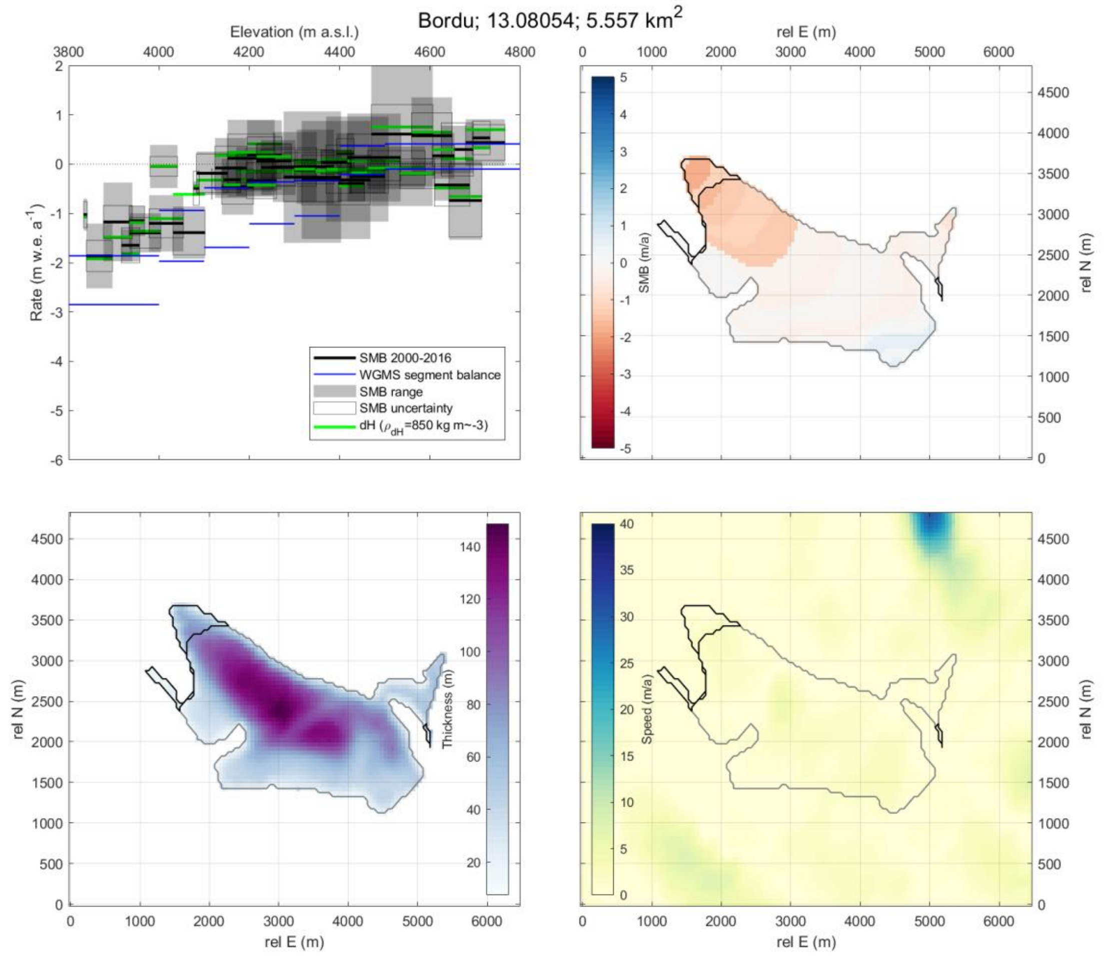


Supplementary Figure 28. Results for glacier 13.08054, as well as inputs and reference mass balance measurements. For this glacier, little ice motion is observable in the satellite observations (lower right), so our method models near-zero ice flux, and our results closely match the surface elevation changes. The reference mass balance measurements indicate considerable variability between years, so it is difficult to tell whether the mismatch is attributable to incorrect ice fluxes or mass balance interannual variability and measurement year bias.

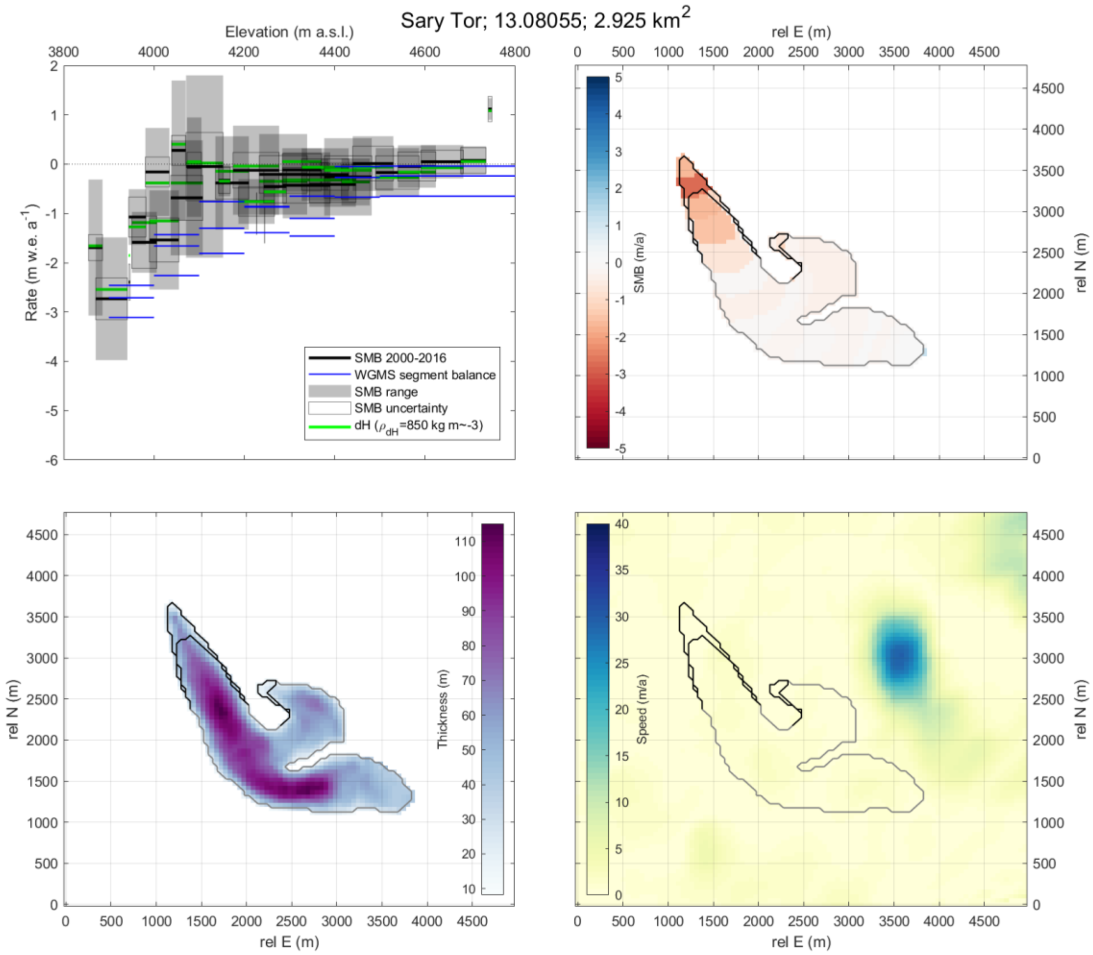


Supplementary Figure 29. Results for glacier 13.08055, as well as inputs and reference mass balance measurements. For this glacier, little ice motion is observable in the satellite observations (lower right), so our method models near-zero ice flux, and our results closely match the surface elevation changes but not the reference mass balance measurements (upper left).

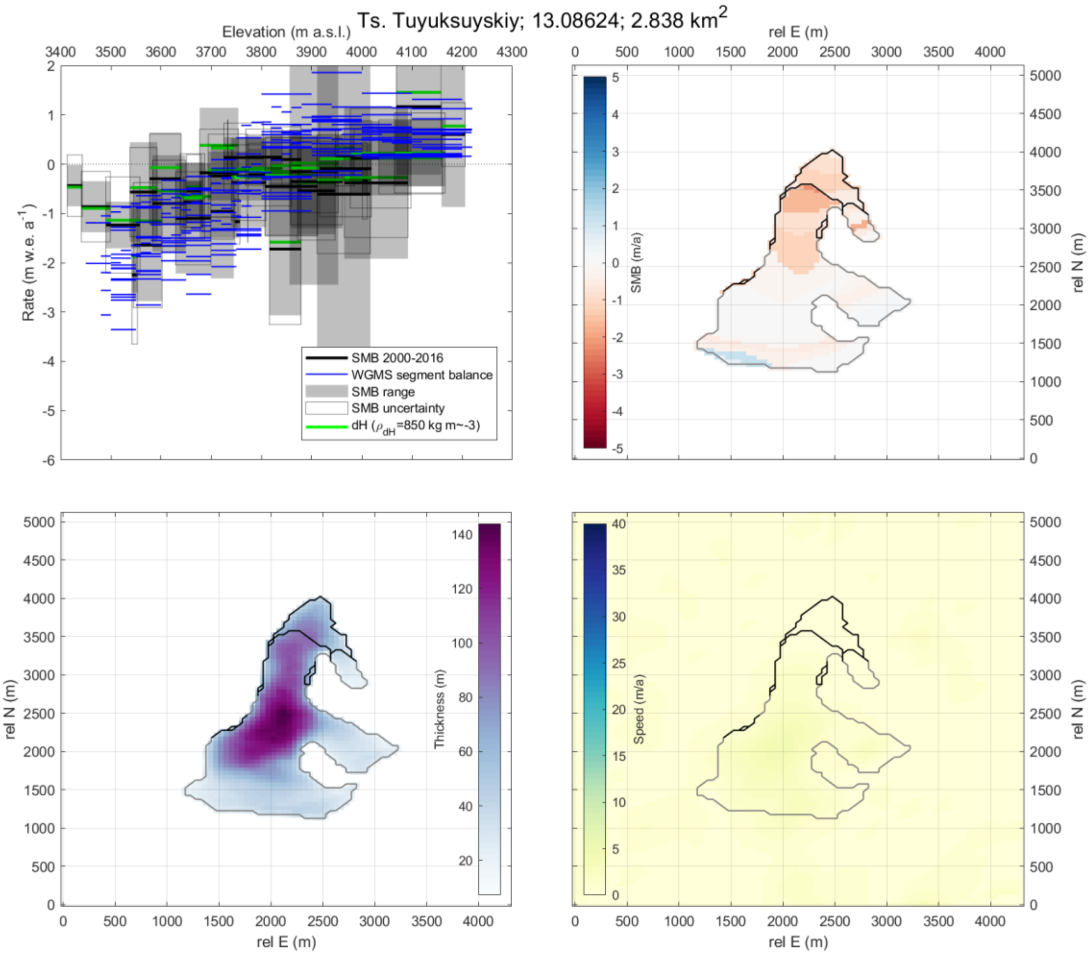


Supplementary Figure 30. Results for glacier 13.08624, as well as inputs and reference mass balance measurements. For this glacier, little ice motion is observable in the satellite observations (lower right), so our method models near-zero ice flux, and our results closely match the surface elevation changes but not the reference mass balance measurements (upper left).

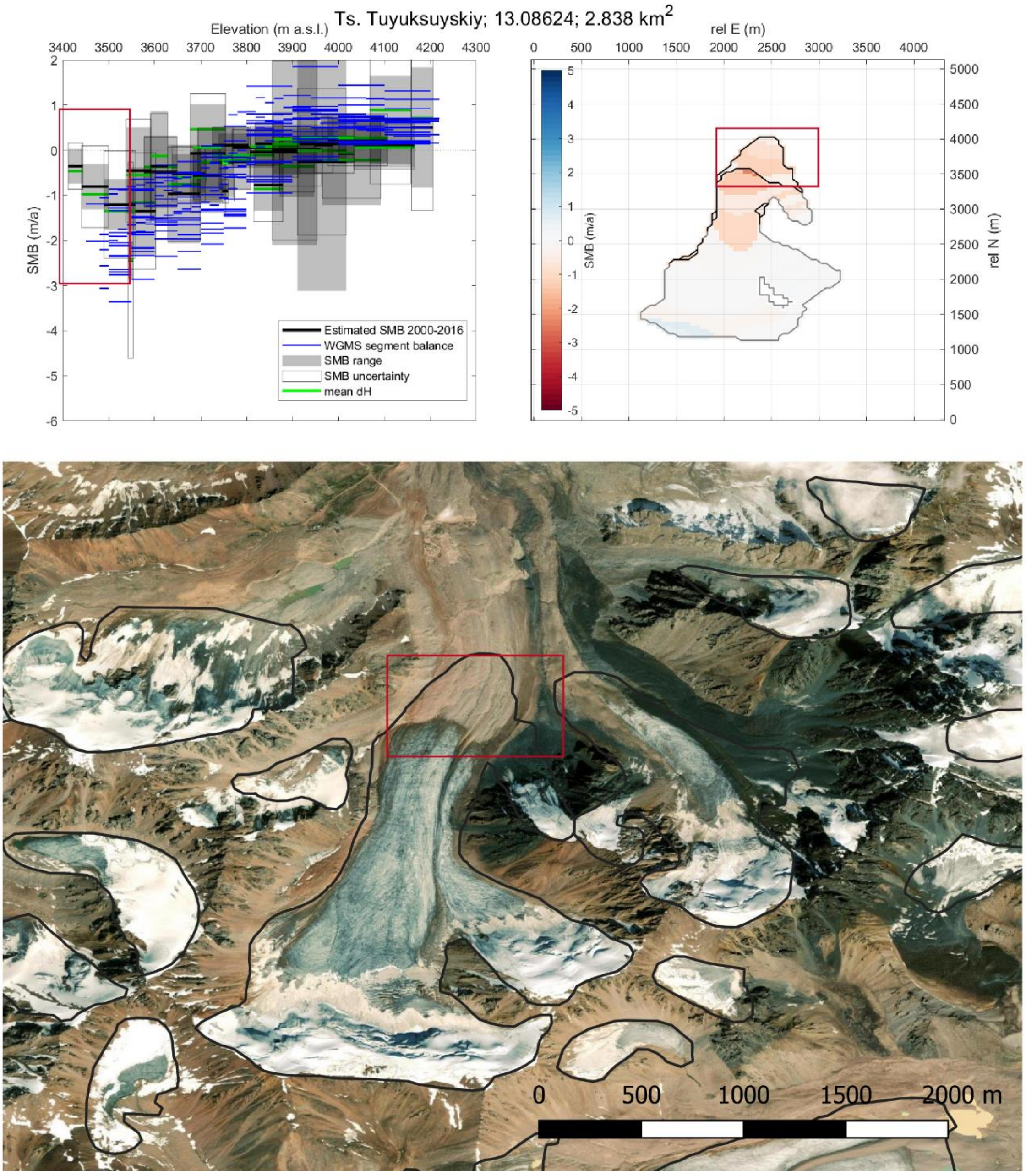


Supplementary Figure 31. Visual comparison of results for glacier 13.08624 to contemporary satellite image (courtesy ESRI). The mass balance at the RGI outline terminus, identified by the red box in all three panels, is negative for the 2000-2016 period. This area shows a reversed ablation gradient, but, as is apparent from the optical satellite image, this is not debris-covered ice (as erroneously indicated by the [70] dataset). The reversed ablation gradient is instead due to thinning and retreat at the terminus of this glacier.

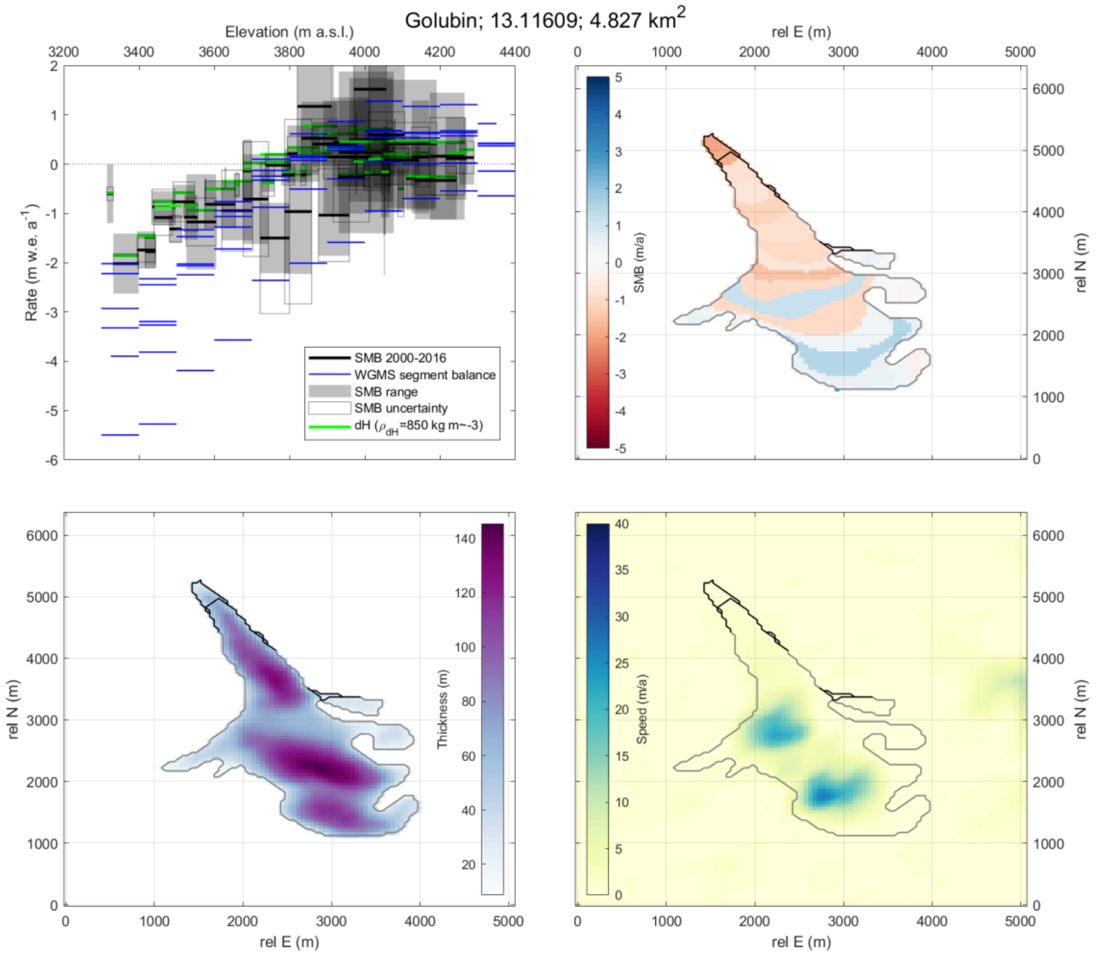


Supplementary Figure 32. Results for glacier 13.11609, as well as inputs and reference mass balance measurements. For this glacier, localized ice motion is observable in the satellite observations (lower right), but the velocity is not measurable in the lower glacier tongue. Consequently, our method models near-zero ice flux in this lower-elevation area, and our results closely match the surface elevation changes but not the reference mass balance measurements in this area (upper left). Where the velocity is observable, our results correspond with the reference measurements. The elevation band of apparent accumulation area is clearly attributable to the shallower ice thickness values in this region (whether correct or not) which partly correspond to increased surface velocity observations.

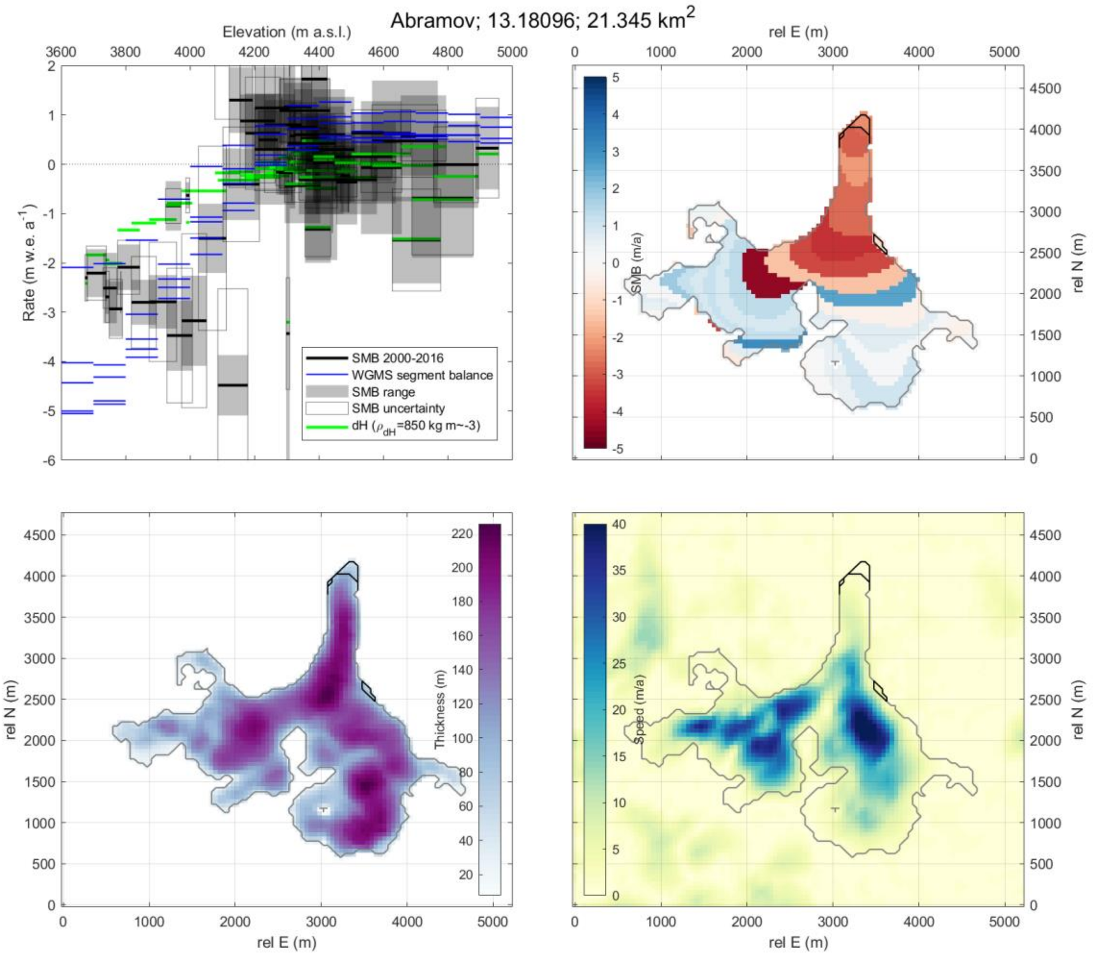


Supplementary Figure 33. Results for glacier 13.18906, as well as inputs and reference mass balance measurements. For this glacier, the velocity pattern leads to a coherent SMB pattern down-glacier, which follows reference measurements except at the terminus (where the observed velocity is low).

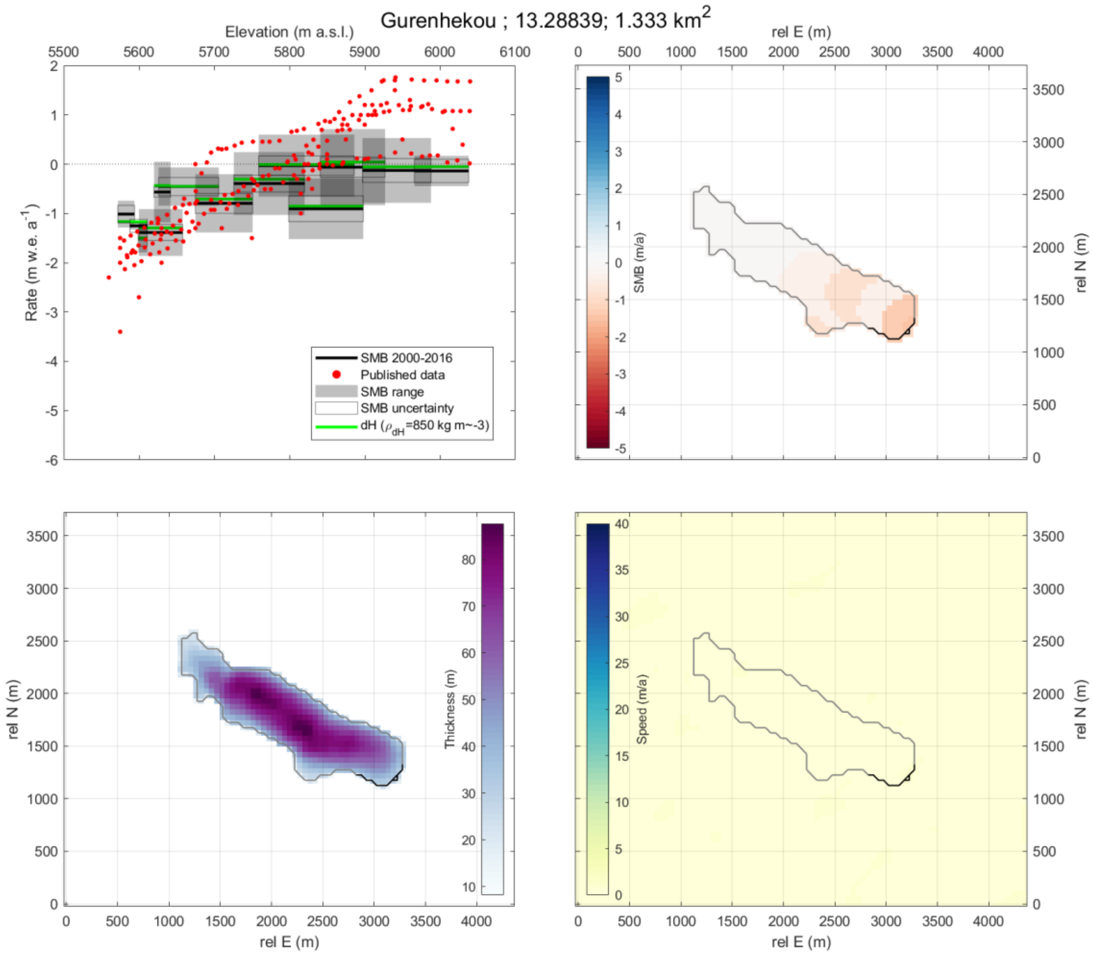


Supplementary Figure 34. Results for glacier 13.28839, as well as inputs and reference mass balance measurements. For this glacier, no velocity is measured, so the calculated SMB is equivalent to the measured *dH/dt*. This fits measurements reasonably well in the lower elevations, but the accumulation rate is not represented well.

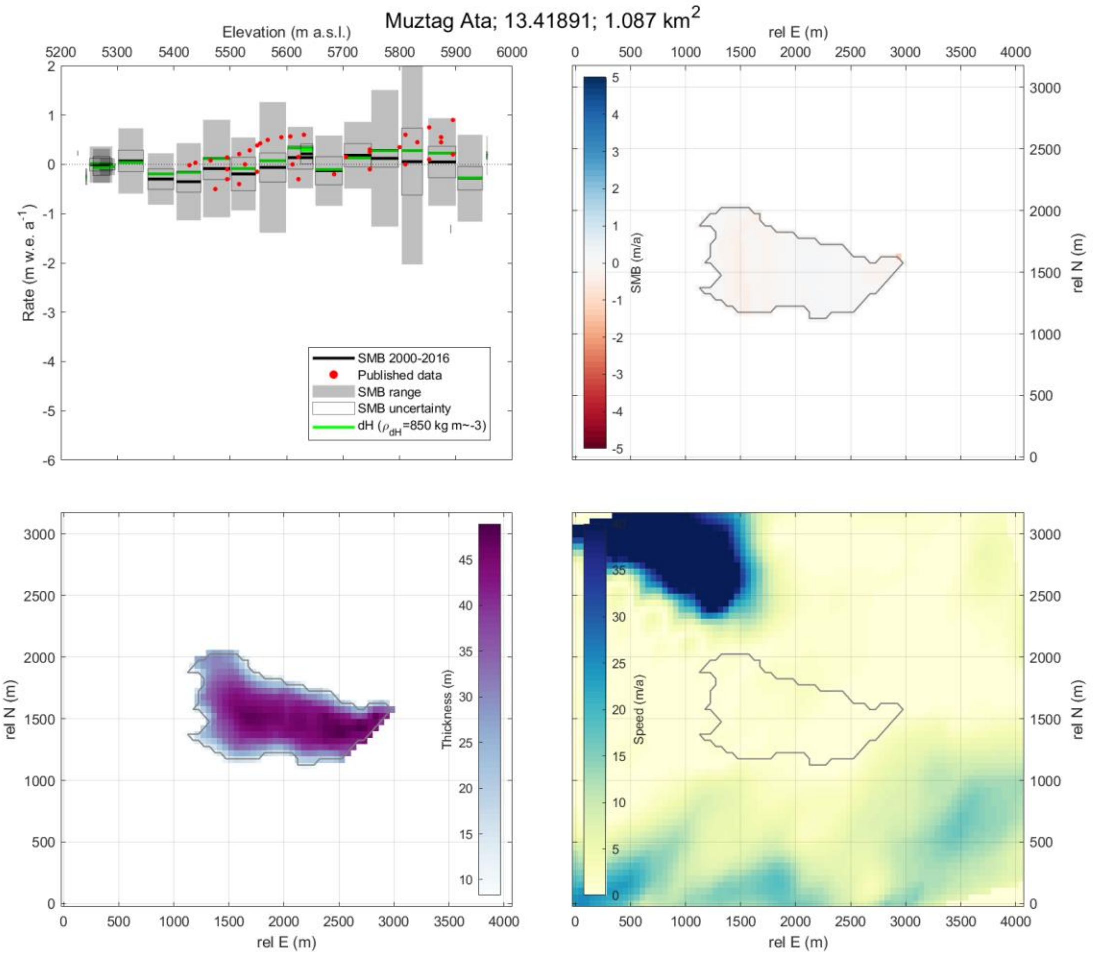


Supplementary Figure 35. Results for glacier 13.28839, as well as inputs and reference mass balance measurements. For this glacier, no velocity is measured, so the calculated SMB is equivalent to the measured *dH/dt*. This fits measurements reasonably well in the lower elevations, but the accumulation rate is not represented well.

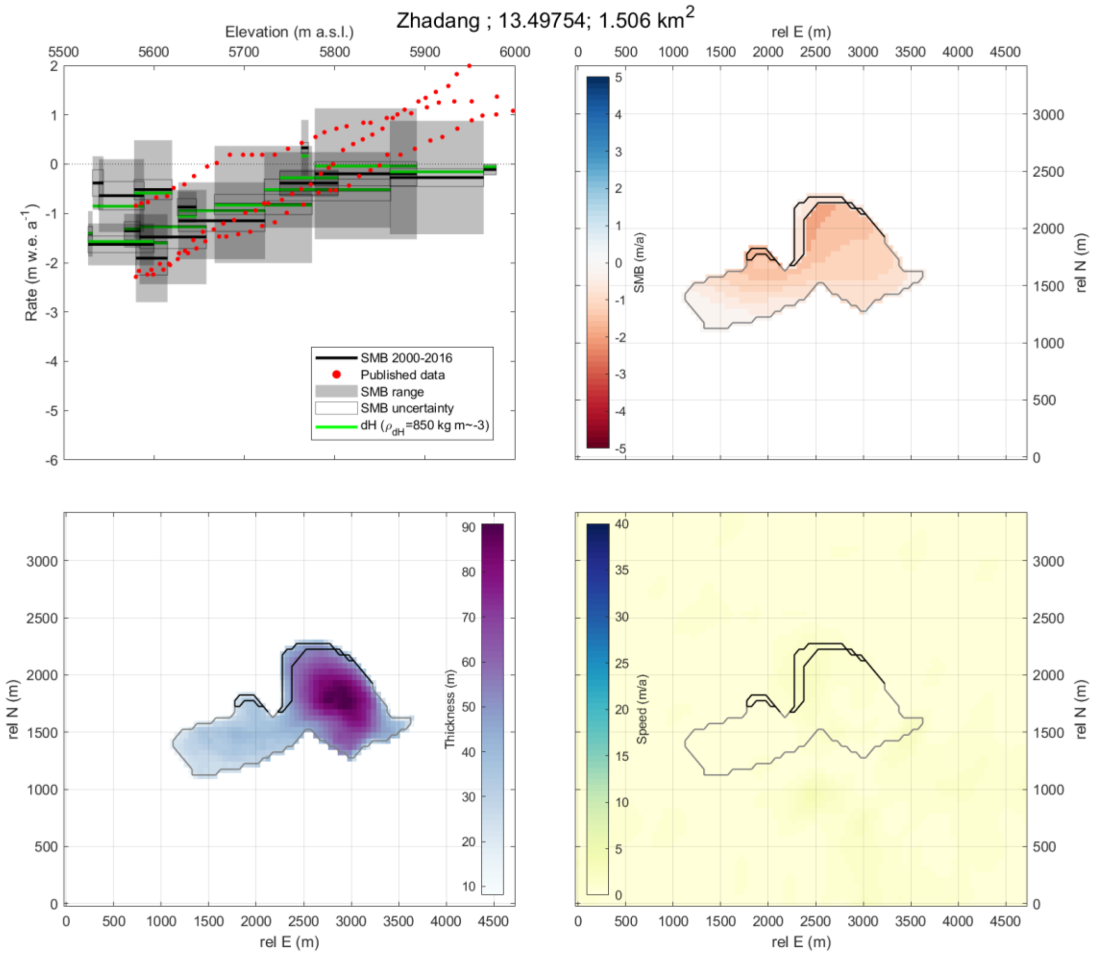


Supplementary Figure 36. Results for glacier 13.49754, as well as inputs and reference mass balance measurements. For this glacier, essentially no velocity is measured, so the calculated SMB is the measured *dH/dt*. The resulting pattern fits the magnitude of observations but poorly represents the mass balance gradient.

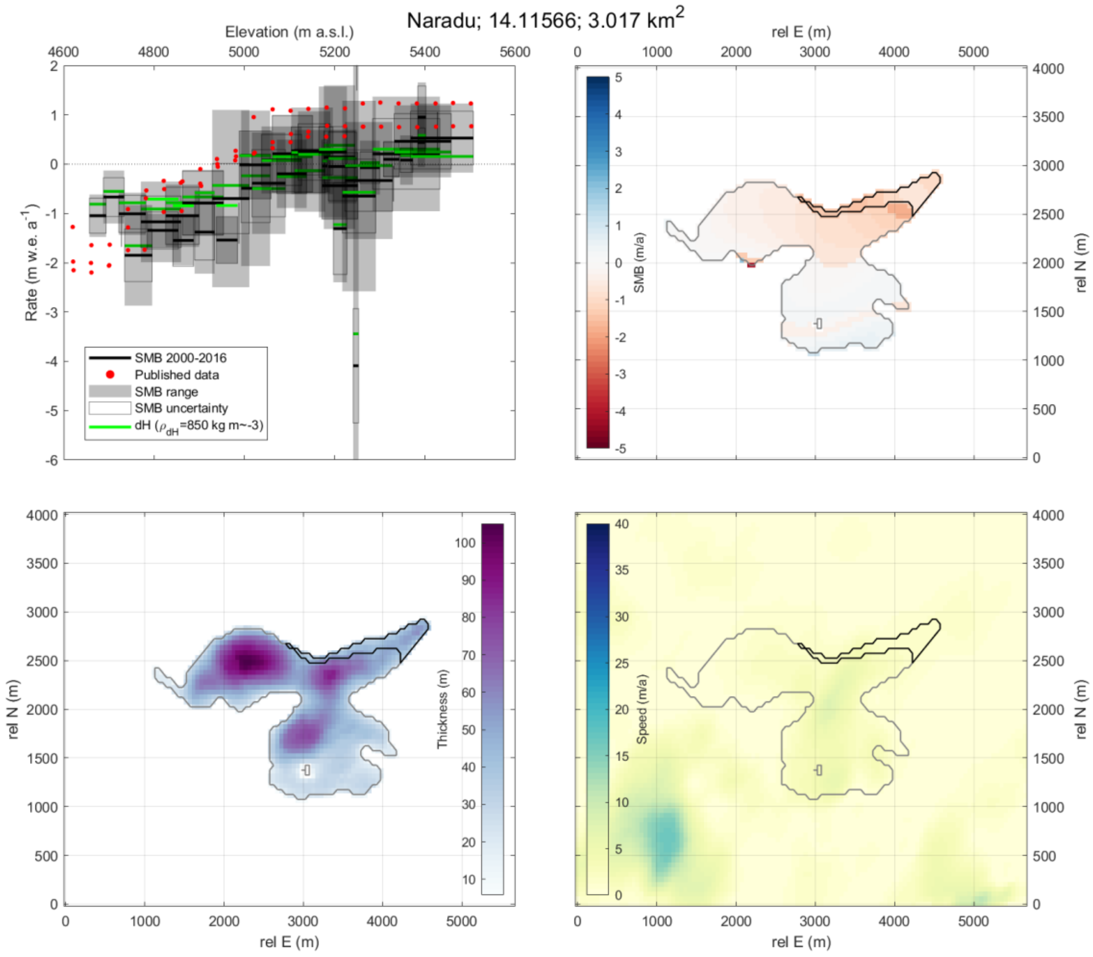


Supplementary Figure 37. Results for glacier 14.11566, as well as inputs and reference mass balance measurements. For this glacier, gentle observed velocity leads to a clear SMB gradient that corresponds closely to the reference measurements, although with a slight negative bias. We note that the *dH/dt* patterns are also biased negative to reference measurements, and that the reference measurements are from 2000-2002, suggesting a shift in mass balance over the study period.

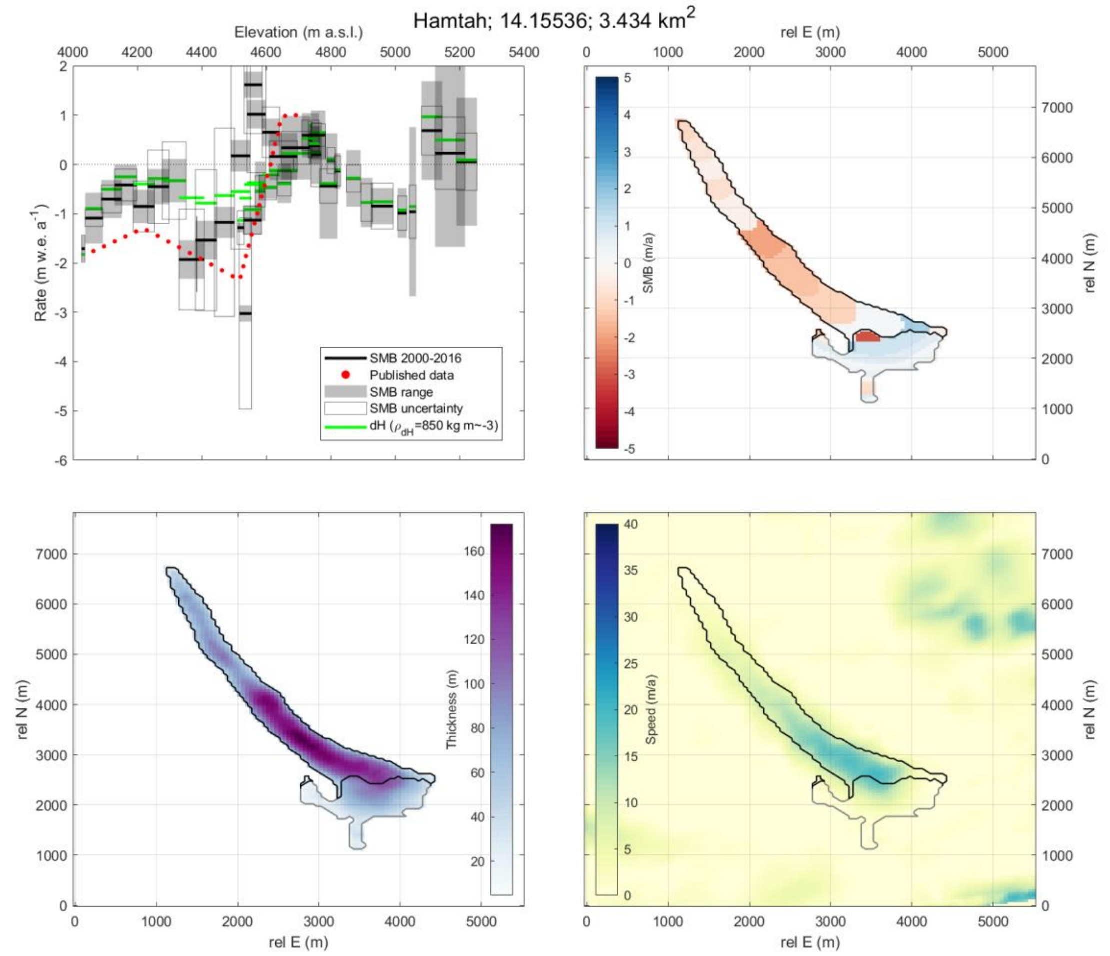


Supplementary Figure 38. Results for glacier 14.15536, as well as inputs and reference mass balance measurements. For this glacier, the velocity and thickness patterns produce a variable SMB pattern over the lower glacier, which, although strange, corresponds well with the reference measurements in magnitude and general pattern. We note that the reference measurements for this glacier are an idealised profile.

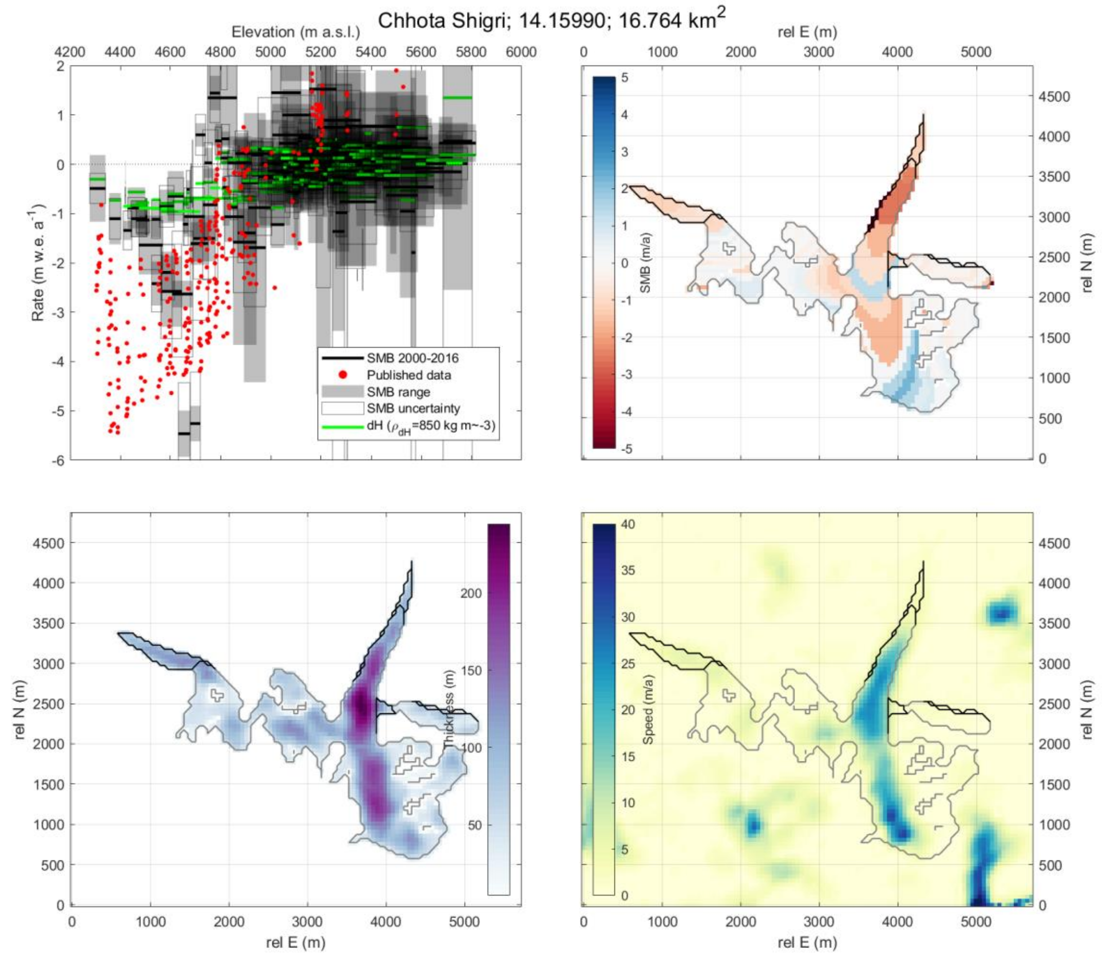


Supplementary Figure 39. Results for glacier 14.15990, as well as inputs and reference mass balance measurements. We note that this glacier (from the RGI) actually contains two distinct ice bodies, and that measurements only correspond to the eastern (right) glacier. For this glacier, the observed velocity leads to a clear SMB gradient that corresponds closely to the reference measurements for much of the glacier. Two problems are evident: the apparent gap in velocity between the accumulation and ablation areas leads to alternating emergence and submergence. Also, the lack of observable velocity near the terminus leads to a reverse gradient not present in the reference measurements.

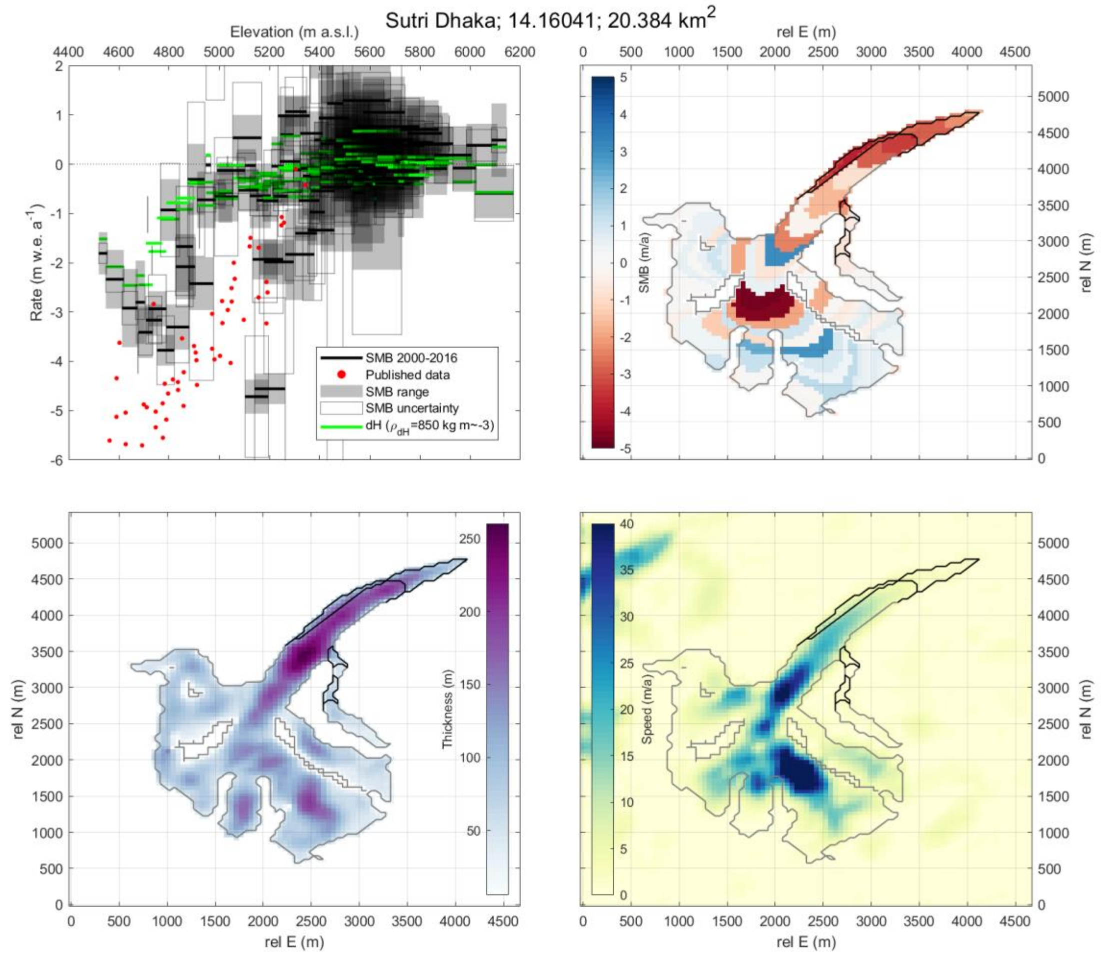


Supplementary Figure 40. Results for glacier 14.16041, as well as inputs and reference mass balance measurements. For this glacier, the velocity pattern is not smoothly-varying, which leads to SMB variations in our results that do not correspond well with reference measurements.

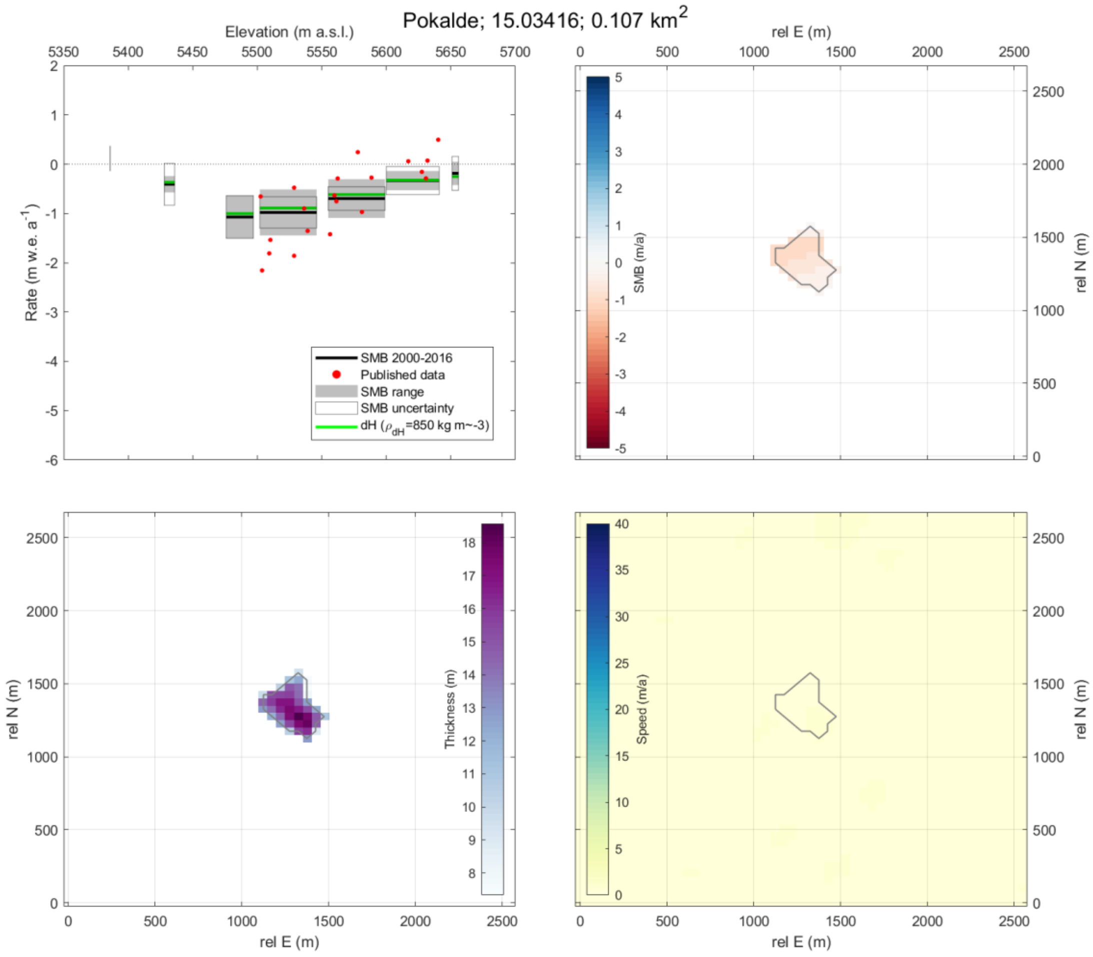


Supplementary Figure 41. Results for glacier 15.03416, as well as inputs and reference mass balance measurements. Ice motion is not observable for this very small glacier, but the *dH/dt* measurements closely correspond to reference measurements.

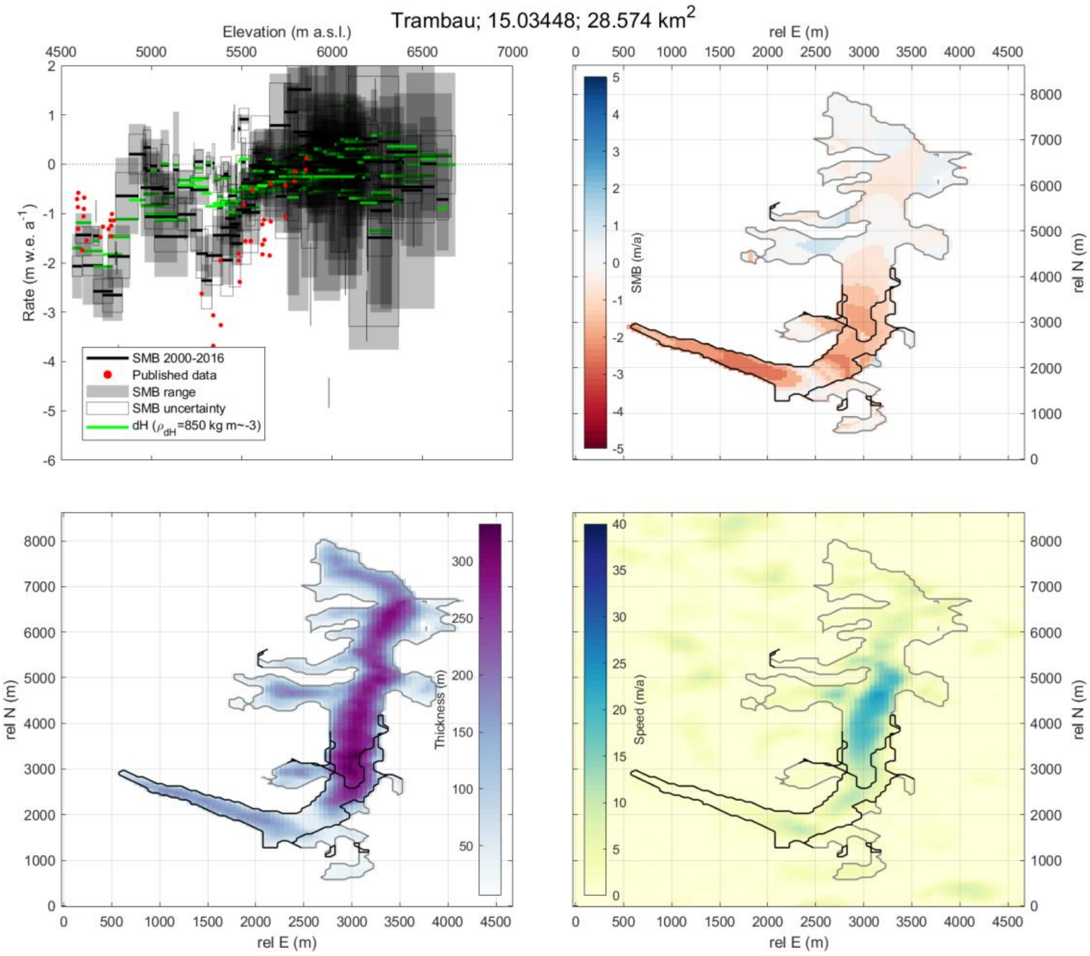


Supplementary Figure 42. Results for glacier 15.03448, as well as inputs and reference mass balance measurements. For this large glacier, the clear velocity pattern leads to a pattern of SMB closely corresponding with reference measurements in both the middle and lower sections of the glacier. We note that measurements for this glacier correspond to the very end of our study period.

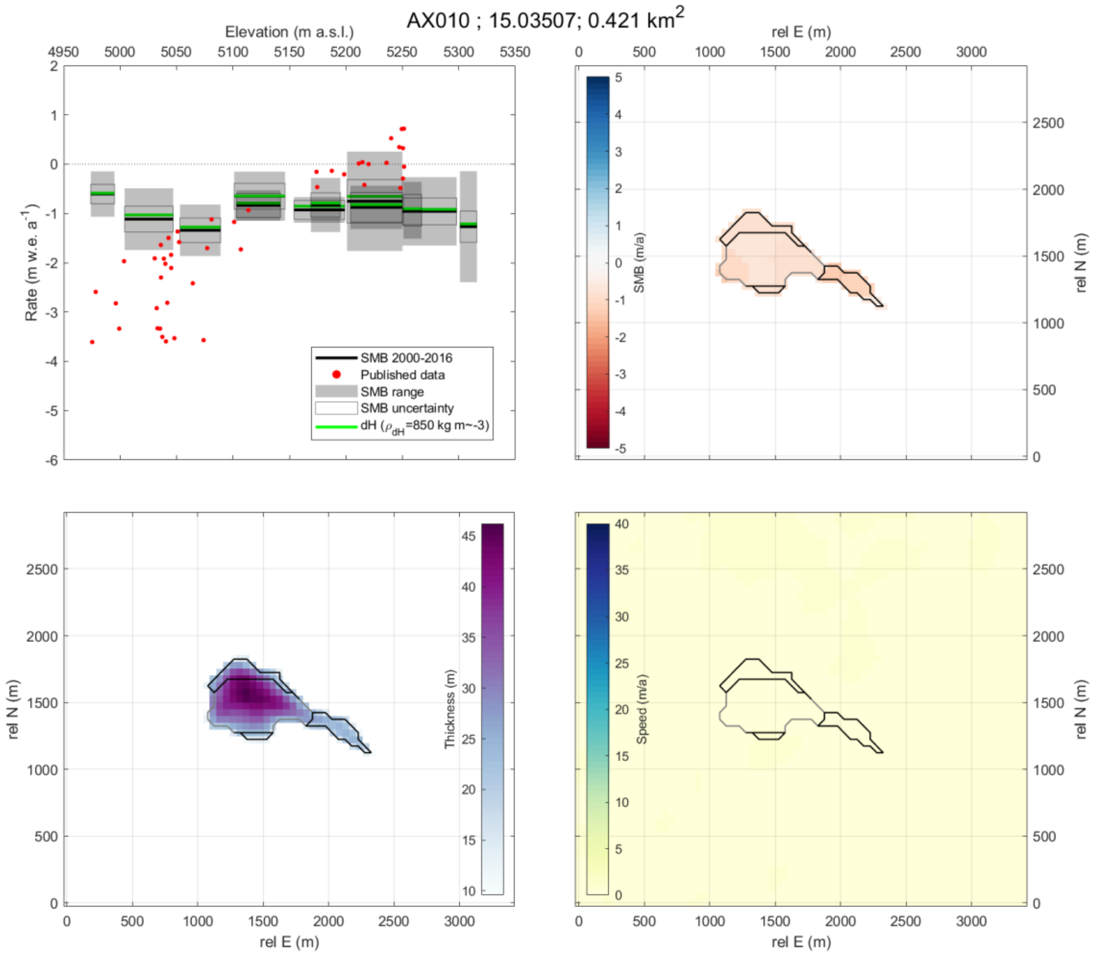


Supplementary Figure 43. Results for glacier 15.03507, as well as inputs and reference mass balance measurements. For this very small glacier, no velocity is measured and the thinning pattern does not correspond to reference measurements, which slightly preceded our study period.

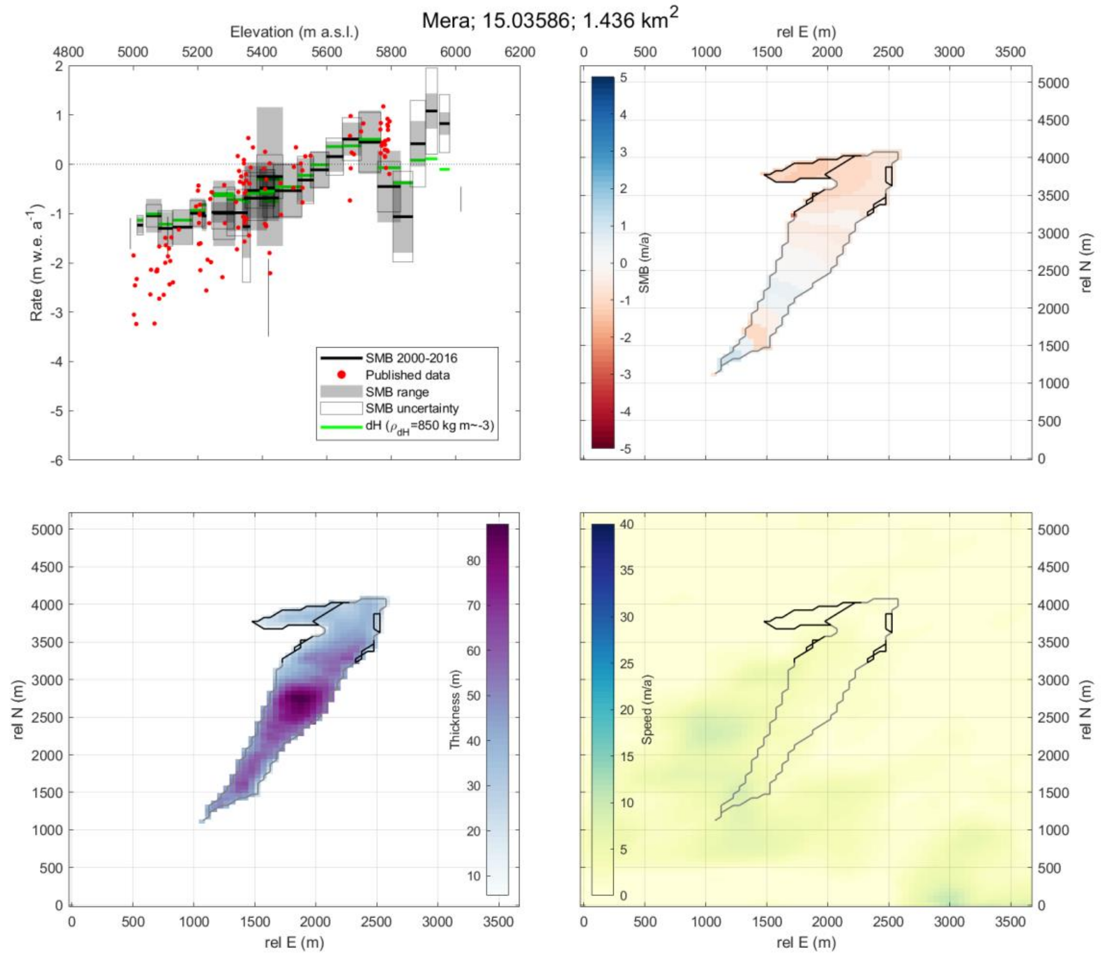


Supplementary Figure 44. Results for glacier 15.03586, as well as inputs and reference mass balance measurements. For this large glacier, only a slight pattern of velocity is evident, but the SMB pattern closely corresponds with reference measurements except at the glacier terminus

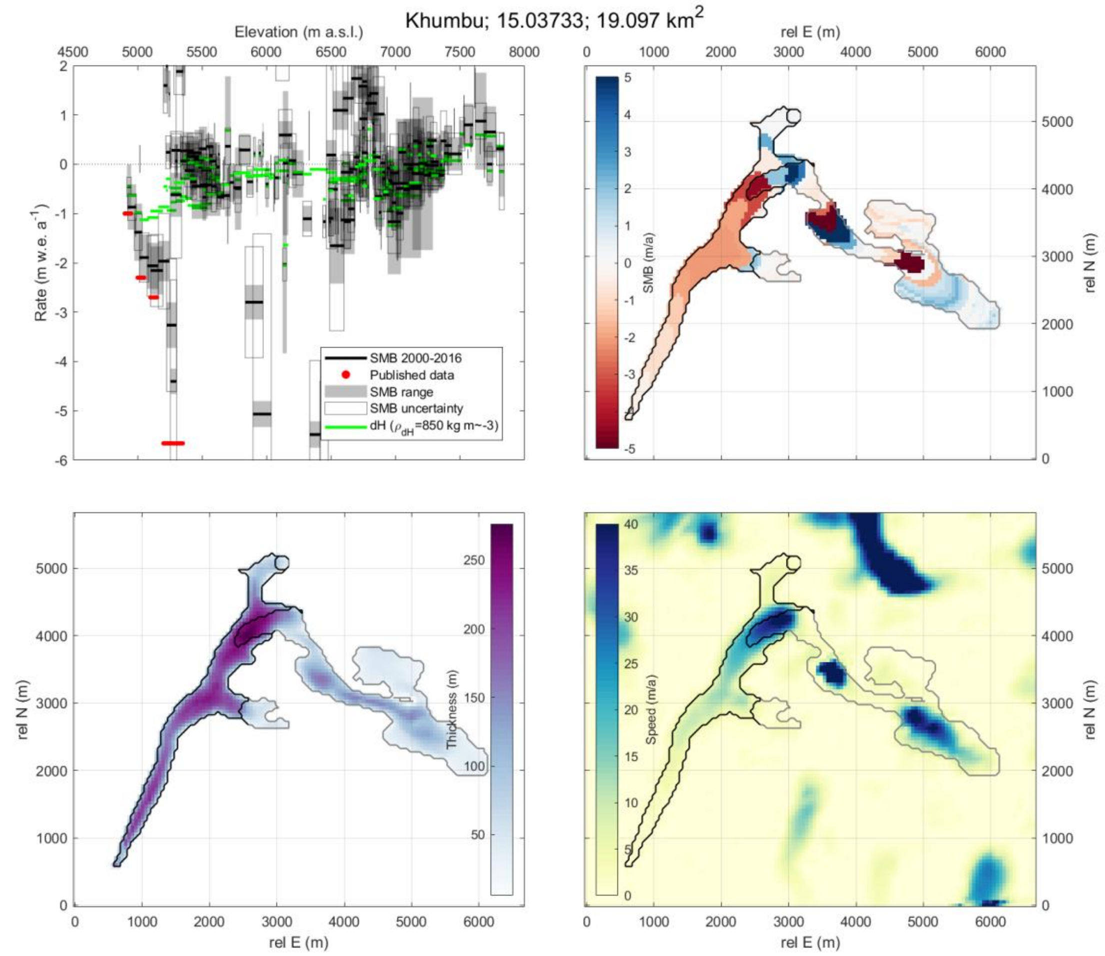


Supplementary Figure 45. Results for glacier 15.03733, as well as inputs and reference mass balance measurements. For this large glacier, the SMB closely corresponds with reference measurements for the lower tongue. However, the velocity data are highly discontinuous, which leads to unrealistic undulations in SMB above the reference measurements. We note that the reference measurements for this glacier used a distinct implementation of the continuity approach with entirely independent data.

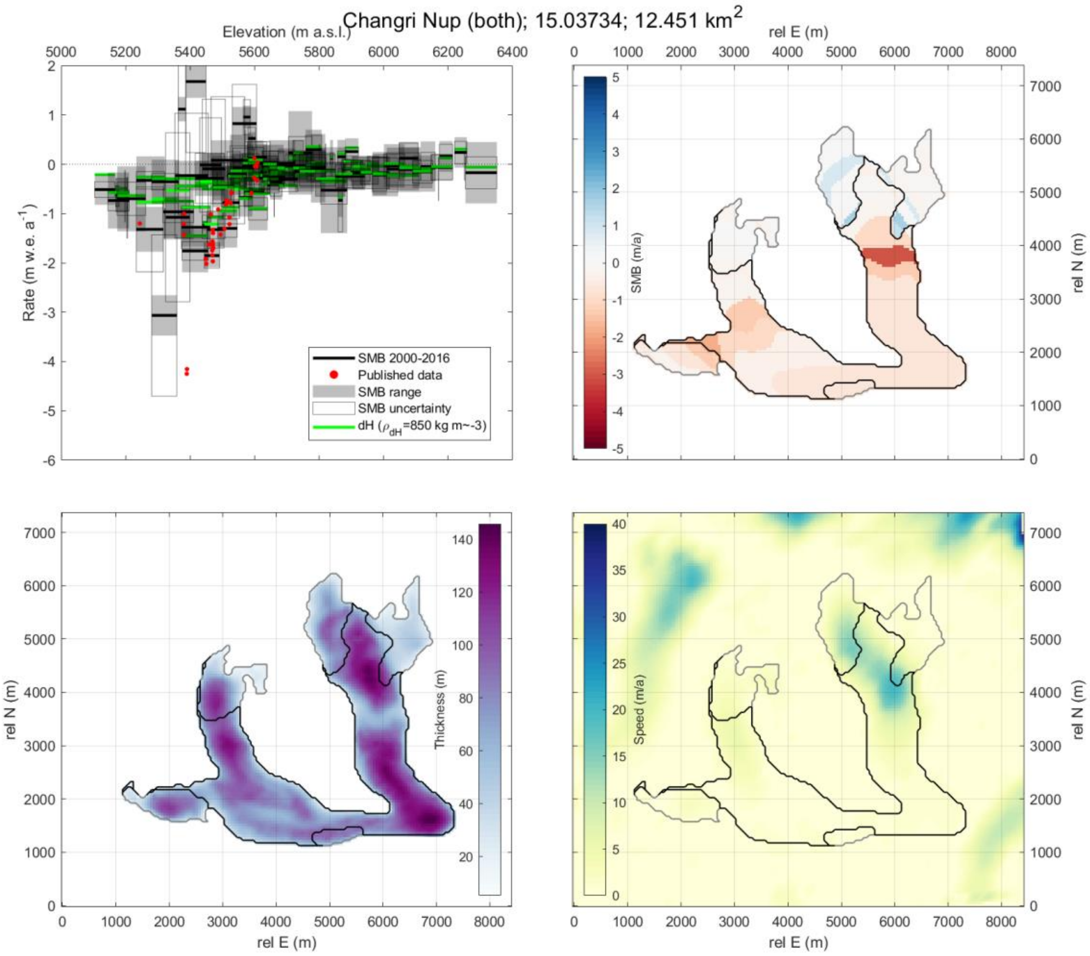


Supplementary Figure 46. Results for glacier 15.03734, as well as inputs and reference mass balance measurements. We note that this glacier (from the RGI) actually consists of three flow units that are no longer connected and are normally considered separately. Nonetheless, our SMB results largely correspond to measurements taken on the left two glaciers, and reproduce both the magnitude and gradient of mass balance.

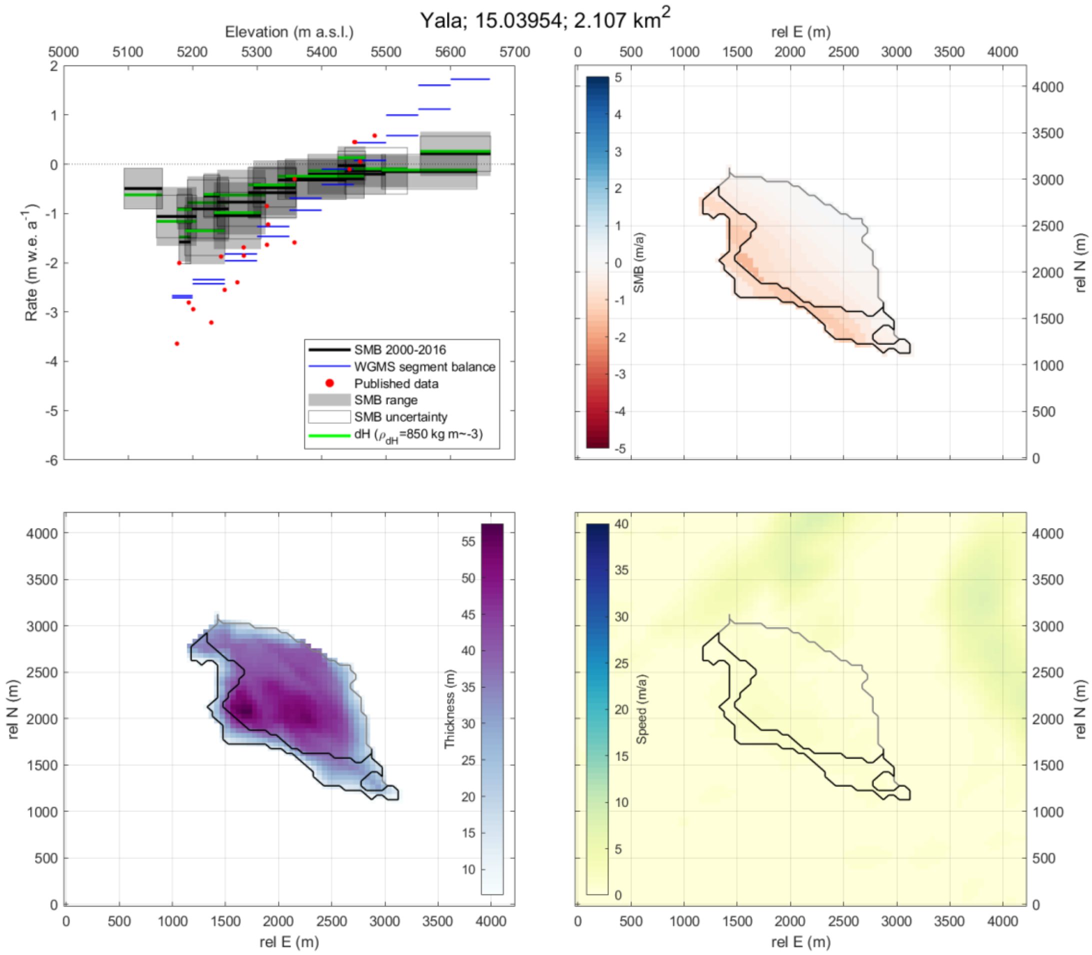


Supplementary Figure 47. Results for glacier 15.03954, as well as inputs and reference mass balance measurements. For this large glacier, no velocity is measured and although the SMB pattern marginally corresponds to the magnitude of reference measurements, the mass balance gradient is poorly represented.

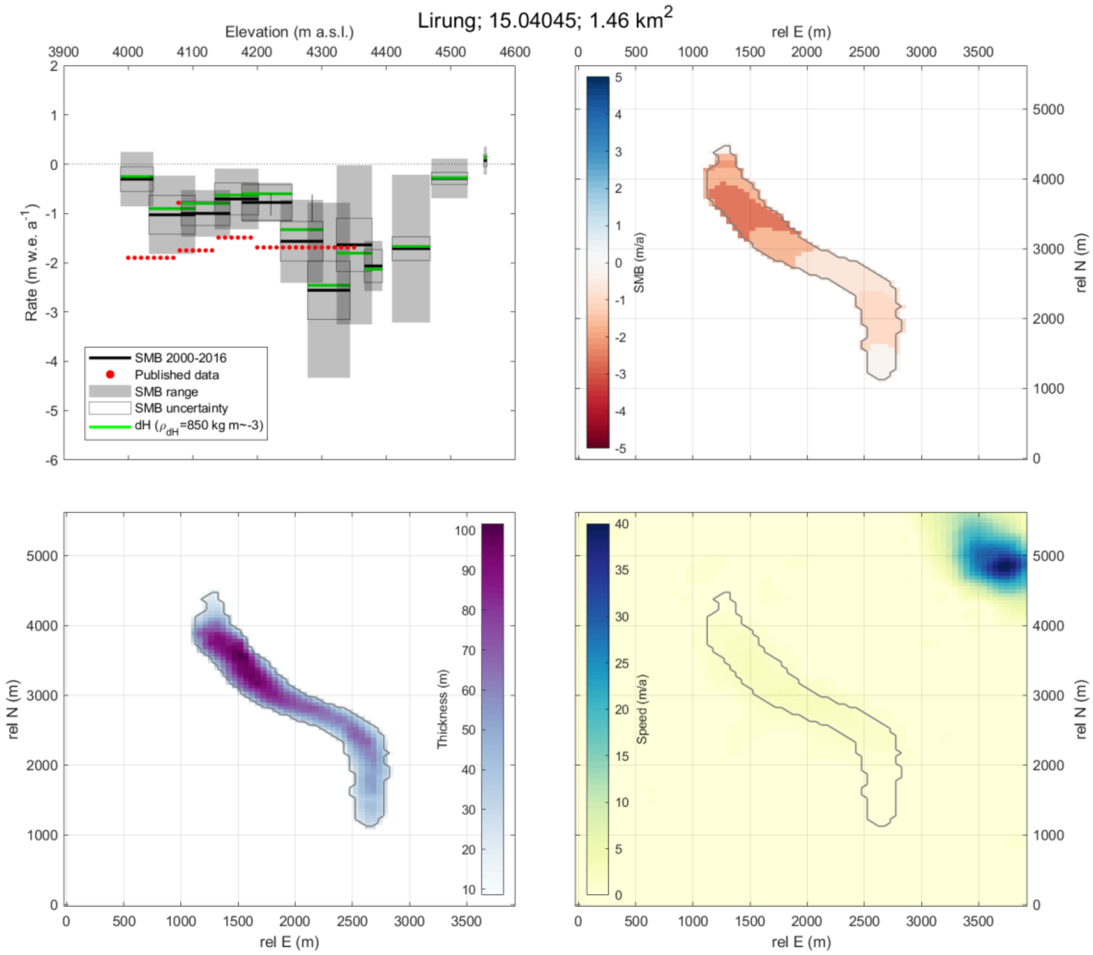


Supplementary Figure 48. Results for glacier 15.04045, as well as inputs and reference mass balance measurements. For this large glacier, only slight velocity is measured, but the results reproduce the pattern observed in reference measurements for the lower tongue for the early portion of our study period, as well as the magnitude of more recent mass balance indicated by a single stake. We note that most of the reference measurements for this glacier used a distinct implementation of the continuity approach with entirely independent data.

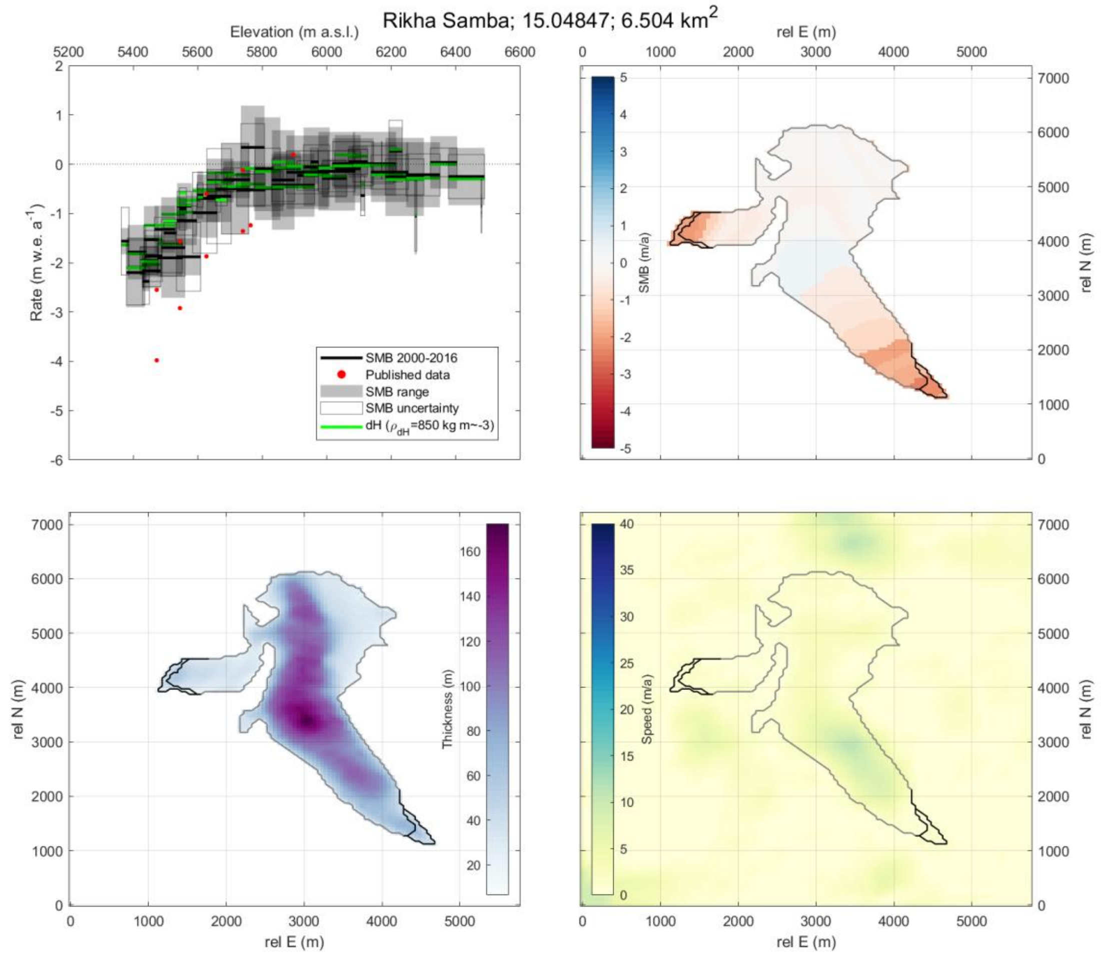


Supplementary Figure 49. Results for glacier 15.04847, as well as inputs and reference mass balance measurements. For this large glacier, only moderate velocity is observed, and the results approximate but slightly underestimate the pattern observed in reference measurements.

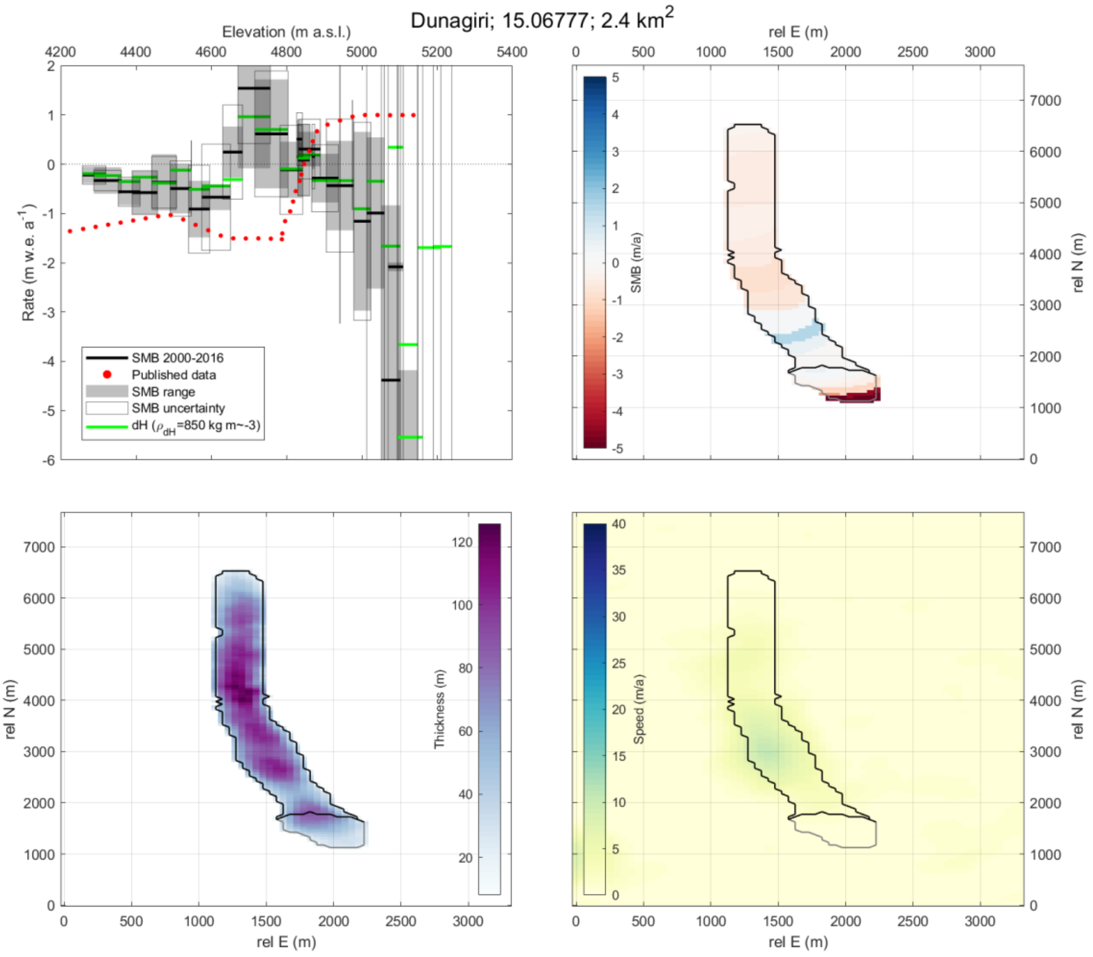


Supplementary Figure 50. Results for glacier 15.06777, as well as inputs and reference mass balance measurements. For this glacier, very little velocity is observed, are the results do not correspond to reference measurements. We note that the reference measurements are an idealised profile.

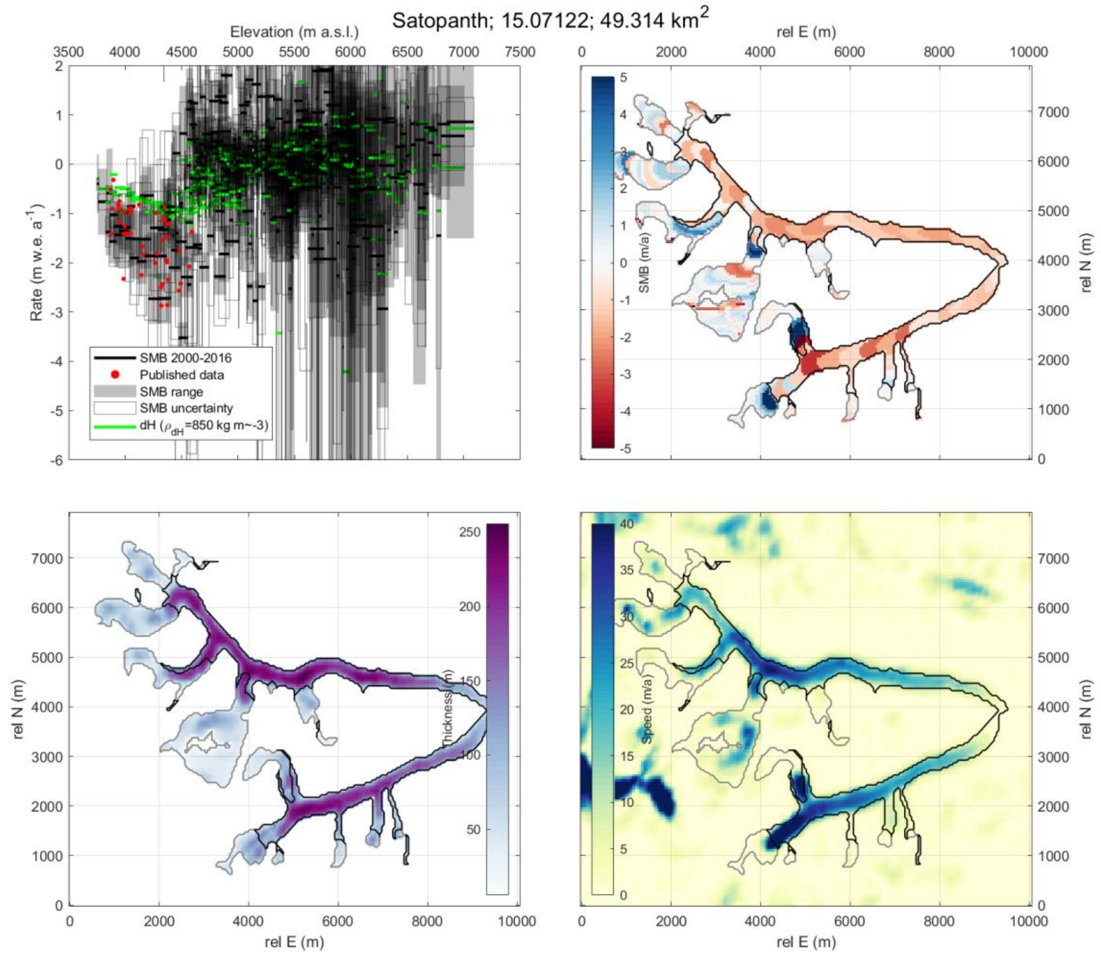


Supplementary Figure 51. Results for glacier 15.07122, as well as inputs and reference mass balance measurements. For this glacier, our results closely correspond to the measured mass balance. We note that this glacier outline corresponds to two individual glaciers, and measurements were only taken from the southern (lower) glacier.

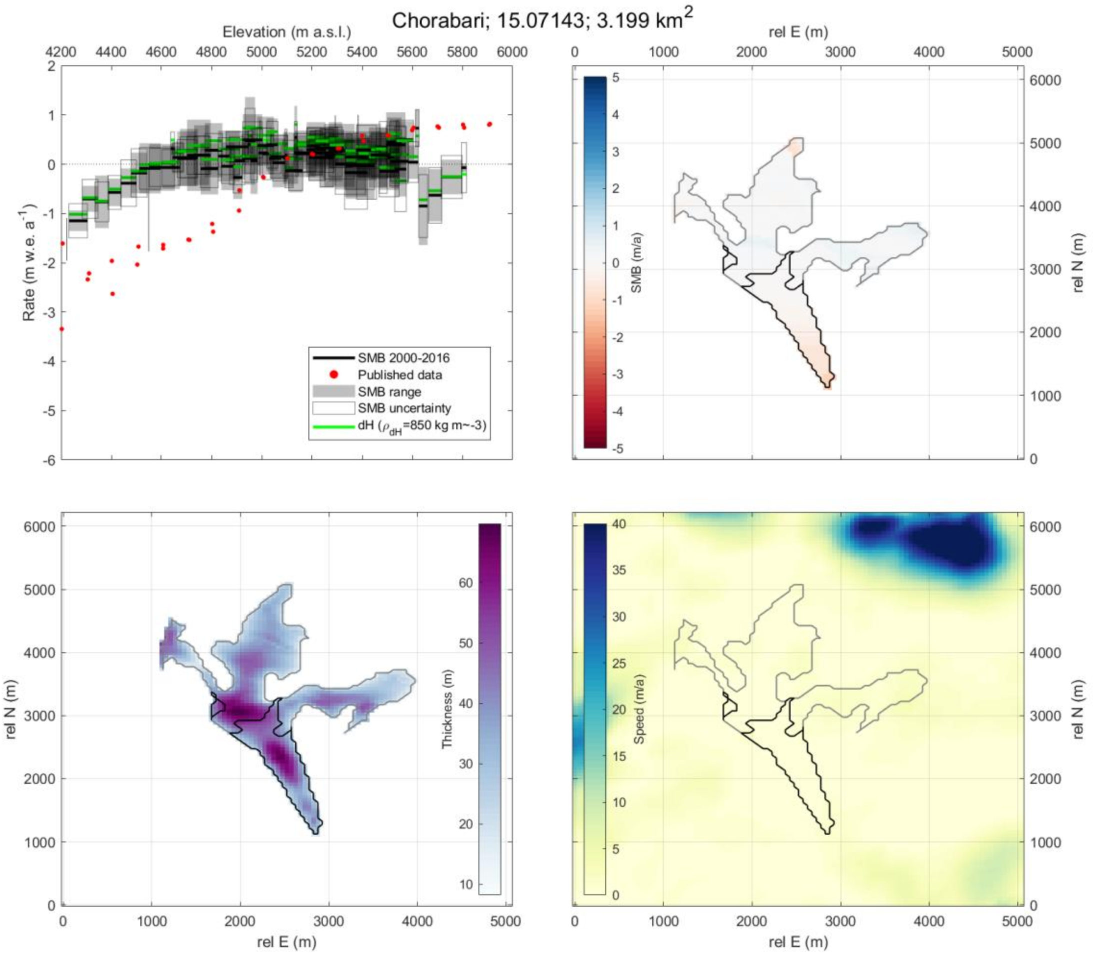


Supplementary Figure 52. Results for glacier 15.07143, as well as inputs and reference mass balance measurements. For this glacier, very little velocity is observed and our results do not correspond to the measured mass balance.

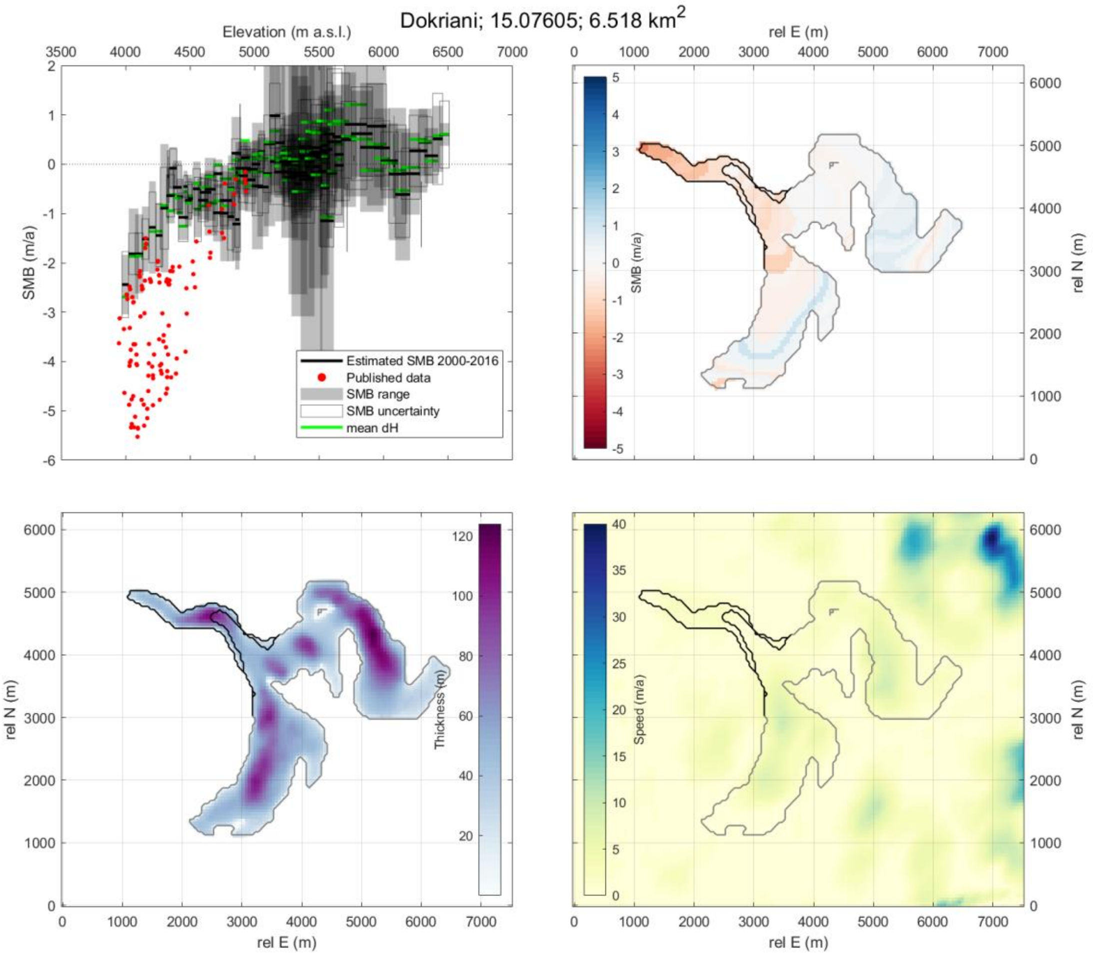


Supplementary Figure 53. Results for glacier 15.07605, as well as inputs and reference mass balance measurements. For this glacier, very little velocity is observed over the lower tongue, where most reference measurements were taken and our results in this area do not correspond to the measured mass balance. However, our results in the middle section of the glacier correspond closely to measurements in terms of magnitude and gradient.

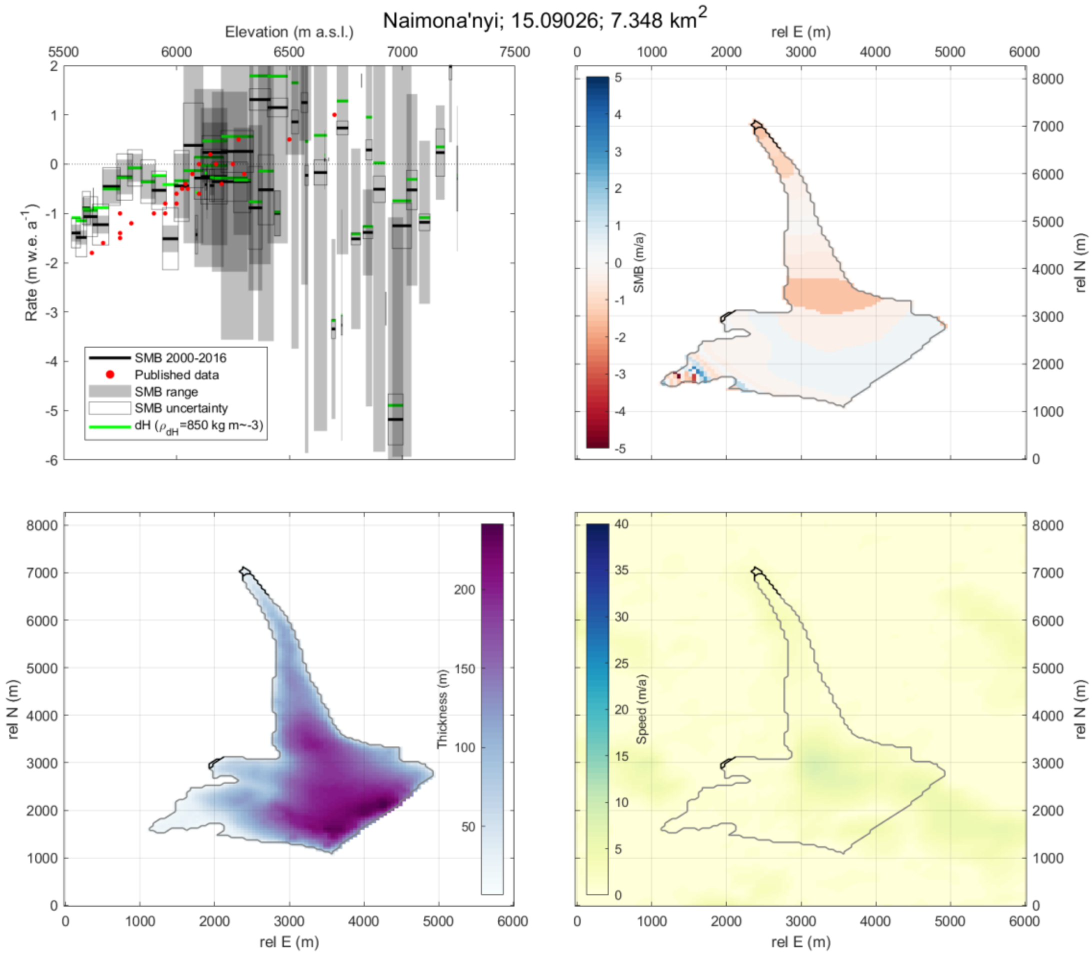


Supplementary Figure 54. Results for glacier 15.09026, as well as inputs and reference mass balance measurements. For this glacier, very little velocity is observed over the lower tongue, where most reference measurements were taken and our results in this area thus do not correspond to the measured mass balance. In addition, vrey small elevation bands at high elevation show unrealistically erratic SMB patterns.

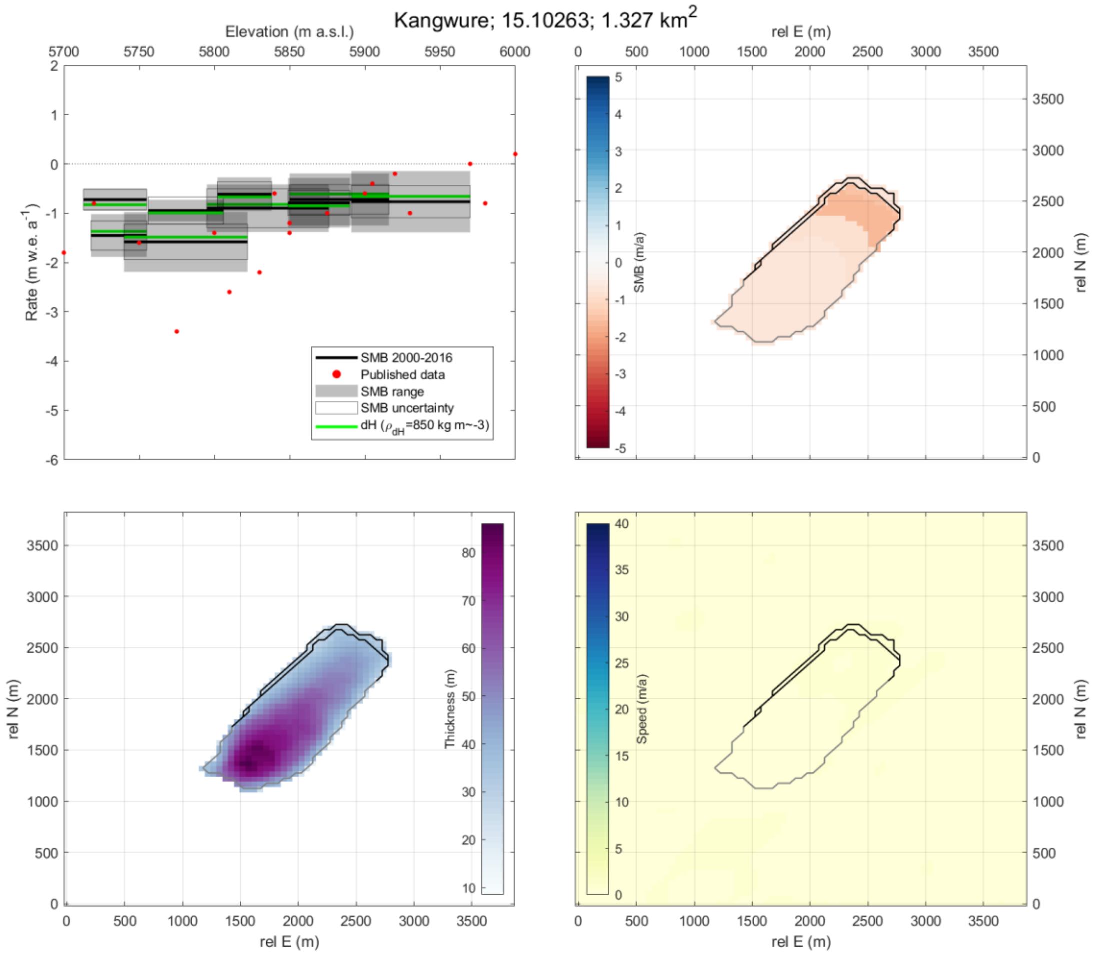


Supplementary Figure 55. Results for glacier 15.10263, as well as inputs and reference mass balance measurements. For this glacier, no velocity is measured and our results overlap with but underestimate the measured mass balance.

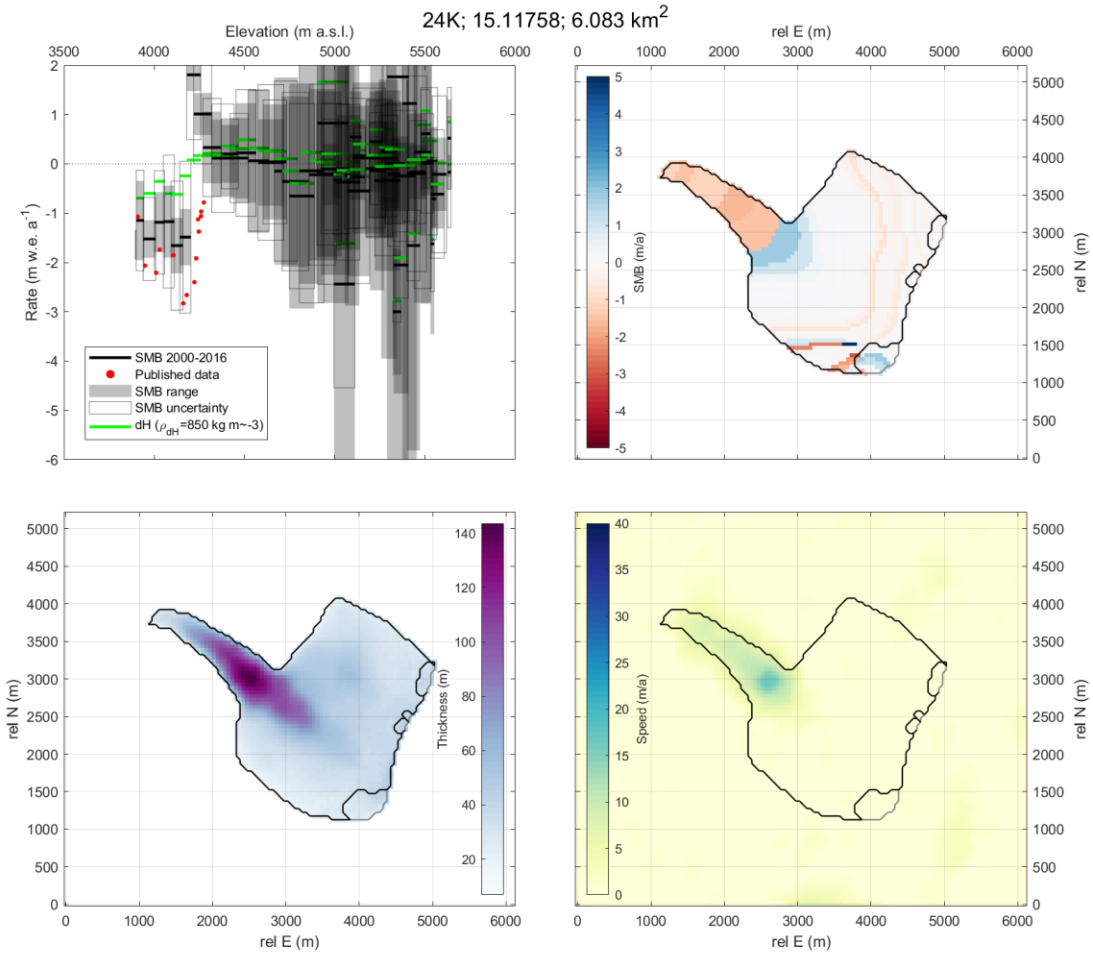


Supplementary Figure 56. Results for glacier 15.11758, as well as inputs and reference mass balance measurements. For this glacier, our results correspond to the measured mass balance in the lower tongue, with a slight bias. We note that for this glacier the outline is unrealistically simple.

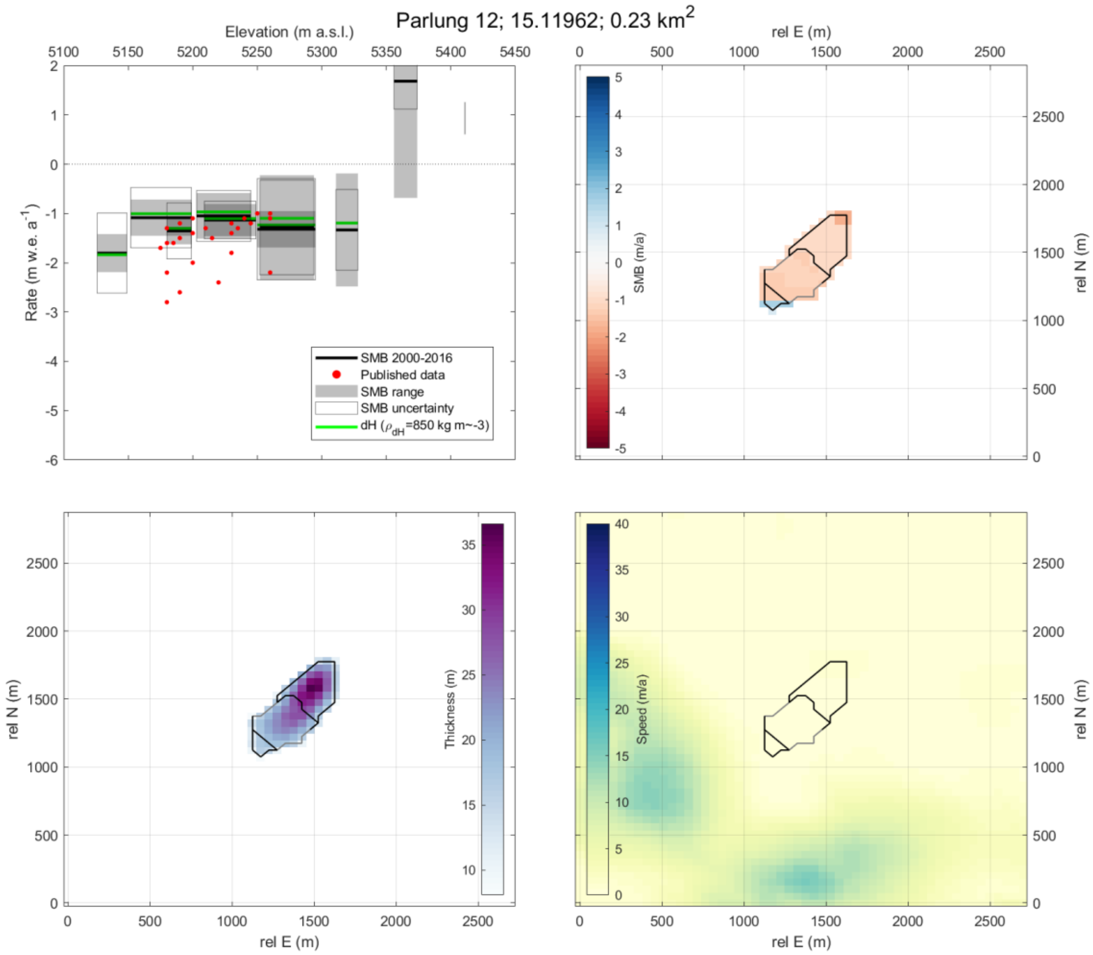


Supplementary Figure 57. Results for glacier 15.11962, as well as inputs and reference mass balance measurements. For this very small glacier, little velocity is observed but our results correspond to the measured mass balance in the lower tongue.

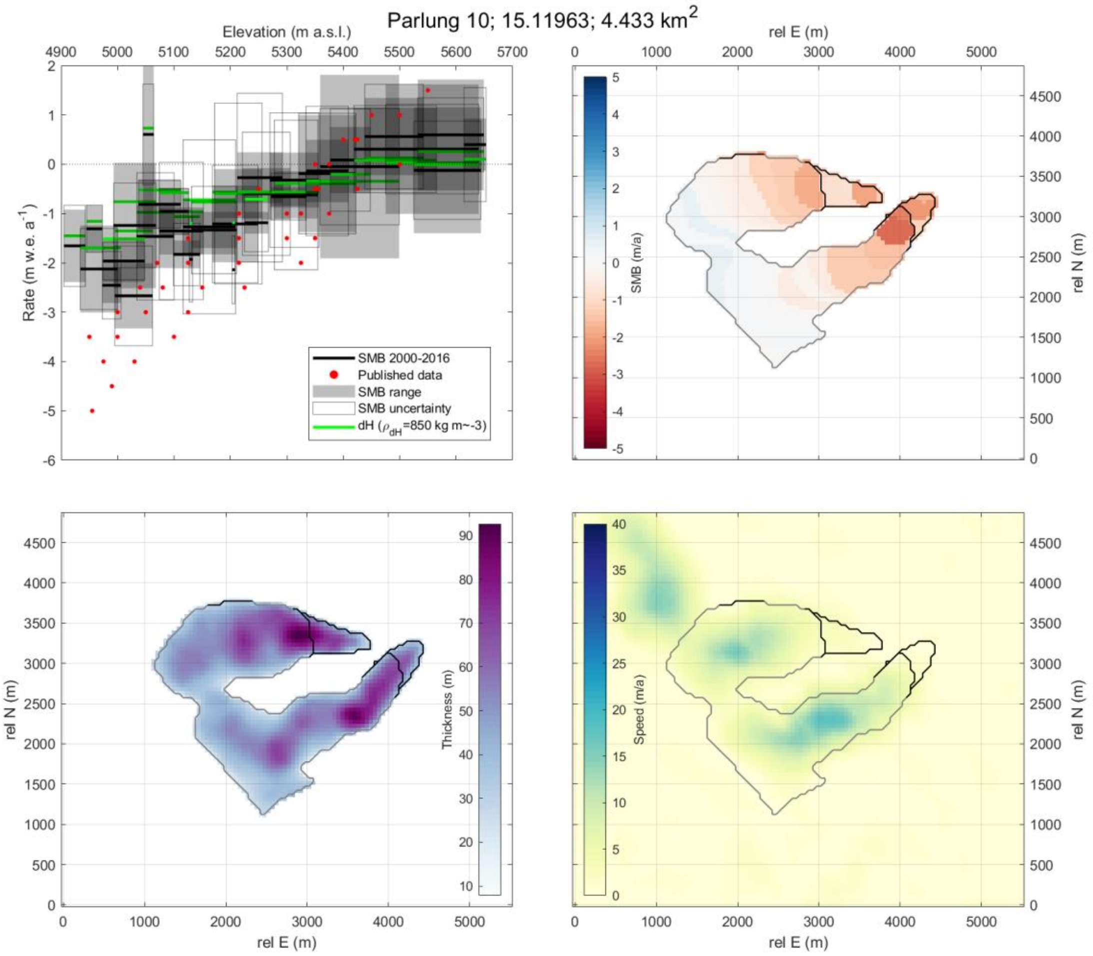


Supplementary Figure 58. Results for glacier 15.11963, as well as inputs and reference mass balance measurements. For this glacier, the SMB correspond reasonably well with observations, although the mass balance gradient is shallower than observed.

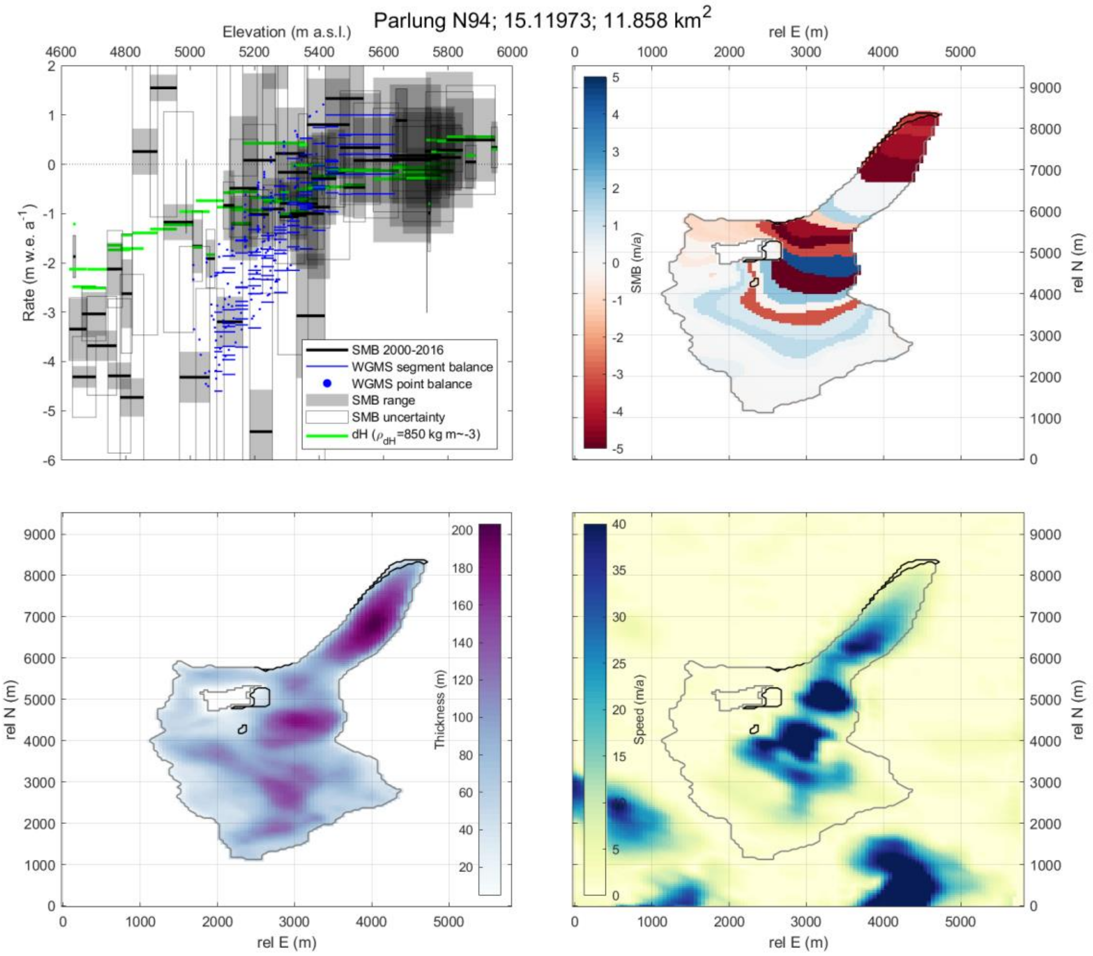


Supplementary Figure 59. Results for glacier 15.11973, as well as inputs and reference mass balance measurements. For this glacier, the velocity and thickness patterns vary dramatically, leading to unrealistic SMB oscillations that do not correspond to reference measurements.

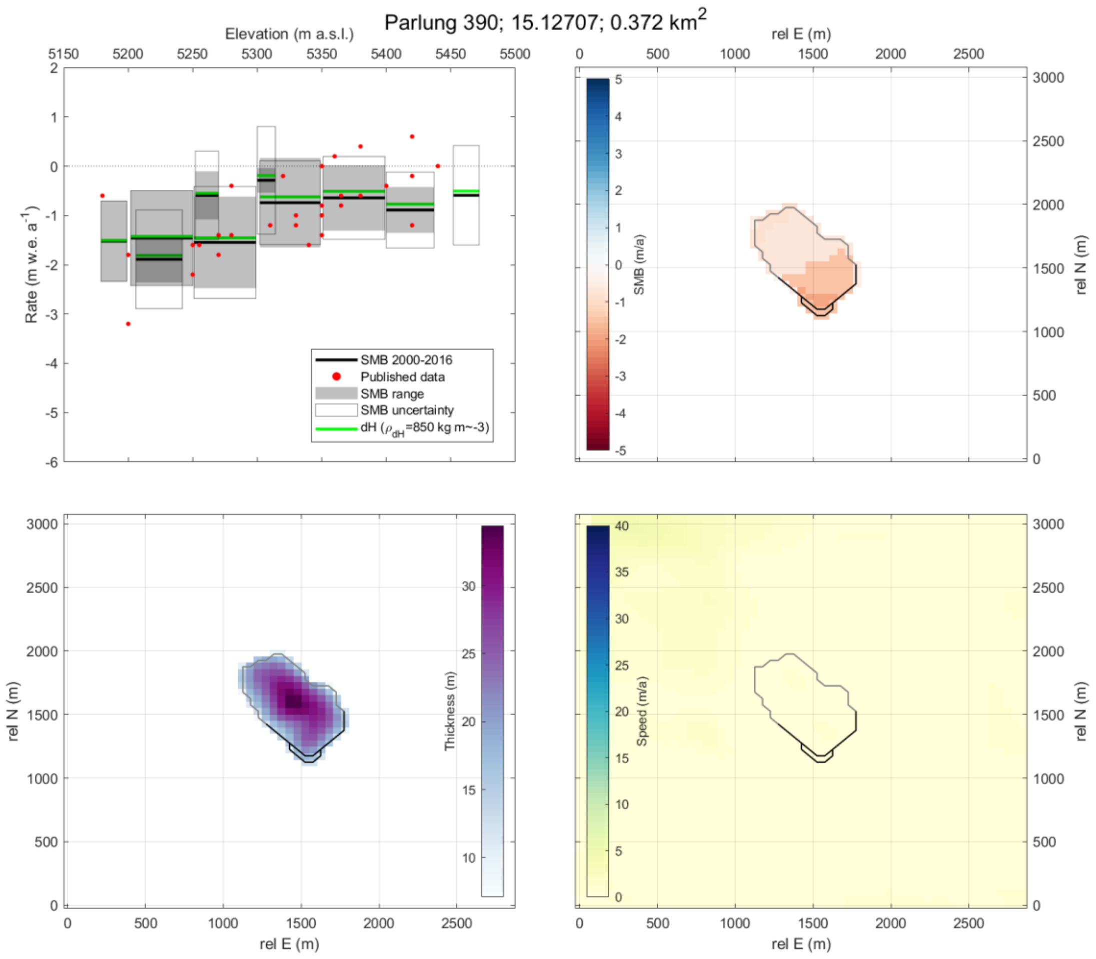


Supplementary Figure 60. Results for glacier 15.12707, as well as inputs and reference mass balance measurements. For this glacier, no velocity is measured, but the *dH/dt* values closely correspond to the reference measurements.

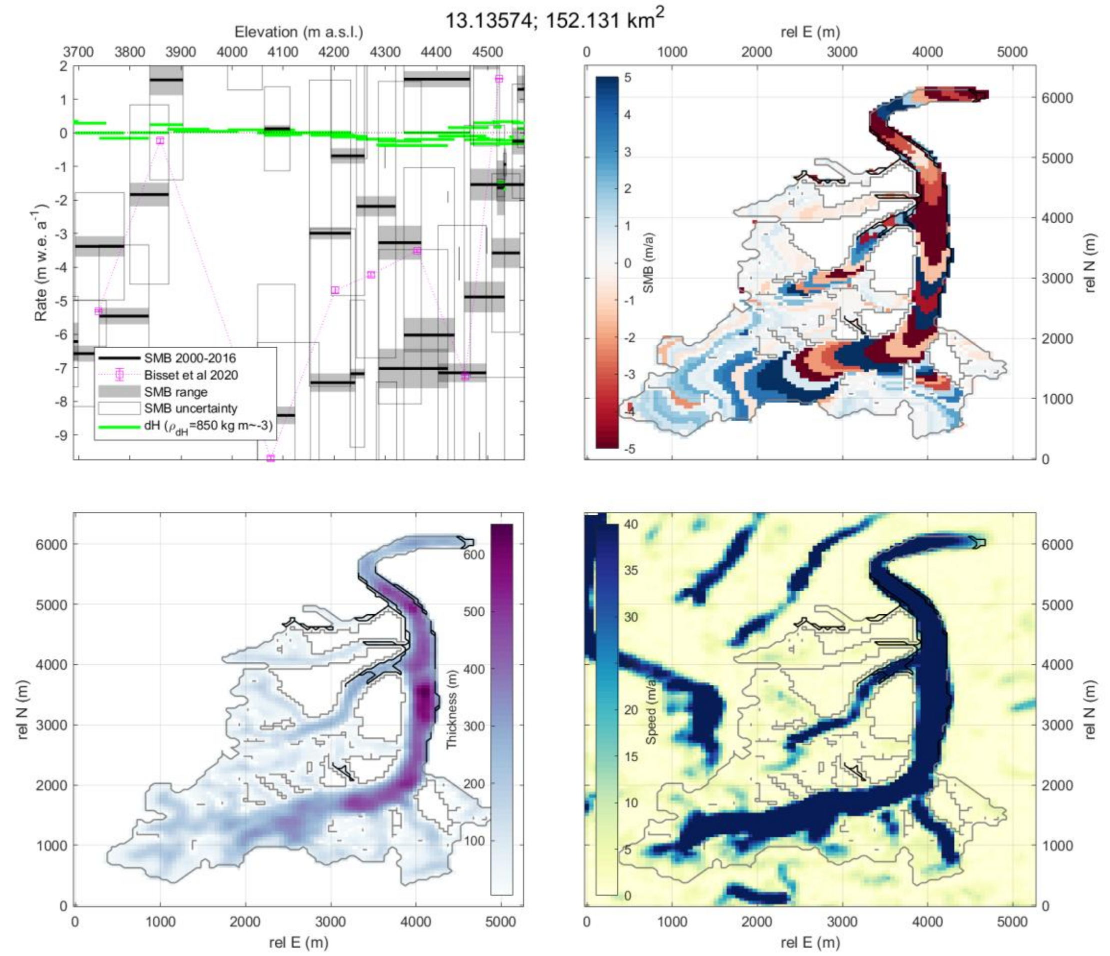


Supplementary Figure 61. Results for glacier 13.13574, as well as inputs and results of B2020. This glacier was removed from our analysis due to its surging appearance, but our results agree closely with those of B2020.

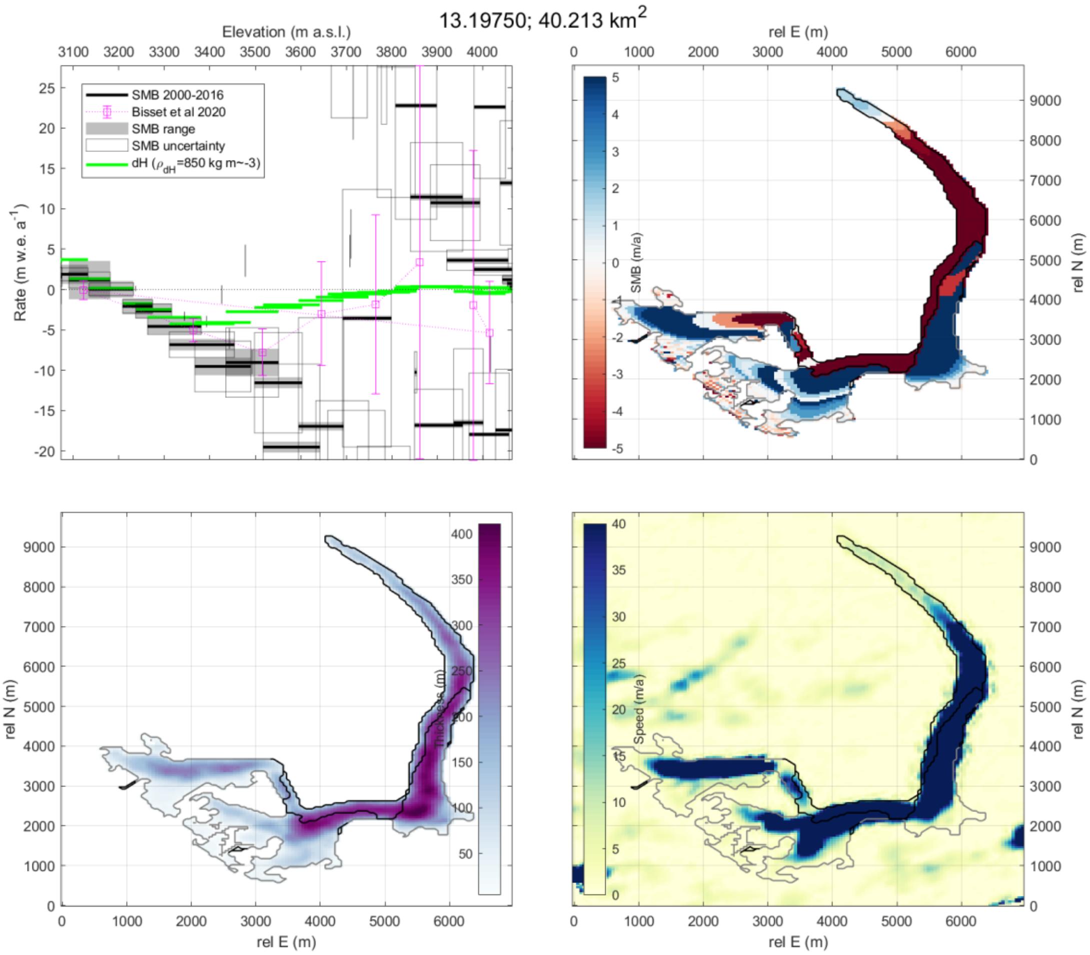


Supplementary Figure 62. Results for glacier 13.19750, as well as inputs and results of B2020. This glacier was removed from our analysis due to its surging appearance, but our results agree closely with those of B2020.

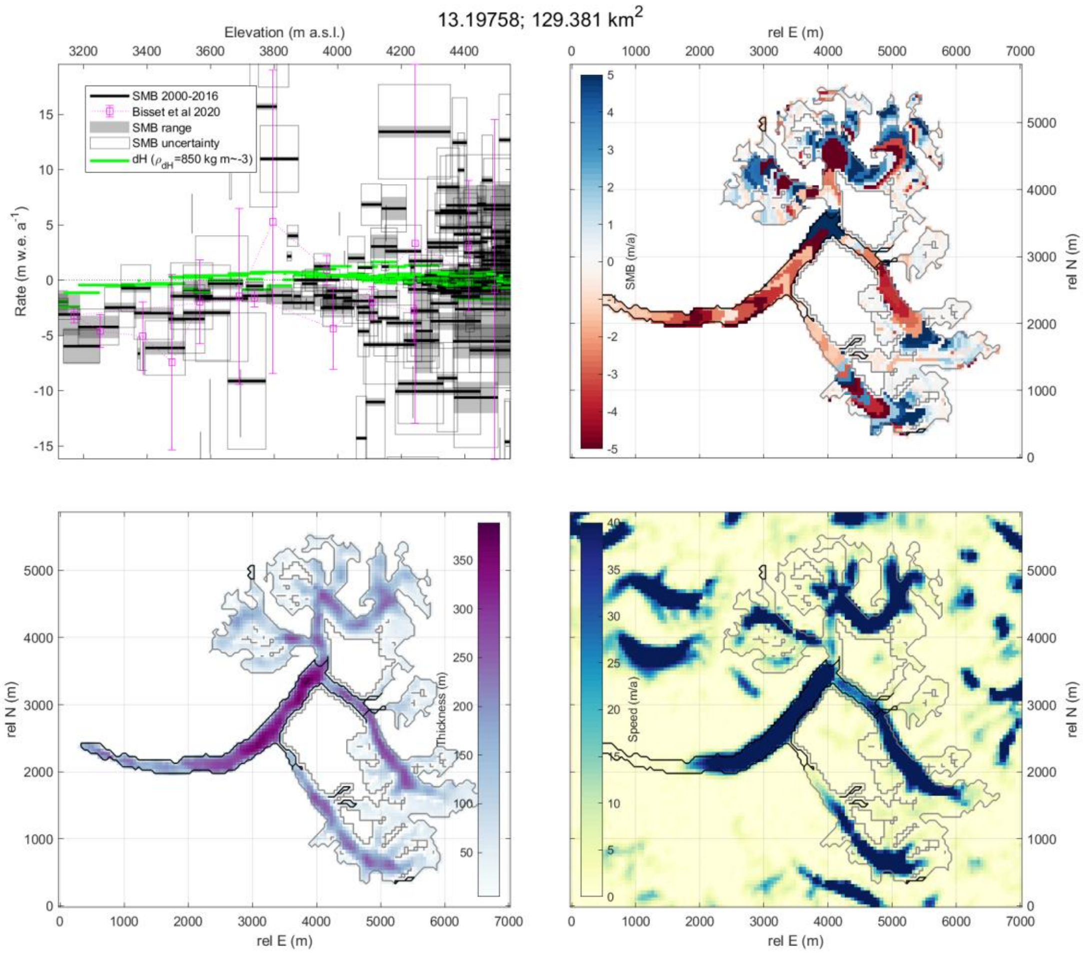


Supplementary Figure 63. Results for glacier 13.19758, as well as inputs and results of B2020. Our results agree closely with those of B2020.

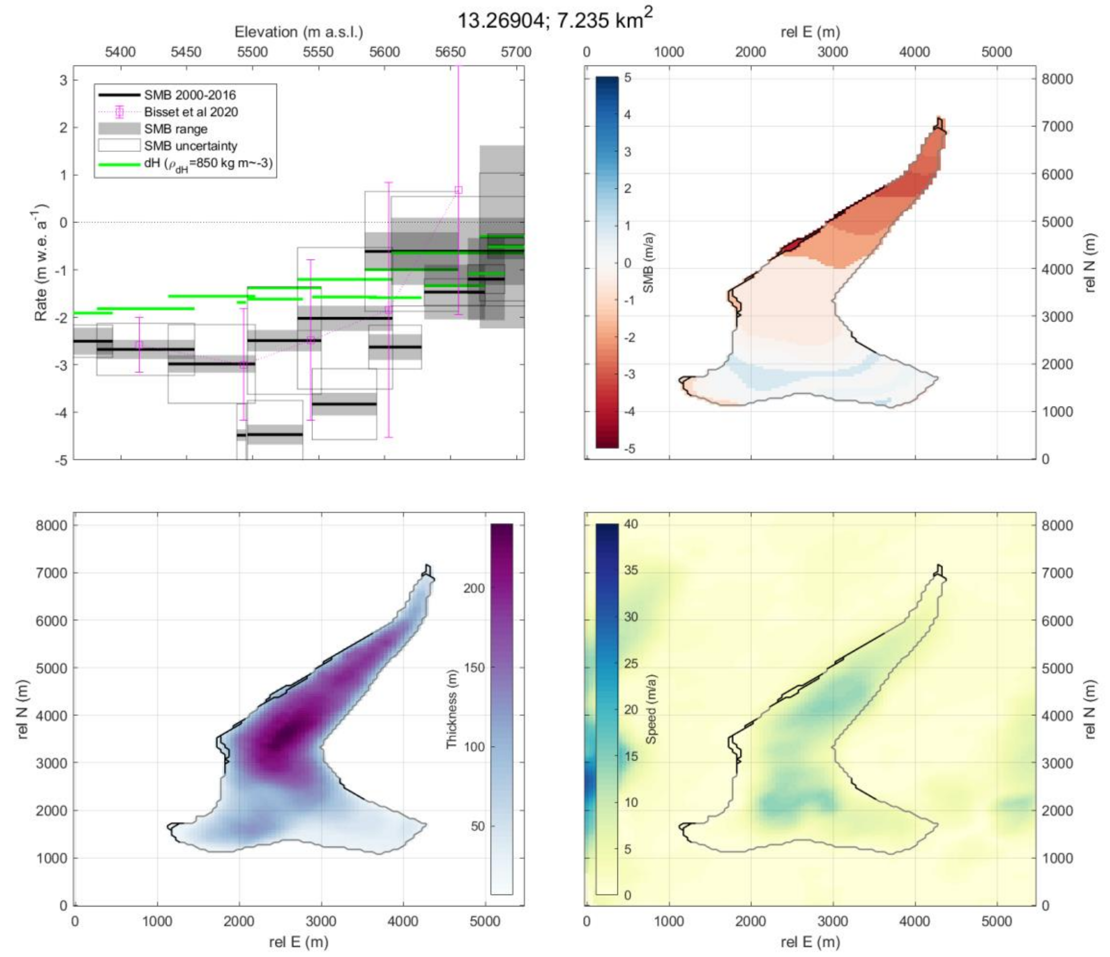


Supplementary Figure 64. Results for glacier 13.26904, as well as inputs and results of B2020. Our results agree closely with those of B2020 with the exception of sliver polygons corresponding to apparent debris cover.

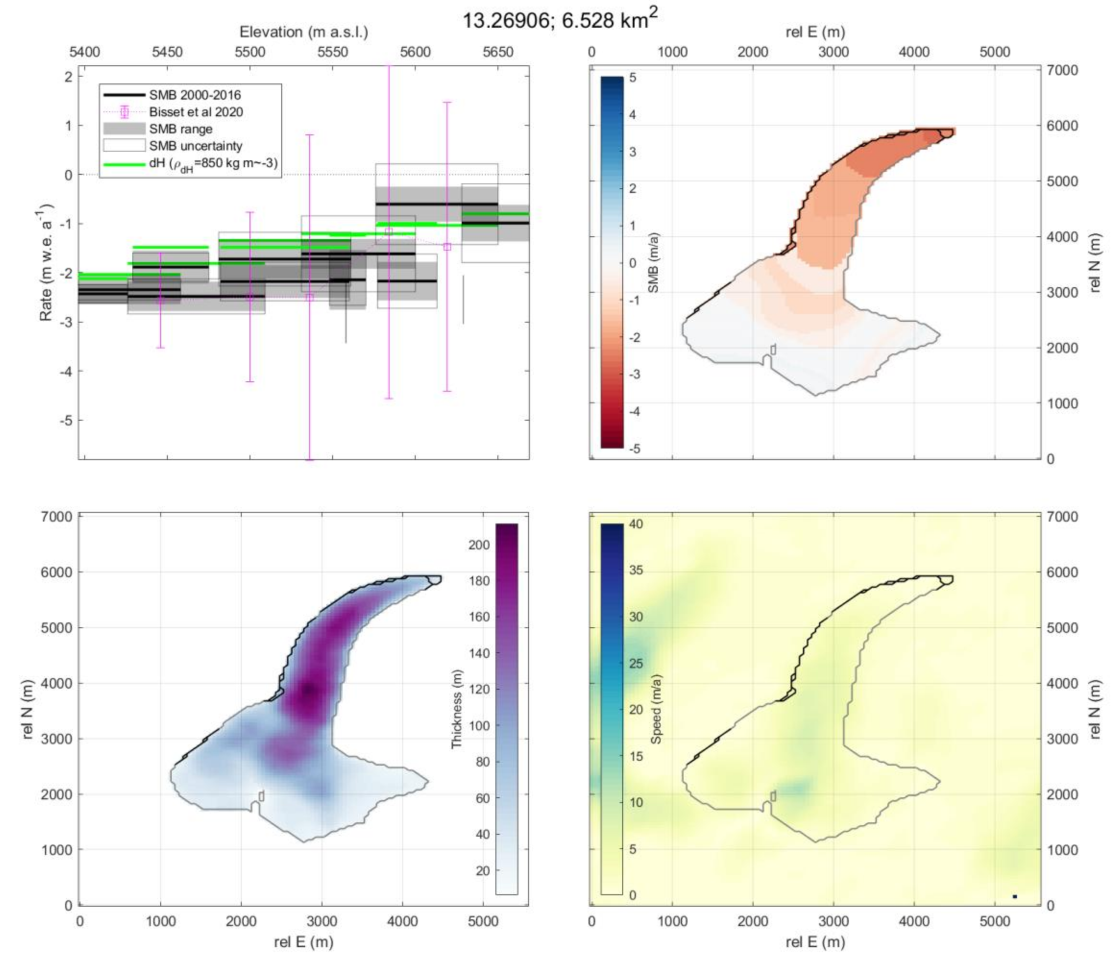


Supplementary Figure 65. Results for glacier 13.26906, as well as inputs and results of B2020. Our results agree closely with those of B2020.

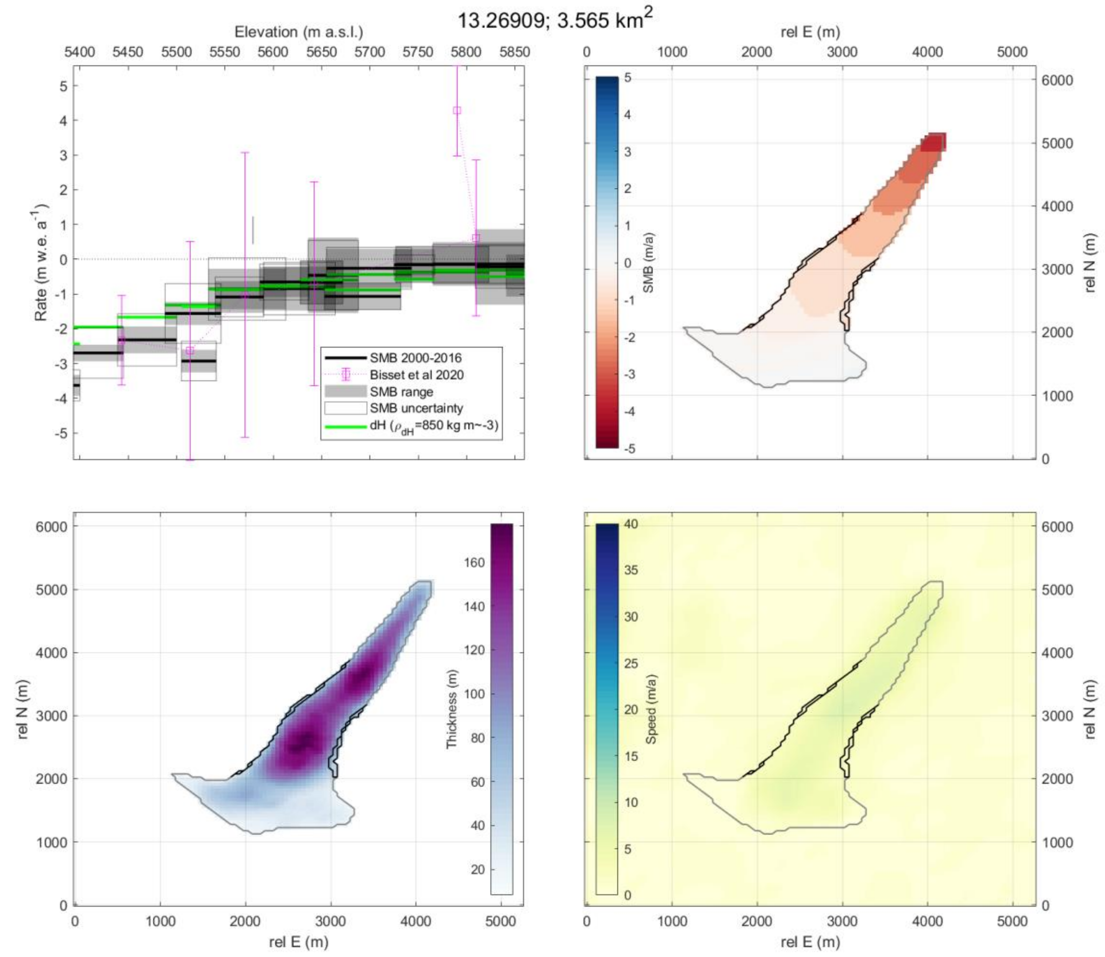


Supplementary Figure 66. Results for glacier 13.26909, as well as inputs and results of B2020. Our results agree closely with those of B2020, and seem to provide a better representation of surface mass balance.

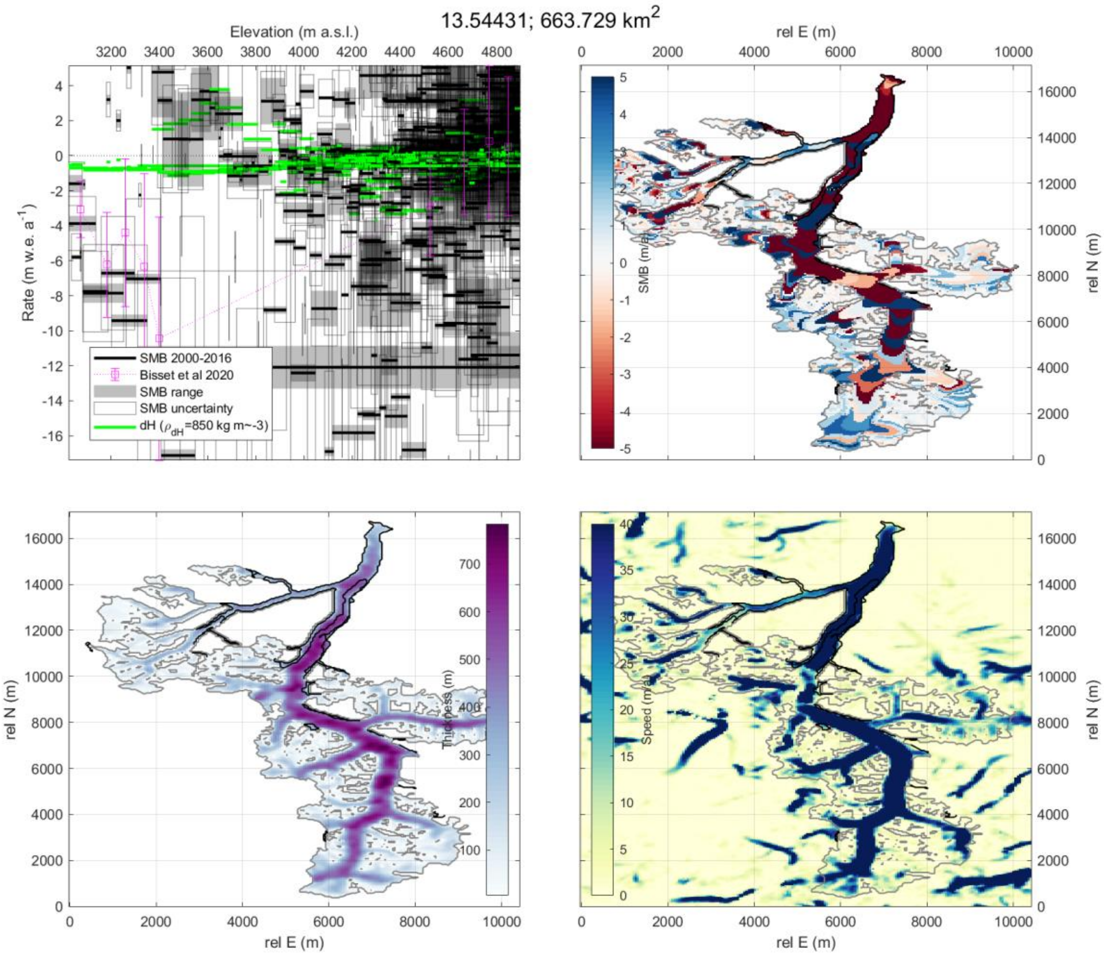


Supplementary Figure 67. Results for glacier 13.54431, as well as inputs and results of B2020. This glacier was removed from our analysis due to its erratic SMB pattern, but our results agree closely with those of B2020.

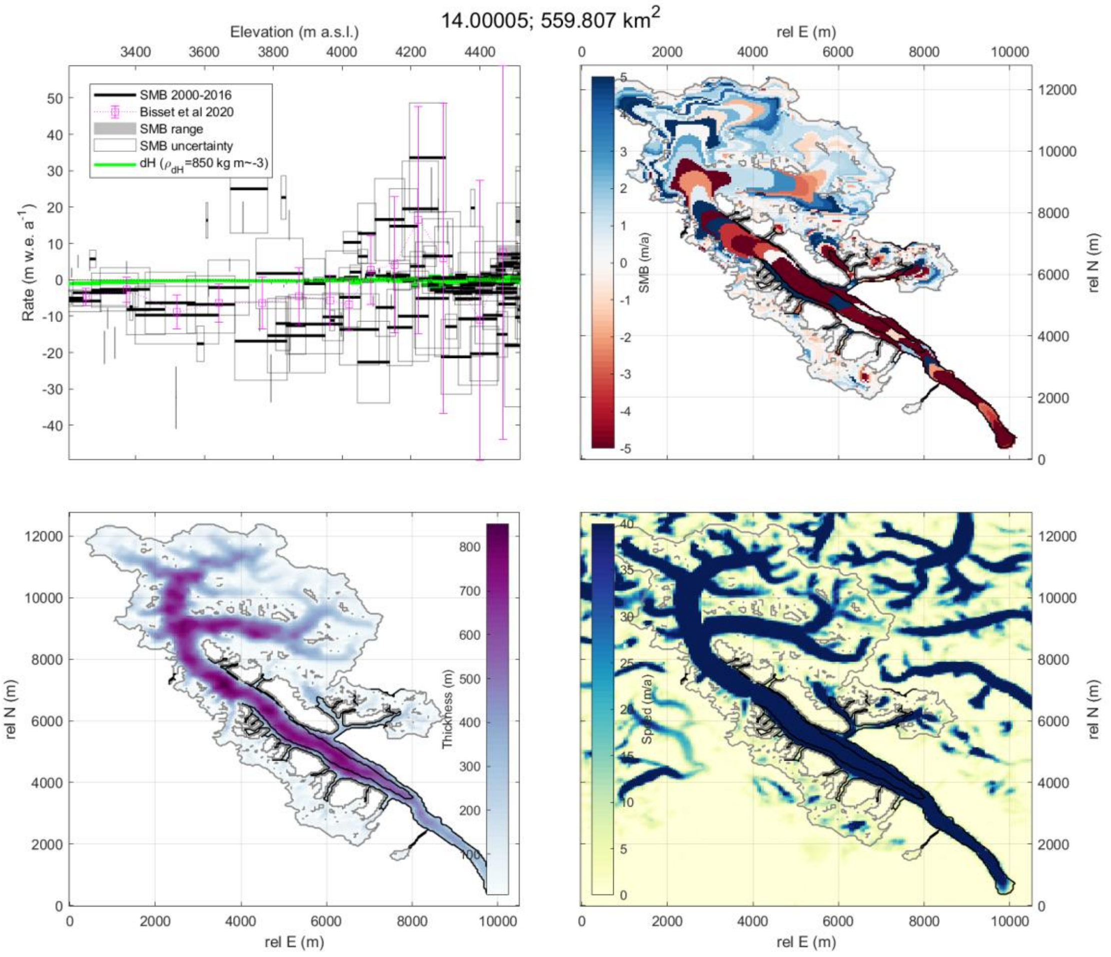


Supplementary Figure 68. Results for glacier 14.00005, as well as inputs and results of B2020. Our results generally agree with those of B2020, but are slightly more erratic due to the automated segmentation scheme.

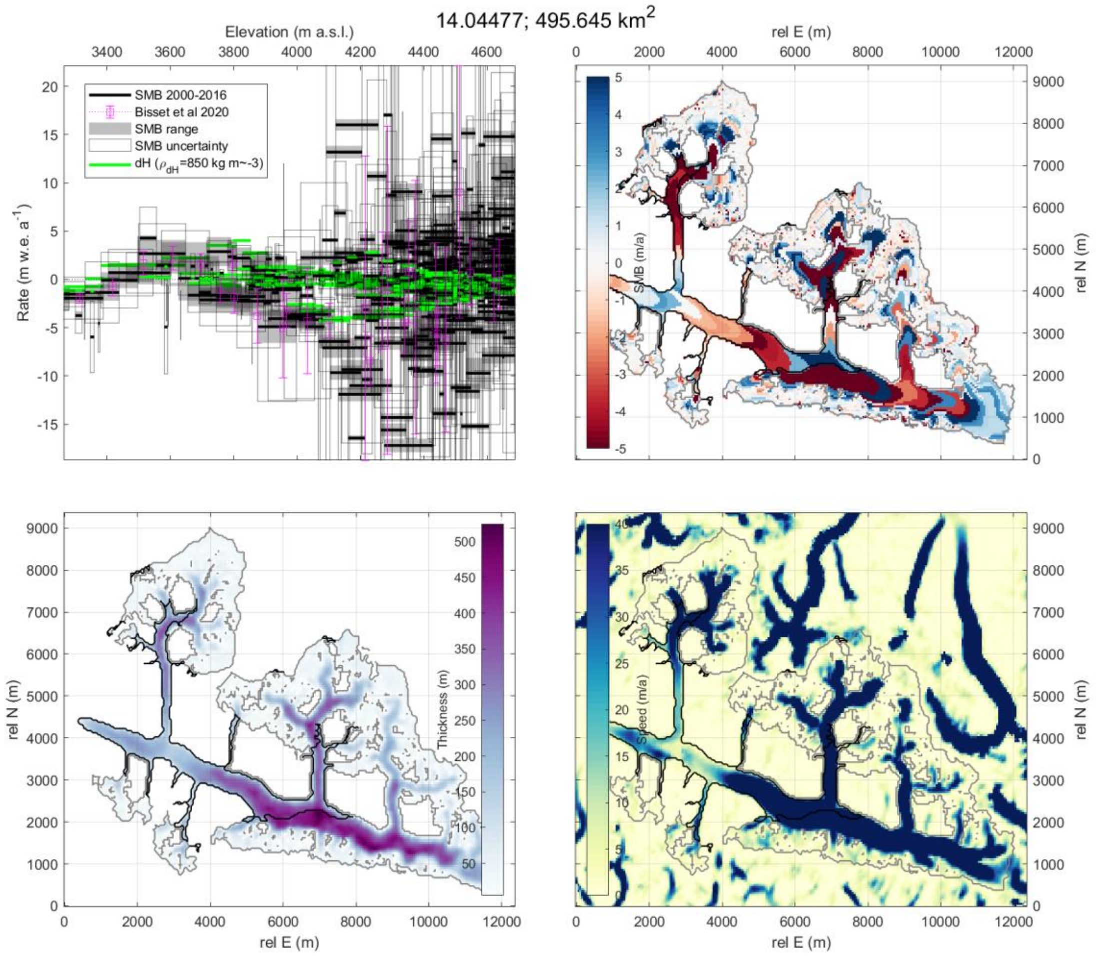


Supplementary Figure 69. Results for glacier 14.04477, as well as inputs and results of B2020. This glacier was removed from our analysis as it is surging. Our results agree closely with those of B2020, but are slightly more erratic due to the automated segmentation scheme.

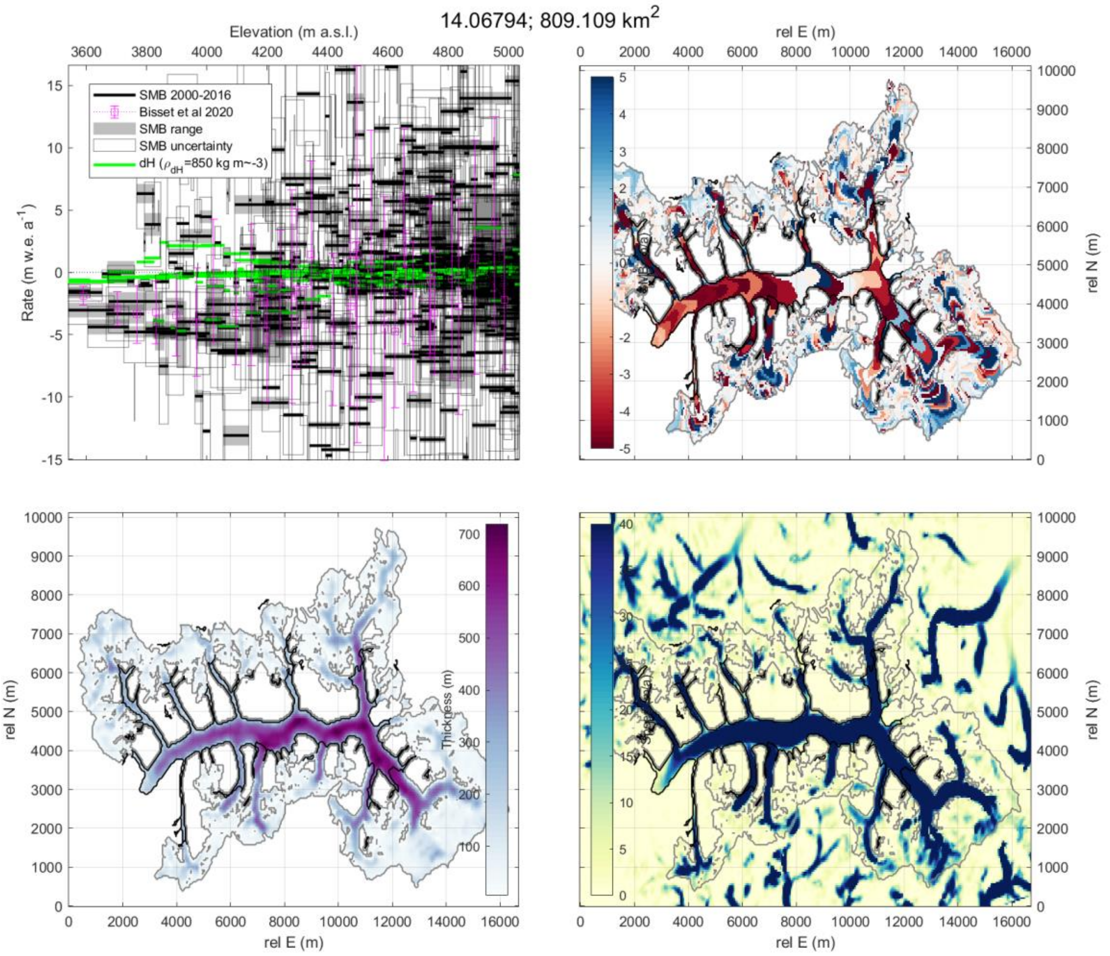


Supplementary Figure 70. Results for glacier 14.06794, as well as inputs and results of B2020. This glacier was removed from our analysis as it has surging tributaries, Our results agree with those of B2020, but are slightly more erratic due to the representation of SMB along all tributaries rather than just the main glacier trunk.

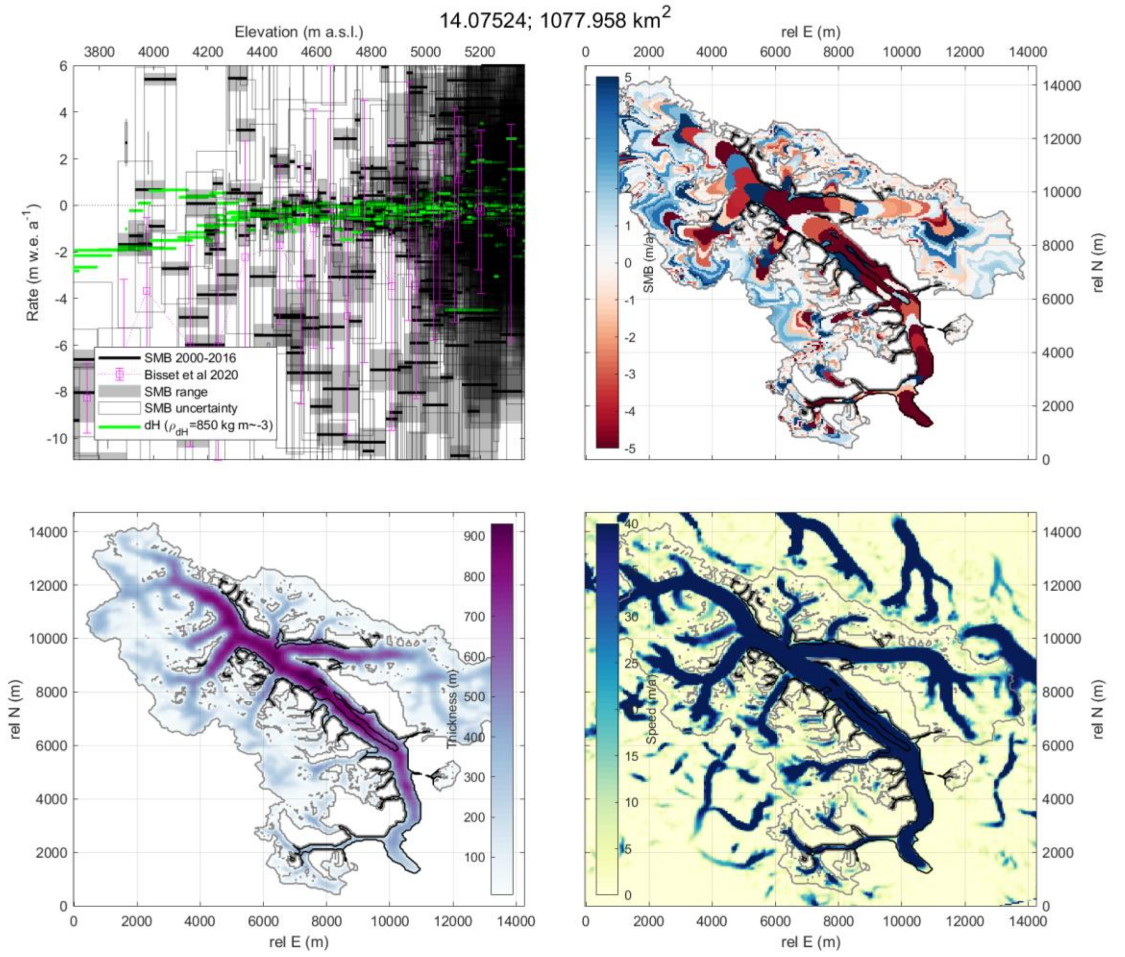


Supplementary Figure 71. Results for glacier 14.07524, as well as inputs and results of B2020. This glacier was removed from our analysis as its results were somewhat erratic. Our results agree with those of B2020 in terms of both pattern and magnitude of SMB, but are slightly more variable. We note that much of the variability in the upper-left plot is attributable to small segments along glacier tributaries, which were not resolved in the B2020 study.

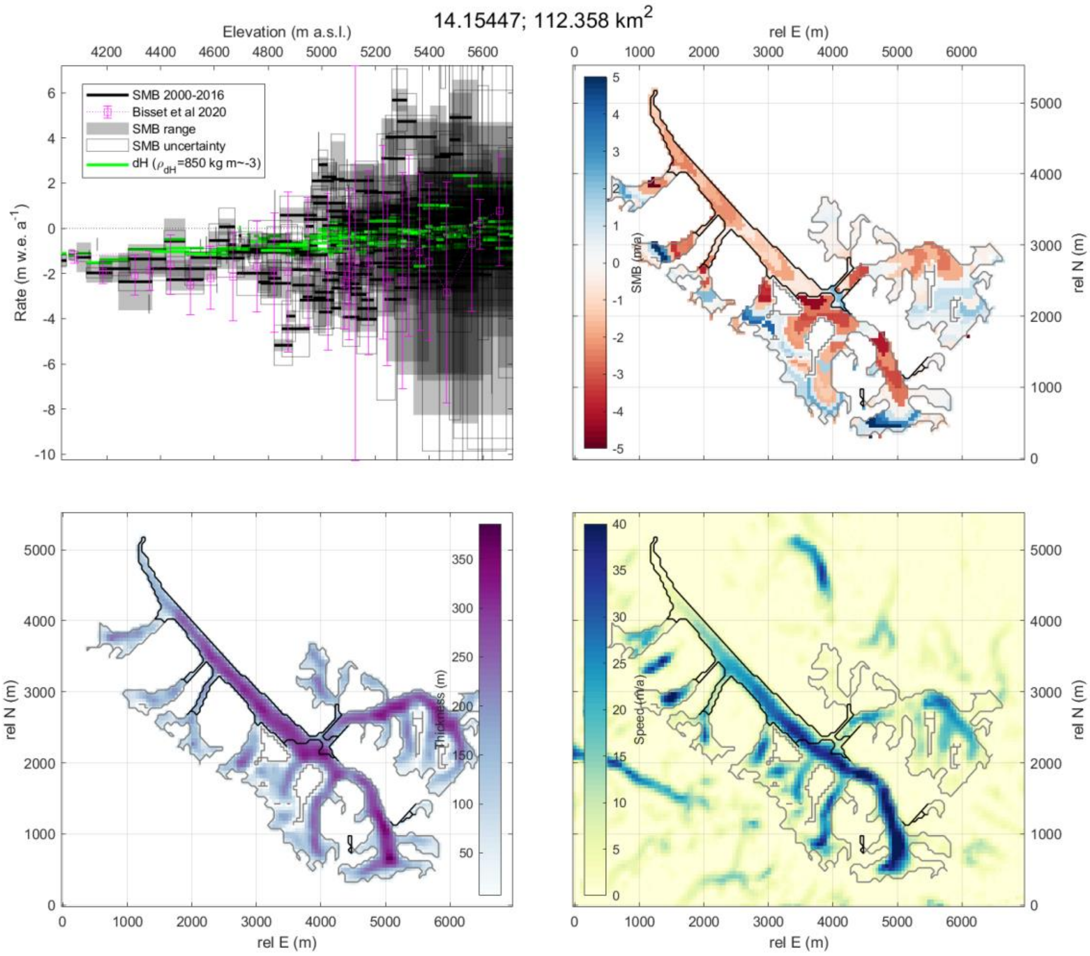


Supplementary Figure 72. Results for glacier 14.15447, as well as inputs and results of B2020. Our results agree with those of B2020 in terms of both pattern and magnitude of SMB, but are slightly more erratic.

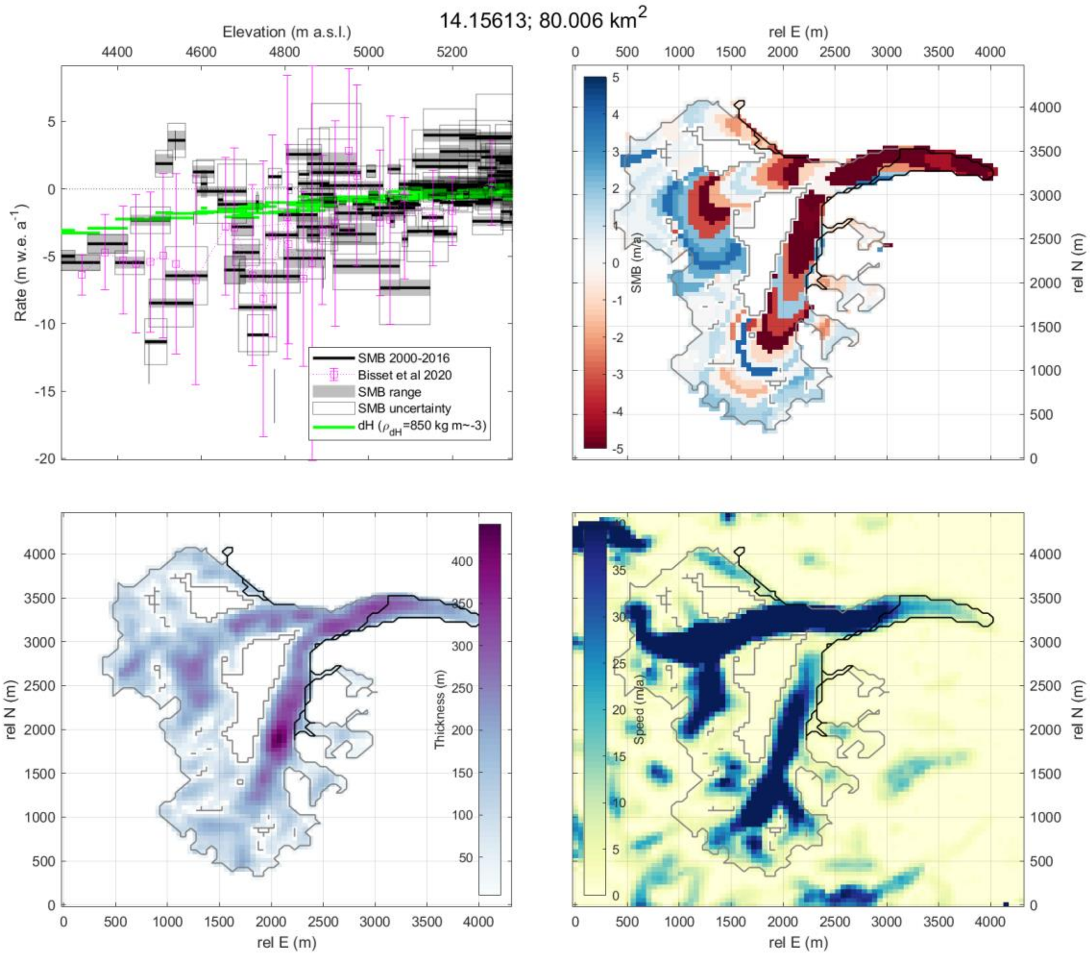


Supplementary Figure 73. Results for glacier 14.15613, as well as inputs and results of B2020. Our results agree with those of B2020 in terms of both pattern and magnitude of SMB. The appearance of disagreement is primarily due to small debris-covered areas at the glacier's margin; the results are otherwise in close agreement.

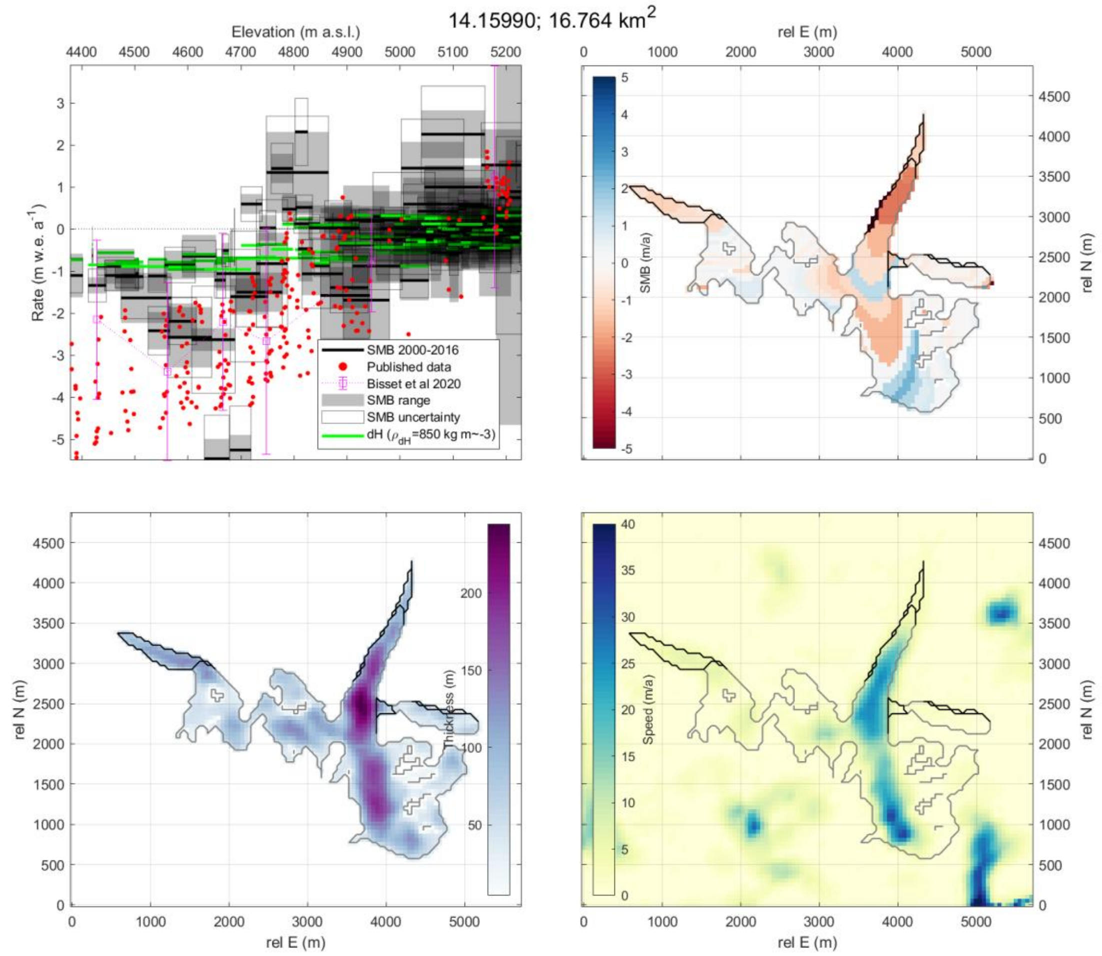


Supplementary Figure 74. Results for glacier 14.15990, as well as inputs, in-situ mass balance measurements, and results of B2020, which correspond to the right of the two glaciers encompassed by this outline. For that flow unit, our results agree with those of B2020 in terms of both pattern and magnitude of SMB. The only disagreement is the small band of apparent positive mass balance at the confluence of two flow units, which was avoided by B2020 by using a manual glacier segmentation.

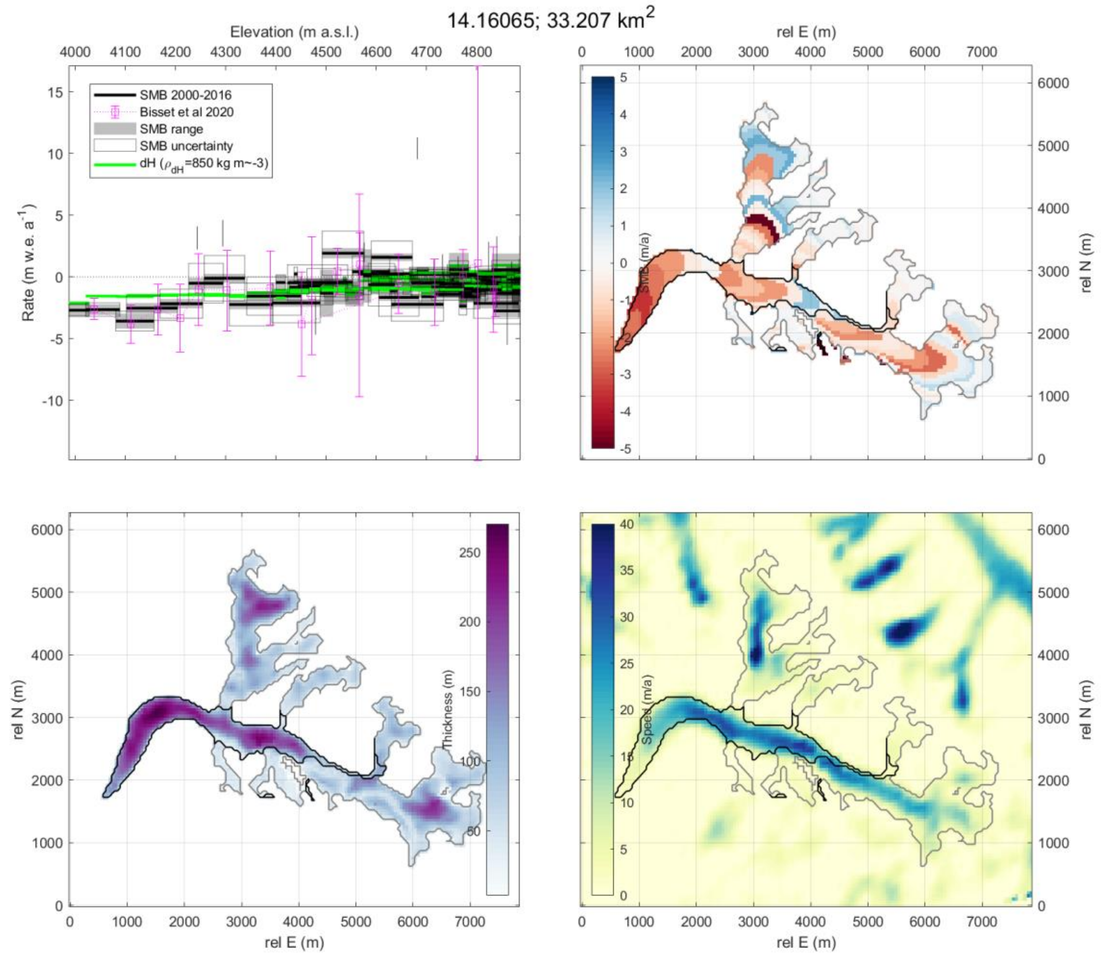


Supplementary Figure 75. Results for glacier 14.16065, as well as inputs and results of B2020. Our results agree closely with those of B2020 in terms of both pattern and magnitude of SMB.

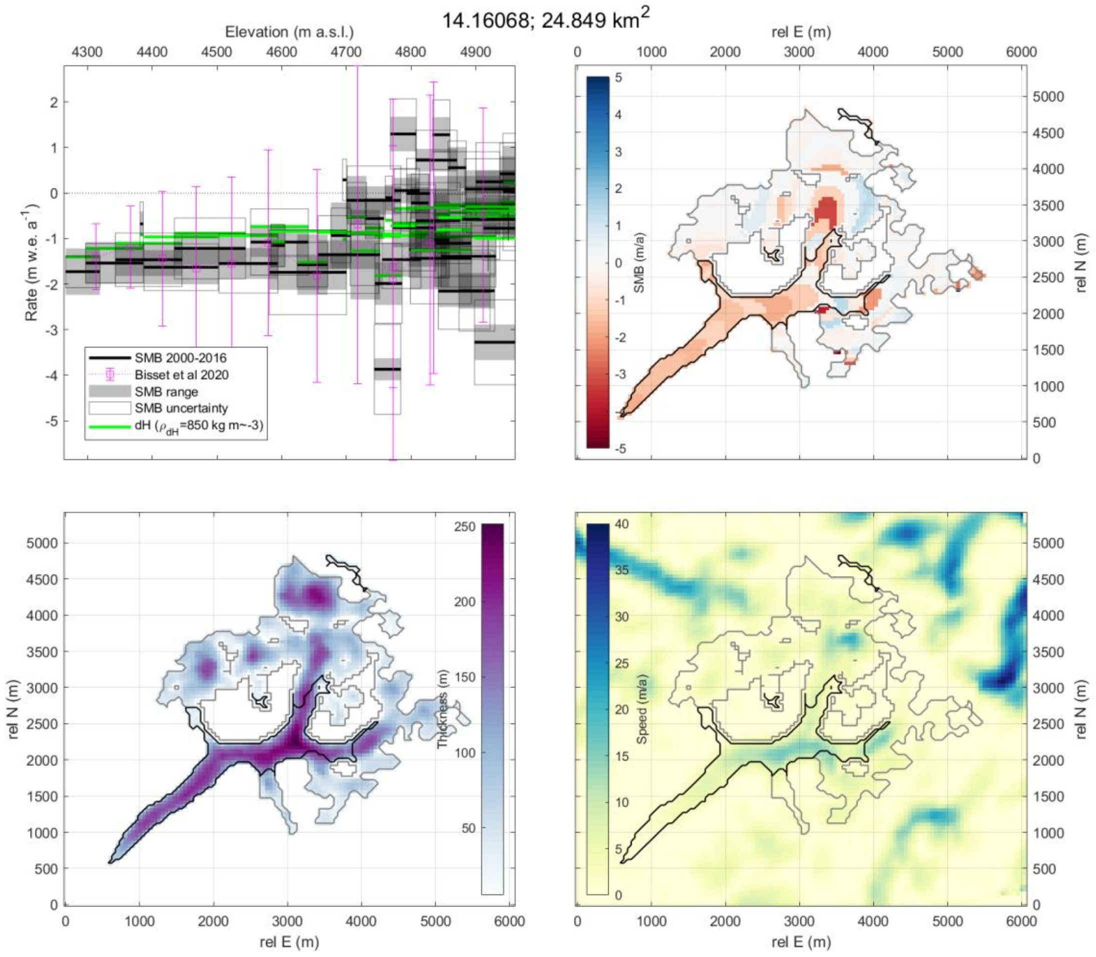


Supplementary Figure 76. Results for glacier 14.16068, as well as inputs and results of B2020. Our results agree closely with those of B2020 in terms of both pattern and magnitude of SMB.

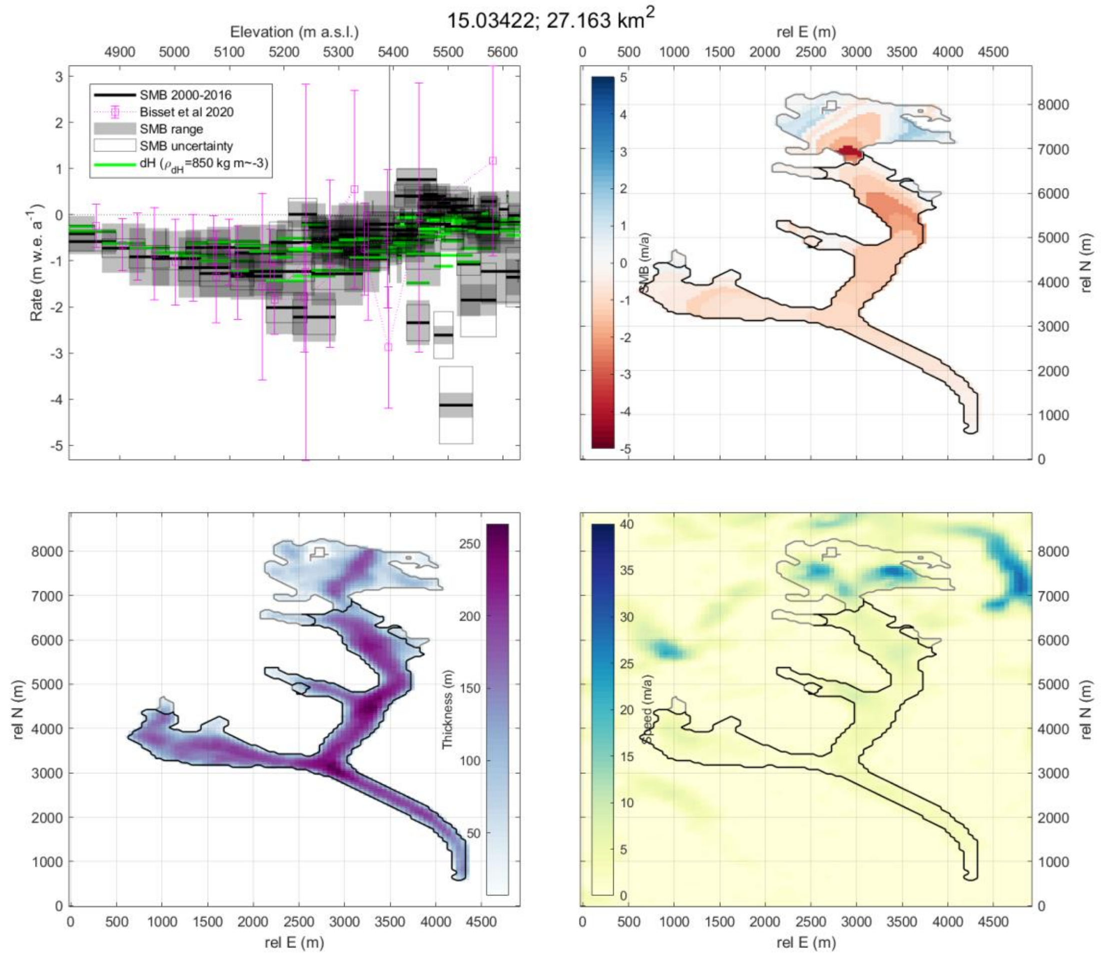


Supplementary Figure 77. Results for glacier 15.03422, as well as inputs and results of B2020. Our results agree closely with those of B2020 in terms of both pattern and magnitude of SMB.

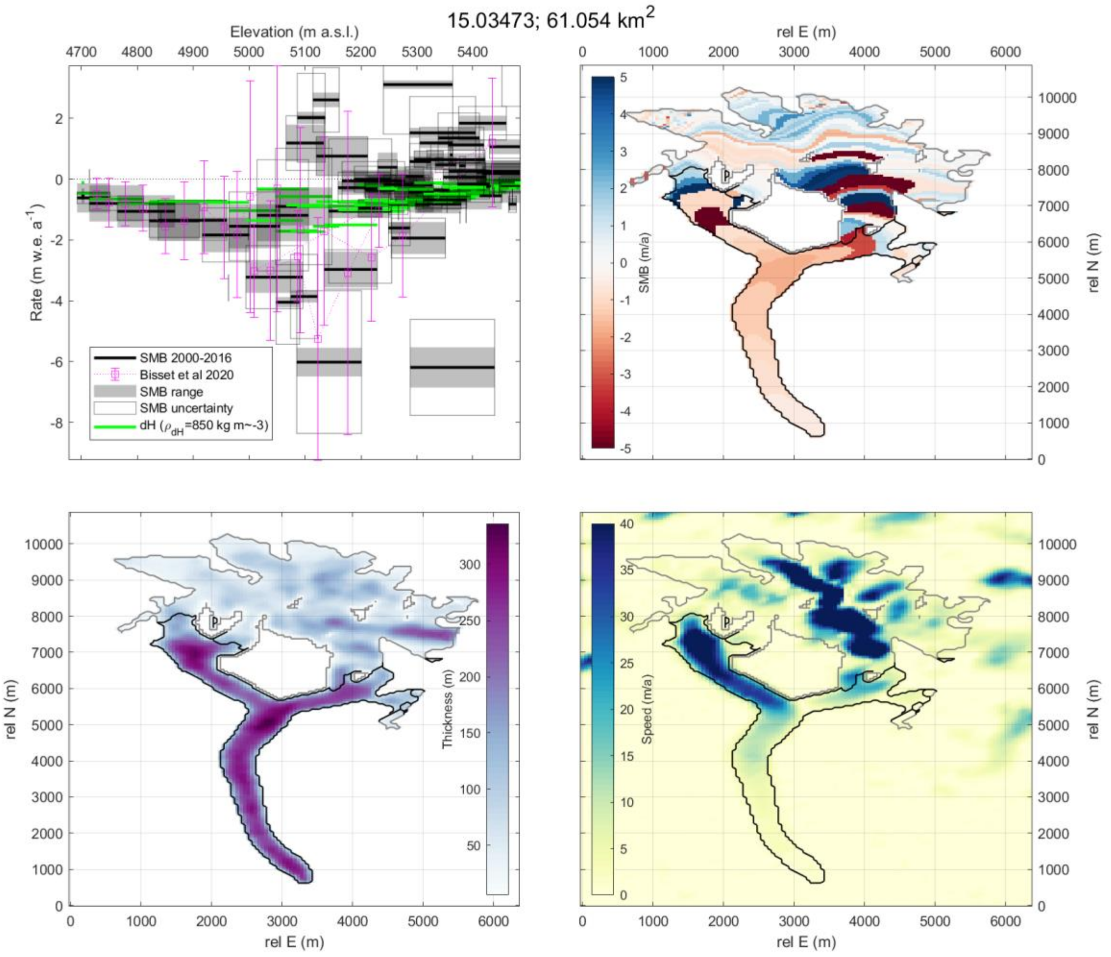


Supplementary Figure 78. Results for glacier 15.03473, as well as inputs and results of B2020. Our results agree closely with those of B2020 in terms of both pattern and magnitude of SMB.

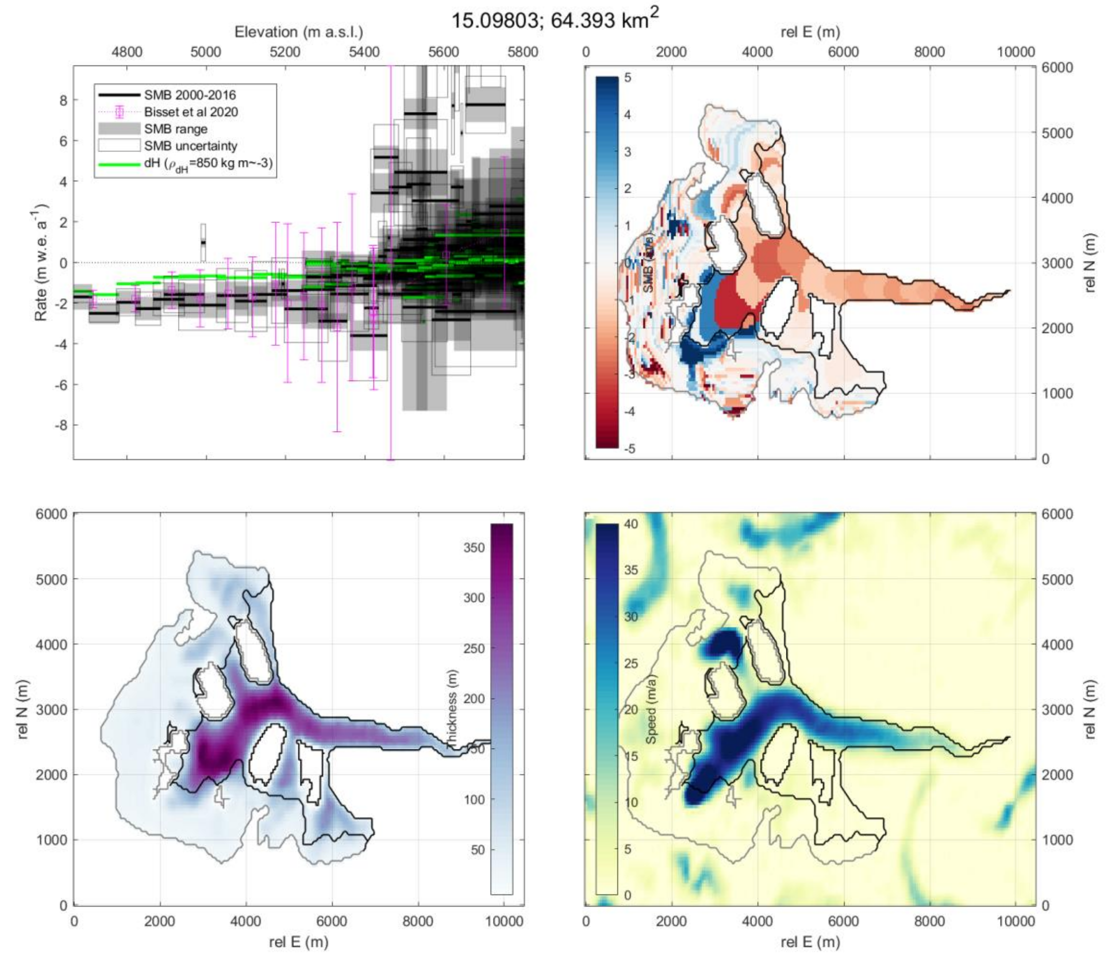


Supplementary Figure 79. Results for glacier 15.09803, as well as inputs and results of B2020. Our results agree closely with those of B2020 in terms of both pattern and magnitude of SMB.

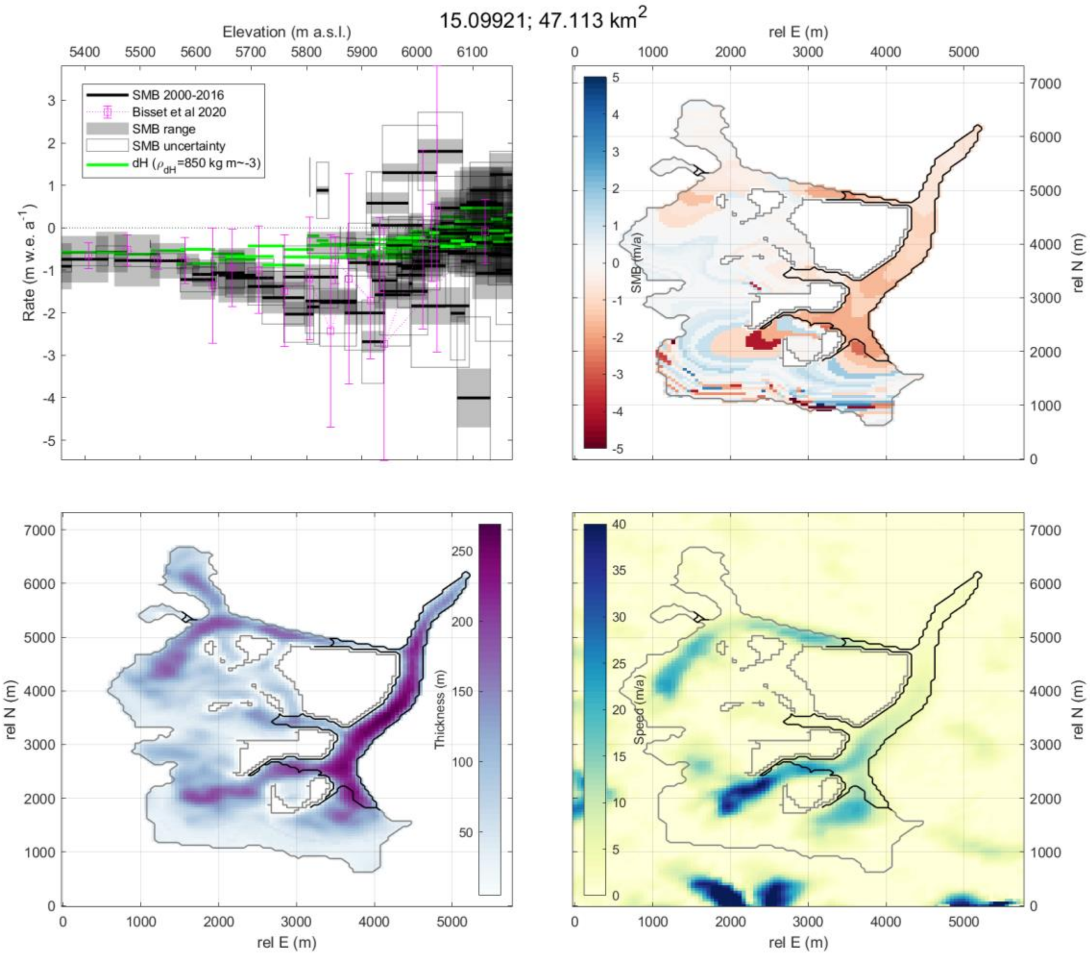


Supplementary Figure 80. Results for glacier 15.09921, as well as inputs and results of B2020. Our results agree closely with those of B2020 in terms of both pattern and magnitude of SMB.

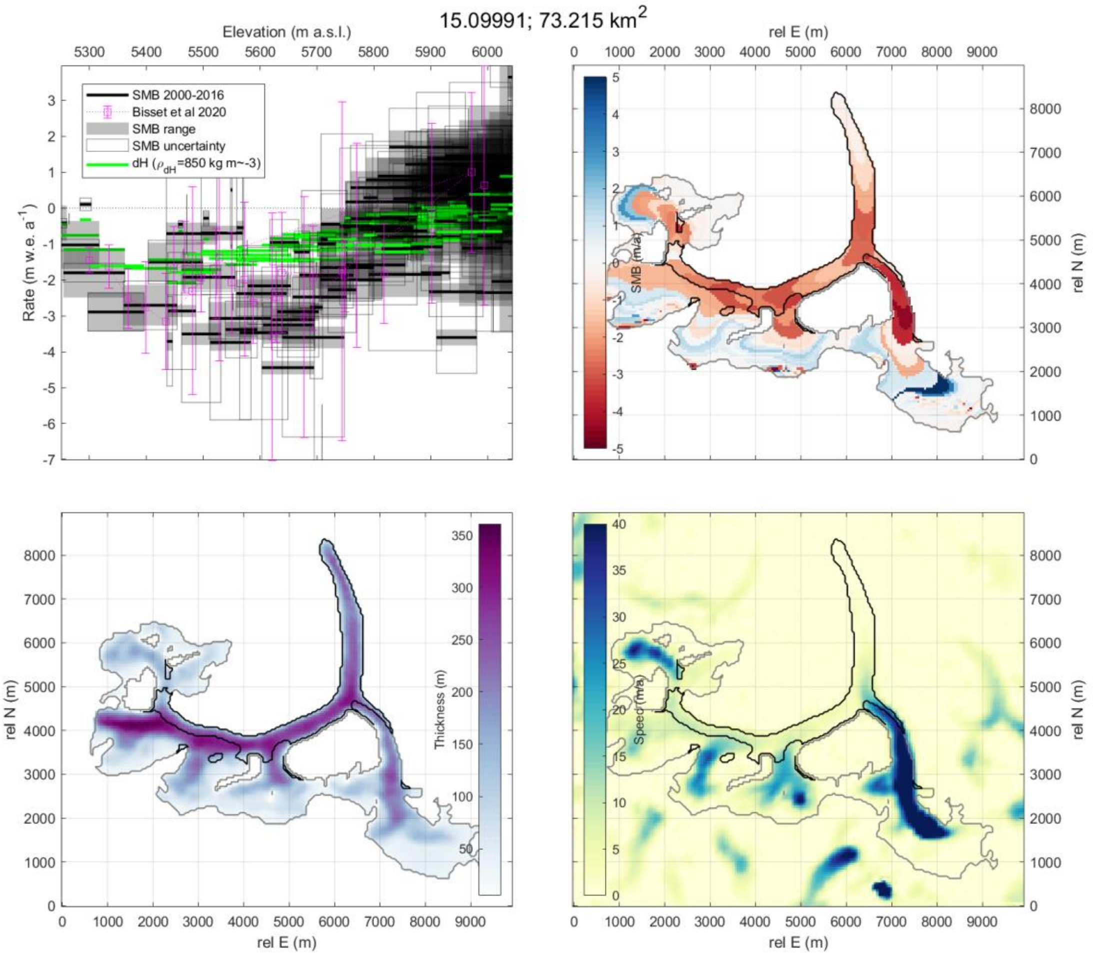


Supplementary Figure 81. Results for glacier 15.09991, as well as inputs and results of B2020. Our results agree closely with those of B2020 in terms of both pattern and magnitude of SMB, and better resolve SMB differences between distinct tributaries.

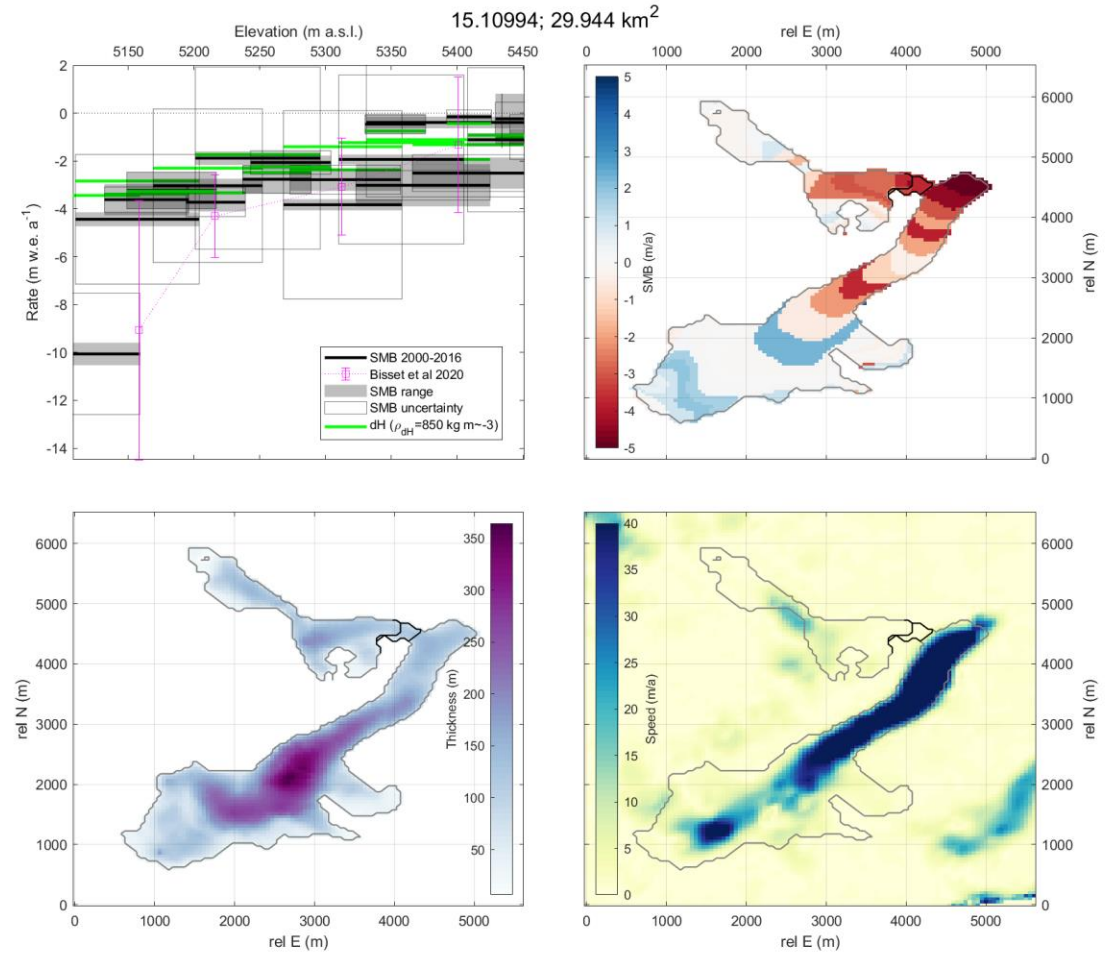


Supplementary Figure 82. Results for glacier 15.03473, as well as inputs and results of B2020. Our results agree closely with those of B2020 in terms of both pattern and magnitude of SMB.

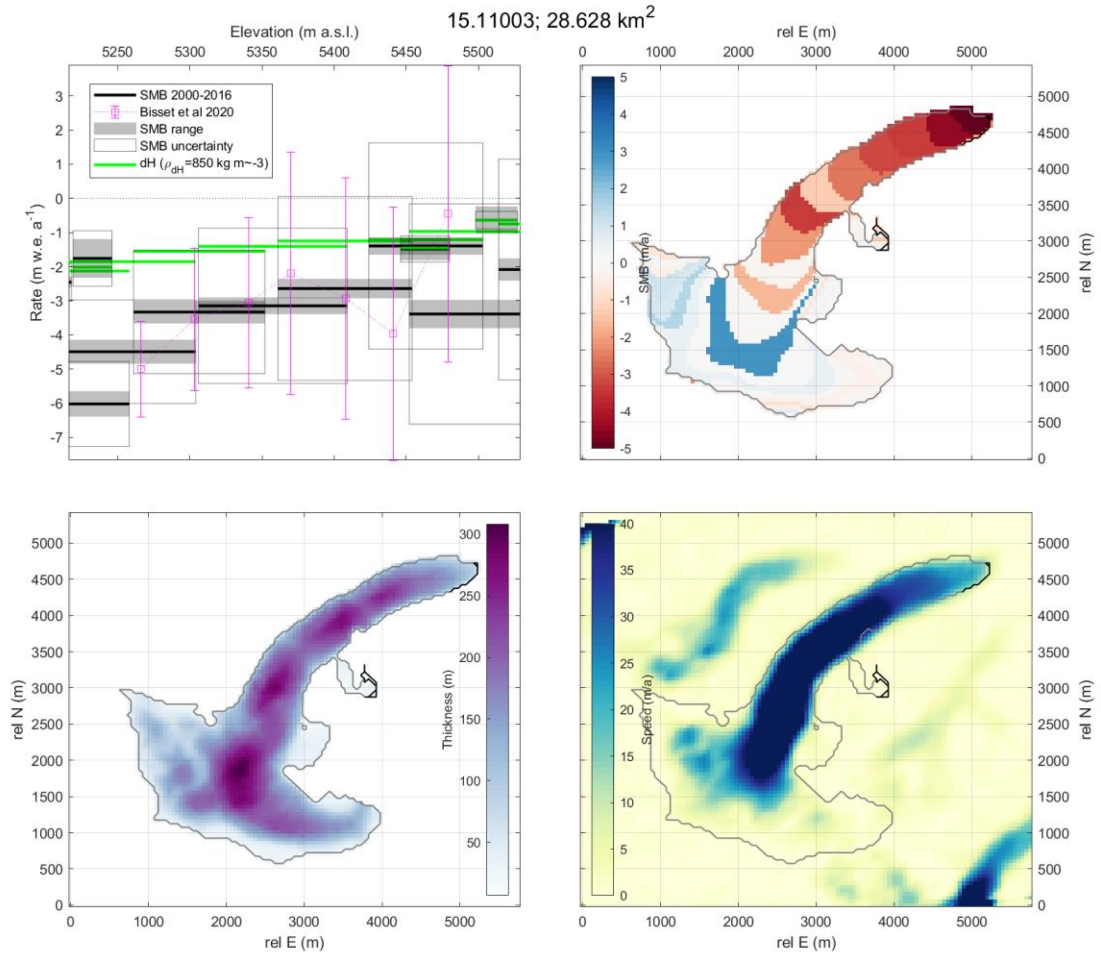


Supplementary Figure 83. Results for glacier 15.03473, as well as inputs and results of B2020. Our results agree closely with those of B2020 in terms of both pattern and magnitude of SMB.

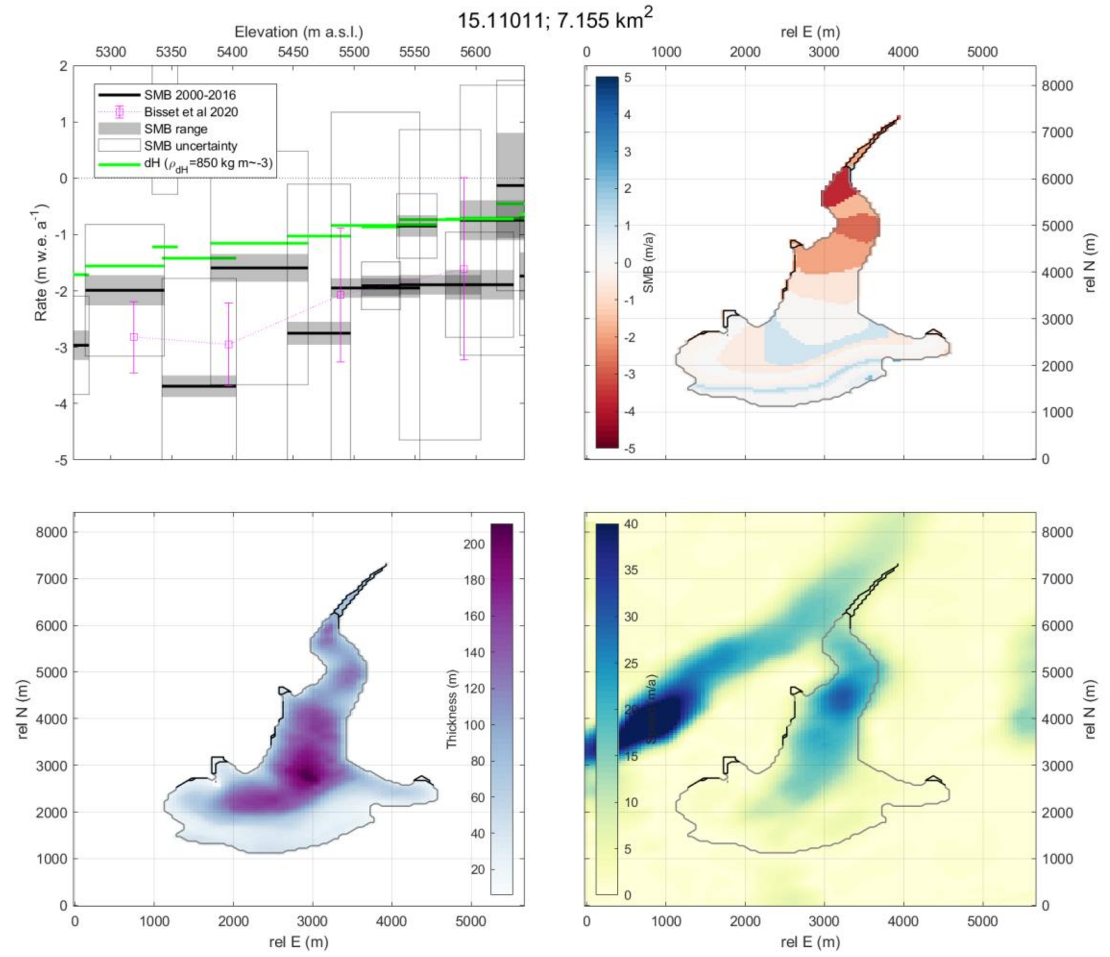


Supplementary Figure 84. Results for glacier 15.11011, as well as inputs and results of B2020. Our results agree closely with those of B2020 in terms of both pattern and magnitude of SMB. The apparent scatter in our results is due to sliver debris-covered segments along the glacier margin.

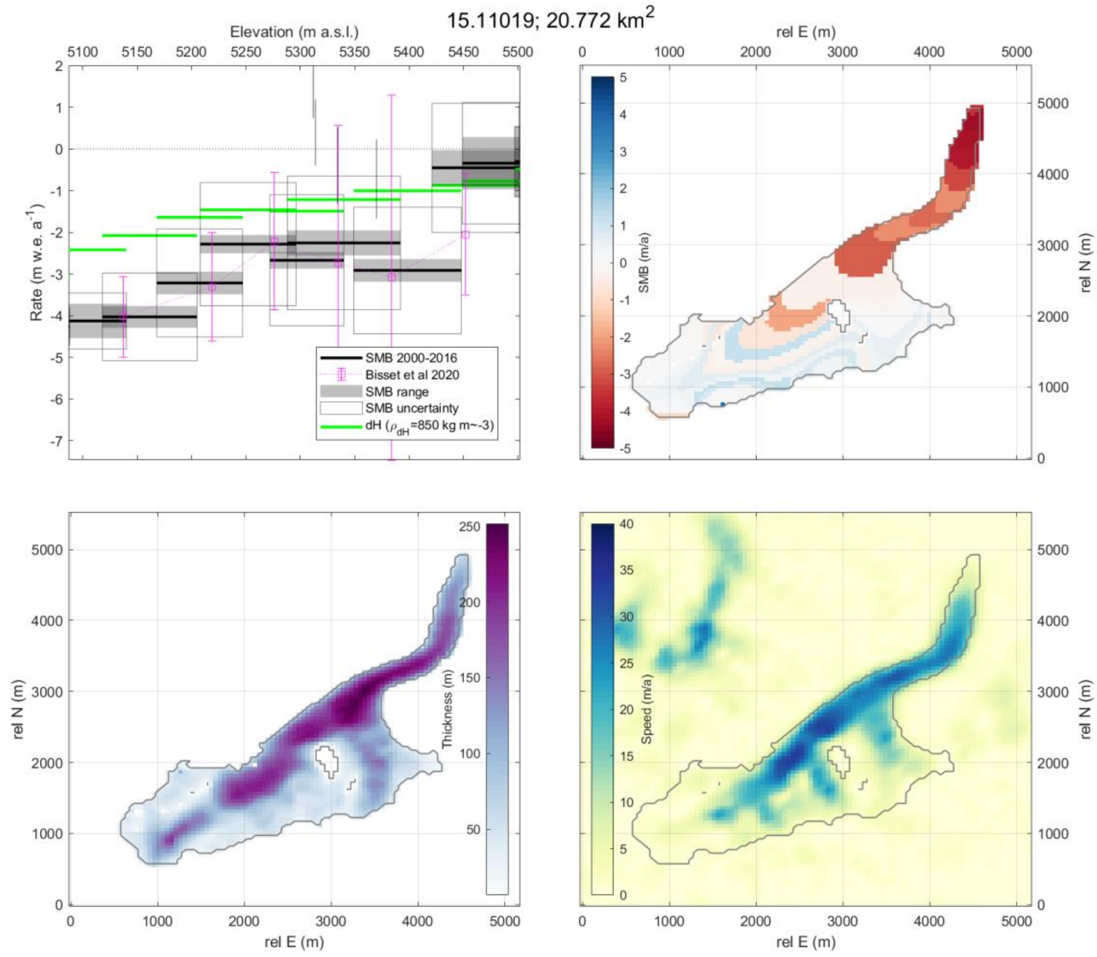


Supplementary Figure 85. Results for glacier 15.11019, as well as inputs and results of B2020. Our results agree closely with those of B2020 in terms of both pattern and magnitude of SMB.

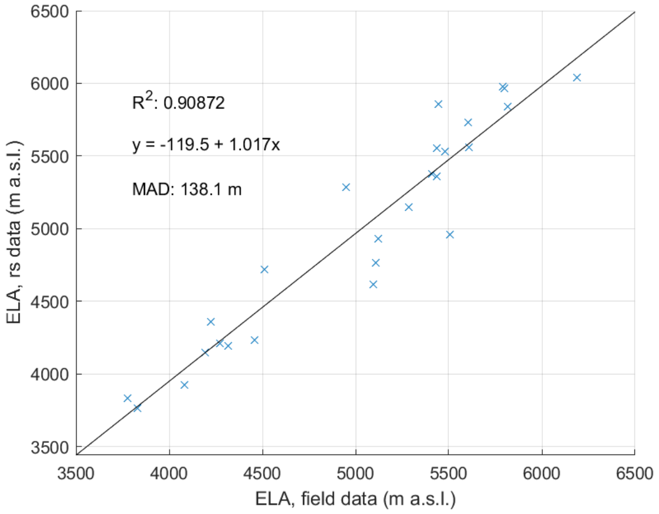


Supplementary Figure 86. ELA values derived from field measurements at reference sites (Supplementary Table 1) compared to results of our analysis.

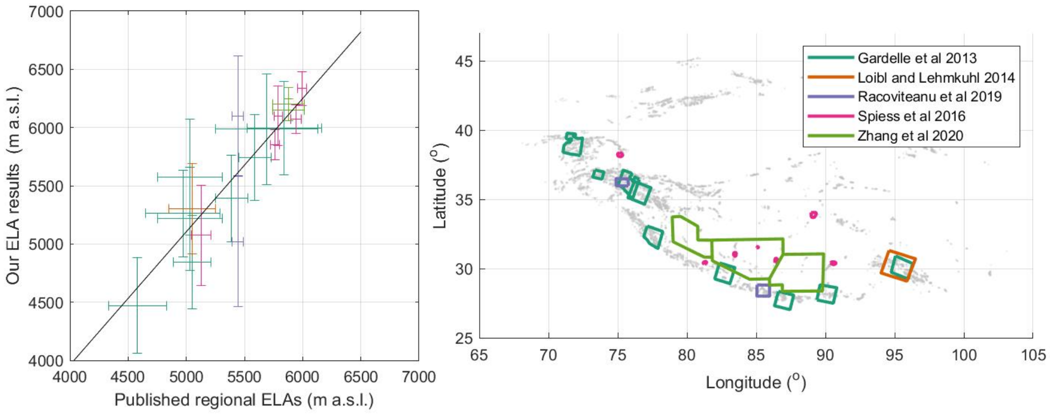


Supplementary Figure 87. ELA results compared to previously published regional estimates by [14–17,71,72].

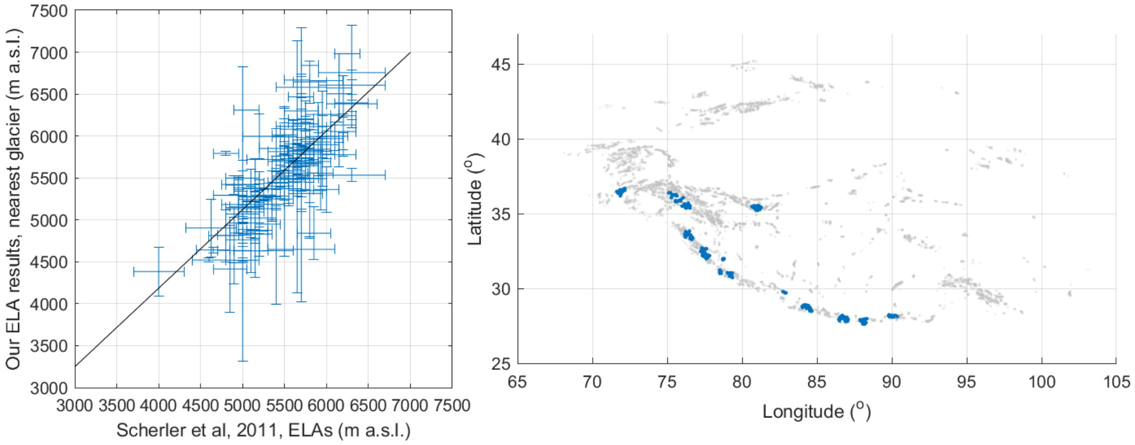


Supplementary Figure 88. ELA results compared to glacier-specific values of [12]. The coordinates provided by [12] did not always correspond to an RGI outline, so here we compare the [12] results to ours for the nearest glaciers, with a search distance limit of 0.5 km.

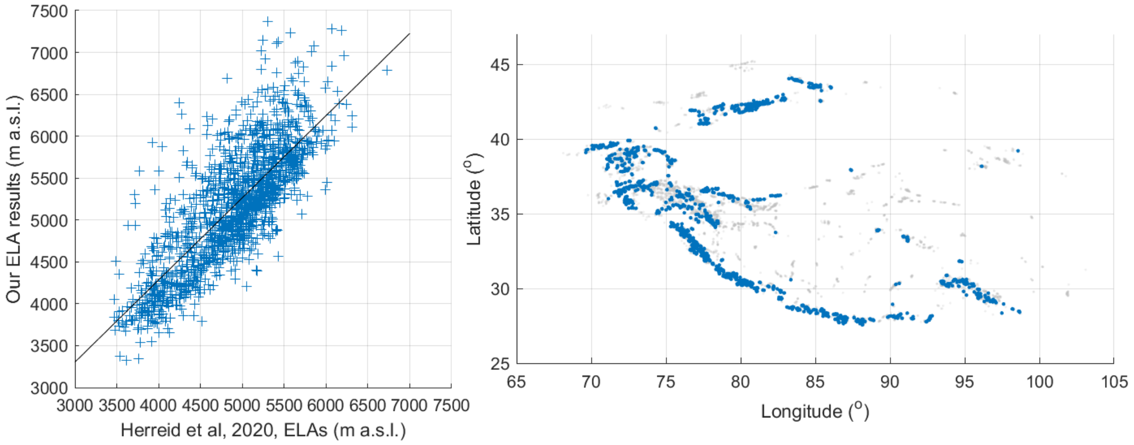


Supplementary Figure 89. ELA results compared to glacier-specific values of [18].

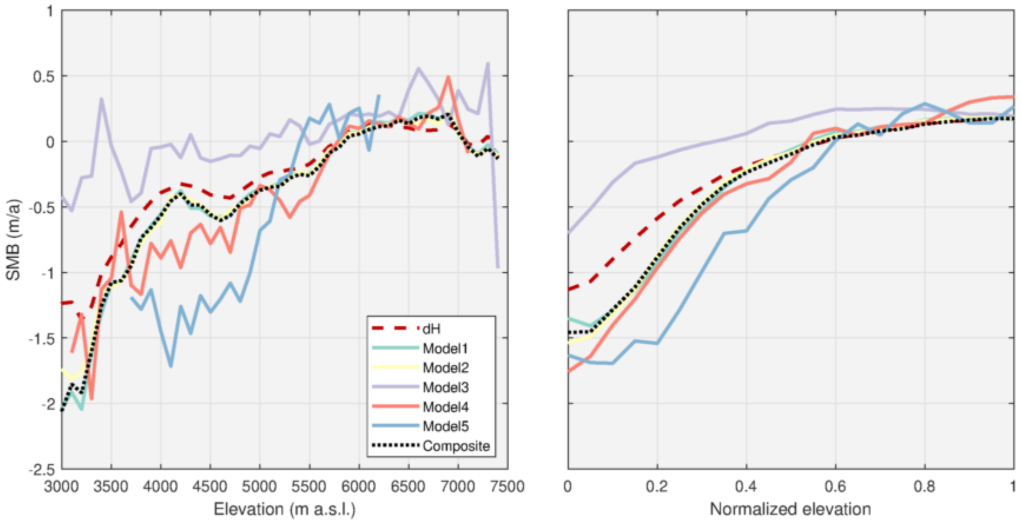


Supplementary Figure 90. Regional mass balance profiles using the continuity method with the five ice thickness models contributing to the consensus [9], with respect to elevation (left) and normalised elevation (right). Note that Models 3-5 do not cover the entire region, so the aggregated profiles differ substantially.

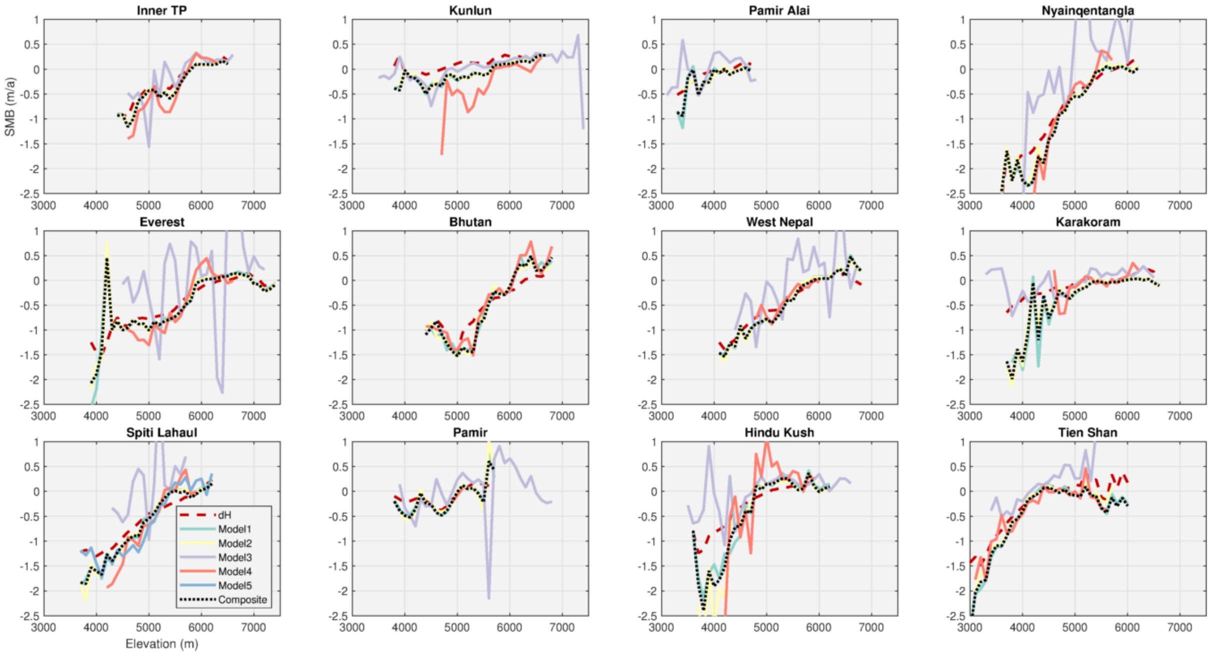


Supplementary Figure 91. Subregional mass balance profiles using the continuity method with the five ice thickness models contributing to the consensus [9]. Note that Models 3-5 do not cover the entire region.

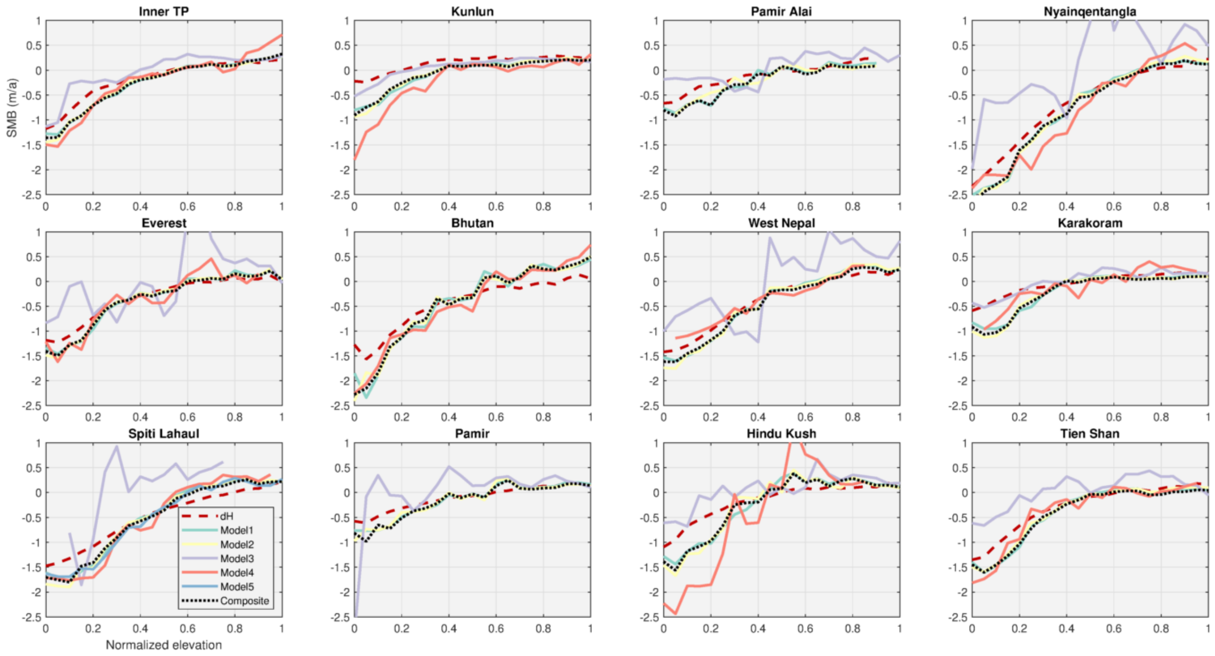


Supplementary Figure 92. Subregional mass balance vs normalized elevation using the continuity method with the five ice thickness models contributing to the consensus [9]. Note that Models 3-5 do not cover the entire region.

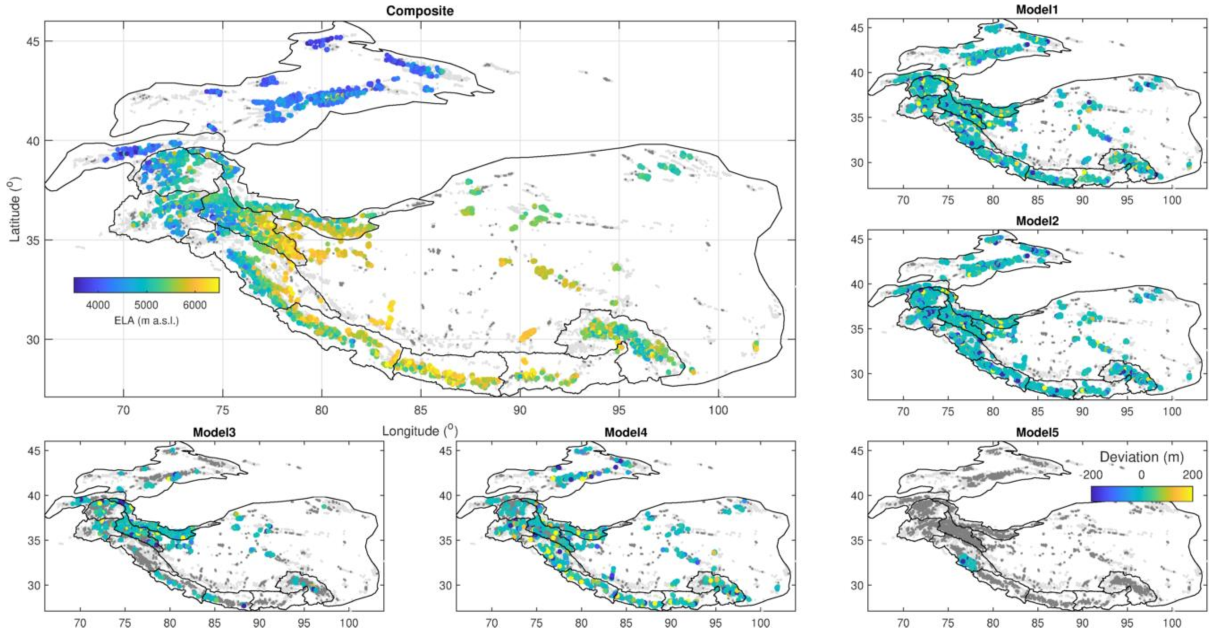


Supplementary Figure 93. ELA estimates based on the consensus ice thickness (upper left, reproduced from Figure 2), along with ELA deviations for the five ice thickness models contributing to the consensus. Note that Models 3-5 do not cover the entire region.

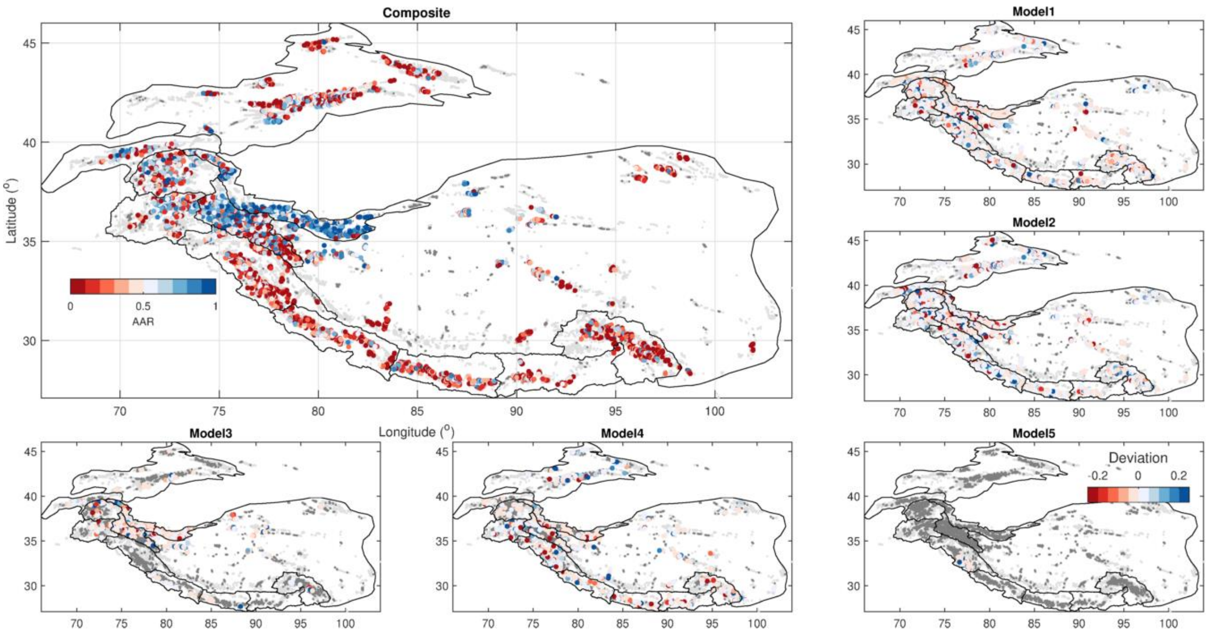


Supplementary Figure 94. AAR estimates based on the consensus ice thickness (upper left, reproduced from Figure 2), along with AAR deviations for the five ice thickness models contributing to the consensus. Note that Models 3-5 do not cover the entire region.

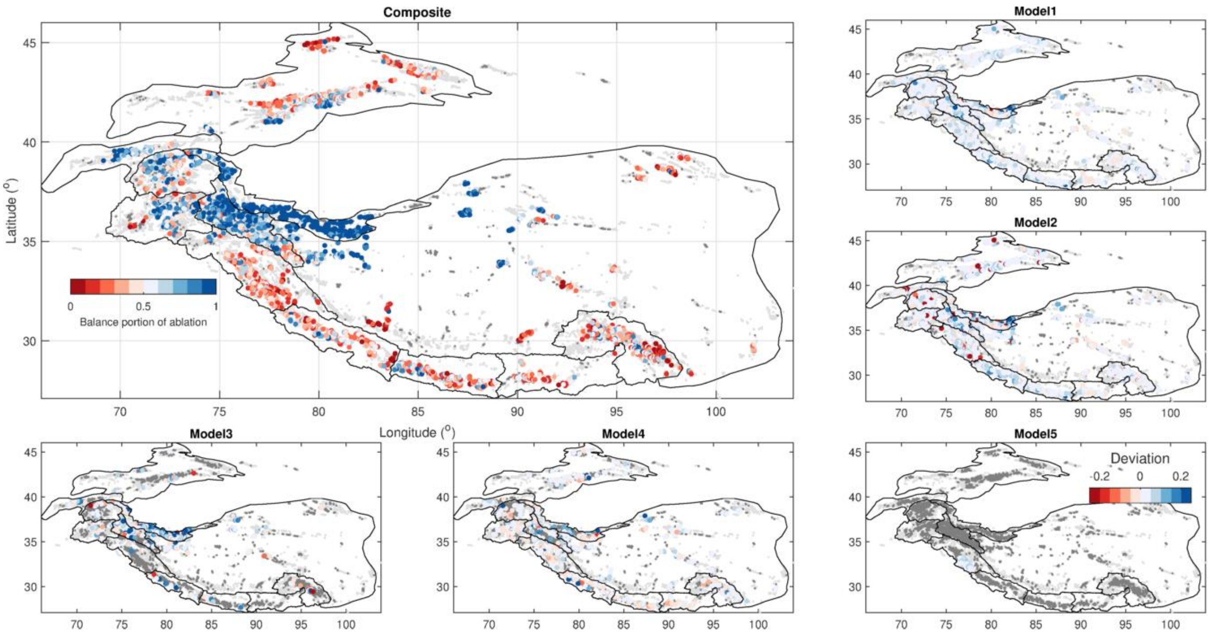


Supplementary Figure 95. Balance ablation estimates based on the consensus ice thickness (upper left, reproduced from Figure 4), along with deviations for the five ice thickness models contributing to the consensus. Note that Models 3-5 do not cover the entire region.

# SUPPLEMENTARY REFERENCES


1. Azam, M. F. *et al.* Review of the status and mass changes of Himalayan-Karakoram glaciers. *J. Glaciol.* **64**, 61–74 (2018).

2. Bolch, T. *et al.* Status and Change of the Cryosphere in the Extended Hindu Kush Himalaya Region. in *The Hindu Kush Himalaya Assessment* (eds. Wester, P., Mishra, A., Mukherji, A. & Shrestha, A. B.) 209–255 (Springer International Publishing, 2019). doi:10.1007/978-3-319-92288-1

3. Bolch, T. *et al.* The State and Fate of Himalayan Glaciers. *Science* **306**, 310–314 (2012).

4. WGMS. Fluctuations of Glaciers Database. (2019). doi:10.5904/wgms-fog-2019-12

5. Gardner, A. S. *et al.* Increased West Antarctic and unchanged East Antarctic ice discharge over the last 7 years. *Cryosphere* **12**, 521–547 (2018).

6. Dehecq, A. *et al.* Twenty-first century glacier slowdown driven by mass loss in High Mountain Asia. *Nat. Geosci.* **12**, 22–27 (2019).

7. Pfeffer, W. T. *et al.* The Randolph Glacier Inventory: a globally complete inventory of glaciers. *J. Glaciol.* **60**, 537–552 (2014).

8. The RGI Consortium. *Randolph Glacier Inventory – A Dataset of Global Glacier Outlines: Version 6.0, GLIMS Technical Report*. (2017).

9. Farinotti, D. *et al.* A consensus estimate for the ice thickness distribution of all glaciers on Earth. *Nat. Geosci.* **12**, 1 (2019).

10. Bisset, R. R. *et al.* Reversed Surface - Mass - Balance Gradients on Himalayan Debris - Covered Glaciers Inferred from Remote Sensing. *Remote Sens.* **12**, (2020).

11. Rabatel, A. *et al.* Can the snowline be used as an indicator of the equilibrium line and mass balance for glaciers in the outer tropics? *J. Glaciol.* **58**, 1027–1036 (2012).

12. Scherler, D., Bookhagen, B. & Strecker, M. R. Hillslope-glacier coupling: The interplay of topography and glacial dynamics in High Asia. *J. Geophys. Res. Earth Surf.* **116**, 1–21 (2011).

13. Yao, T. *et al.* Different glacier status with atmospheric circulations in Tibetan Plateau and surroundings. *Nat. Clim. Chang.* **2**, 663–667 (2012).

14. Gardelle, J., Berthier, E., Arnaud, Y. & Kääb, a. Region-wide glacier mass balances over the Pamir-Karakoram-Himalaya during 1999–2011. *Cryosph.* **7**, 1263–1286 (2013).

15. Loibl, D. M. & Lehmkuhl, F. Glaciers and equilibrium line altitudes of the eastern Nyainqêntanglha Range, SE Tibet. *J. Maps* **11**, 575–588 (2015).

16. Racoviteanu, A. E., Rittger, K. & Armstrong, R. An Automated Approach for Estimating Snowline Altitudes in the Karakoram and Eastern Himalaya From Remote Sensing. *Front. Earth Sci.* **7**, (2019).

17. Spiess, M., Schneider, C. & Maussion, F. MODIS-derived interannual variability of the equilibrium-line altitude across the Tibetan Plateau. *Ann. Glaciol.* **57**, 140–154 (2016).

18. Herreid, S. & Pellicciotti, F. The state of rock debris covering Earth's glaciers. *Nat. Geosci.* **13**, 621–627 (2020).

19. Hubbard, A. *et al.* Glacier mass-balance determination by remote sensing and high-resolution modelling. *J. Glaciol.* **46**, 491–498 (2000).

20. Nuimura, T. *et al.* Temporal Changes in Elevation of the Debris-Covered Ablation Area of Khumbu Glacier in the Nepal Himalaya since 1978. *Arctic, Antarct. Alp. Res.* **43**, 246–255 (2011).

21. Nuimura, T., Fujita, K. & Sakai, A. Downwasting of the debris-covered area of Lirung Glacier in Langtang Valley, Nepal Himalaya, from 1974 to 2010. *Quat. Int.* 1–9 (2017). doi:10.1016/j.quaint.2017.06.066

22. Berthier, E. & Vincent, C. Relative contribution of surface mass-balance and ice-flux changes to the accelerated thinning of Mer de Glace, French Alps, over 1979-2008. *J. Glaciol.* **58**, 501–512 (2012).

23. Gabbud, C., Micheletti, N. & Lane, S. N. Response of a temperate alpine valley glacier to climate change at the decadal scale. *Geogr. Ann. Ser. A Phys. Geogr.* **98**, 81–95 (2016).

24. Brun, F. *et al.* Ice cliff contribution to the tongue-wide ablation of Changri Nup Glacier, Nepal, central Himalaya. *Cryosph.* **12**, 3439–3457 (2018).

25. Miles, E. S. *et al.* Surface Pond Energy Absorption Across Four Himalayan Glaciers Accounts for 1/8 of Total Catchment Ice Loss. *Geophys. Res. Lett.* **45**, 10464–10473 (2018).

26. Rounce, D. R., King, O., McCarthy, M., Shean, D. E. & Salerno, F. Quantifying Debris Thickness of Debris-Covered Glaciers in the Everest Region of Nepal Through Inversion of a Subdebris Melt Model. *J. Geophys. Res. Earth Surf.* **123**, 1094–1115 (2018).

27. Gao, H. *et al.* Post-20th century near-steady state of Batura Glacier: observational evidence of Karakoram Anomaly. *Sci. Rep.* **10**, 1–10 (2020).

28. Huss, M. & Farinotti, D. Distributed ice thickness and volume of all glaciers around the globe. *J. Geophys. Res.* **117**, 1–10 (2012).

29. Jakob Fürst, J. *et al.* Application of a two-step approach for mapping ice thickness to various glacier types on Svalbard. *Cryosphere* **11**, 2003–2032 (2017).

30. Frey, H. *et al.* Estimating the volume of glaciers in the Himalayan & Karakoram region using different methods. *Cryosph.* **8**, 2313–2333 (2014).

31. Kenzhebaev, R. *et al.* Mass balance observations and reconstruction for Batysh Sook Glacier, Tien Shan, from 2004 to 2016. *Cold Reg. Sci. Technol.* **135**, 76–89 (2017).

32. Hoelzle, M. *et al.* Re-establishing a monitoring programme for glaciers in Kyrgyzstan and Uzbekistan, Central Asia. *Geosci. Instrumentation, Methods Data Syst.* **6**, 397–418 (2017).

33. Liang, L., Cuo, L. & Liu, Q. The energy and mass balance of a continental glacier: Dongkemadi Glacier in central Tibetan Plateau. *Sci. Rep.* **8**, 1–8 (2018).

34. Wang, R., Liu, S., Shangguan, D., Radić, V. & Zhang, Y. Spatial heterogeneity in glacier mass-balance sensitivity across High Mountain Asia. *Water (Switzerland)* **11**, 1–21 (2019).

35. Yu, W. *et al.* Different region climate regimes and topography affect the changes in area and mass balance of glaciers on the north and south slopes of the same glacierized massif (the West Nyainqentanglha Range, Tibetan Plateau). *J. Hydrol.* **495**, 64–73 (2013).

36. Wu, X., He, J., Jiang, X. & Wang, N. Analysis of surface energy and mass balance in the accumulation zone of Qiyi Glacier, Tibetan Plateau in an ablation season. *Environ. Earth Sci.* **75**, 1–13 (2016).

37. Zhu, M. *et al.* Reconstruction of the mass balance of Muztag Ata No. 15 glacier, eastern Pamir, and its climatic drivers. *J. Glaciol.* **64**, 259–274 (2018).

38. Zhu, M. *et al.* Differences in mass balance behavior for three glaciers from different climatic regions on the Tibetan Plateau. *Clim. Dyn.* **50**, 3457–3484 (2018).

39. Koul, M. N. & Ganjoo, R. K. Impact of inter- and intra-annual variation in weather parameters on mass balance and equilibrium line altitude of Naradu Glacier (Himachal Pradesh), NW Himalaya, India. *Clim. Change* **99**, 119–139 (2010).

40. Soheb, M. *et al.* Mass-balance observation, reconstruction and sensitivity of Stok glacier, Ladakh region, India , between 1978 and 2019. *J. Glaciol.* 1–16 (2020).

41. Laha, S. *et al.* Evaluating the contribution of avalanching to the mass balance of Himalayan glaciers. *Ann. Glaciol.* **58**, 110–118 (2017).

42. Wagnon, P. *et al.* Four years of mass balance on Chhota Shigri Glacier, Himachal Pradesh, India, a new benchmark glacier in the western Himalaya. *J. Glaciol.* **53**, 603–611 (2007).

43. Azam, M. F. *et al.* Meteorological conditions, seasonal and annual mass balances of Chhota Shigri Glacier, western Himalaya, India. *Ann. Glaciol.* **57**, 328–338 (2016).

44. Sharma, P. *et al.* Role of debris cover to control specific ablation of adjoining batal and sutri dhaka glaciers in chandra basin (Himachal Pradesh) during peak ablation season. *J. Earth Syst. Sci.* **125**, 459–473 (2016).

45. Pratap, B. *et al.* Reconciling High Glacier Surface Melting in Summer with Air Temperature in the Semi-Arid Zone of Western Himalaya. *Water* **11**, 1–18 (2019).

46. Tshering, P. & Fujita, K. First in situ record of decadal glacier mass balance (2003–2014) from the Bhutan Himalaya. *Ann. Glaciol.* **57**, 289–294 (2016).

47. Wagnon, P. *et al.* Seasonal and annual mass balances of Mera and Pokalde glaciers (Nepal Himalaya) since 2007. *Cryosphere* **7**, 1769–1786 (2013).

48. Sherpa, S. F. *et al.* Contrasted surface mass balances of debris-free glaciers observed between the southern and the inner parts of the Everest region (2007–15). *J. Glaciol.* 1–15 (2017). doi:10.1017/jog.2017.30

49. Sunako, S., Fujita, K., Sakai, A. & Kayastha, R. B. Mass balance of Trambau Glacier, Rolwaling region, Nepal Himalaya: In-situ observations, long-term reconstruction and mass-balance sensitivity. *J. Glaciol.* 1–12 (2019). doi:10.1017/jog.2019.37

50. Fujita, K., Kadota, T., Rana, B., Kayastha, R. & Ageta, Y. Shrinkage of Glacier AX010 in Shorong region, Nepal Himalayas in the 1990s. *Bull. Glaciol. Res.* **18**, 51–54 (2001).

51. Fujita, K. & Nuimura, T. Spatially heterogeneous wastage of Himalayan glaciers. *Proc. Natl. Acad. Sci. U. S. A.* **108**, 14011–14014 (2011).

52. Wang, P. *et al.* Primary investigation of statistical correlation between changes in ice volume and area of glaciers. *Sci. Cold Arid Reg.* **11**, 41–49 (2019).

53. Vincent, C. *et al.* Reduced melt on debris-covered glaciers: investigations from Changri Nup Glacier, Nepal. *Cryosph.* **10**, 1845–1858 (2016).

54. Baral, P. *et al.* Preliminary results of mass-balance observations of Yala Glacier and analysis of temperature and precipitation gradients in Langtang Valley, Nepal. *Ann. Glaciol.* **55**, 9–14 (2014).

55. Acharya, A. & Kayastha, R. B. Mass and energy balance estimation of Yala Glacier (2011-2017), Langtang Valley, Nepal. *Water (Switzerland)* **11**, (2018).

56. Gurung, S. *et al.* Study of annual mass balance (2011 – 2013) of Rikha Samba Glacier, Hidden Valley, Mustang, Nepal. *Sci. Cold Arid Reg.* **8**, 311–318 (2016).

57. Singh, S. S., Banerjee, A., Nainwal, H. C. & Shankar, R. Estimation of the total sub-debris ablation from point-scale ablation data on a debris-covered glacier. *J. Glaciol.* 1–11 (2019). doi:10.31223/OSF.IO/HU7NA

58. Dobhal, D. P., Mehta, M. & Srivastava, D. Influence of debris cover on terminus retreat and mass changes of Chorabari Glacier, Garhwal region, central Himalaya, India. *J. Glaciol.* **59**, 961–971 (2013).

59. Pratap, B., Dobhal, D. P., Mehta, M. & Bhambri, R. Influence of debris cover and altitude on glacier surface melting: a case study on Dokriani Glacier, central Himalaya, India. *Ann. Glaciol.* **56**, 9–16 (2015).

60. Yu, J., He, Y., Li, S., Wang, S. & Niu, H. Mass balance of a typical monsoonal temperate glacier in Hengduan Mountains Region. *Dili Xuebao/Acta Geogr. Sin.* **70**, 1415–1422 (2015).

61. Zhao, H. *et al.* Dramatic mass loss in extreme high-elevation areas of a western Himalayan glacier: observations and modeling. *Sci. Rep.* **6**, 30706 (2016).

62. Wei, Y., Tandong, Y., Baiqing, X. & Hang, Z. Influence of supraglacial debris on summer ablation and mass balance in the 24k glacier, southeast tibetan plateau. *Geogr. Ann. Ser. A Phys. Geogr.* **92**, 353–360 (2010).

63. Brun, F., Berthier, E., Wagnon, P., Kääb, A. & Treichler, D. A spatially resolved estimate of High Mountain Asia glacier mass balances from 2000 to 2016. *Nat. Geosci.* 668–673 (2017). doi:10.1038/ngeo2999

64. Pritchard, H. D. Asia's shrinking glaciers protect large populations from drought stress. *Nature* **569**, 649–654 (2019).

65. Shean, D. E. *et al.* A Systematic, Regional Assessment of High Mountain Asia Glacier Mass Balance. *Front. Earth Sci.* **7**, (2020).

66. Rounce, D. R., Hock, R., Shean, D. & Khurana, T. Quantifying glacier mass change in High Mountain Asia through 2100 using the open-source Python Galcier Evolution Model (PyGEM). *Front. Earth Sci.* **7**, 1–20 (2020).

67. Kaser, G., Großhauser, M., Marzeion, B. & Barry, R. G. Contribution potential of glaciers to water availability in different climate regimes. *Proc. Natl. Acad. Sci. U. S. A.* **107**, 21300–21305 (2010).

68. Huss, M. Density assumptions for converting geodetic glacier volume change to mass change. *Cryosph.* **7**, 877–887 (2013).

69. Immerzeel, W. W. *et al.* Importance and vulnerability of the world's water towers. *Nature* **577**, 364–369 (2020).

70. Scherler, D., Wulf, H. & Gorelick, N. Global Assessment of Supraglacial Debris-Cover Extents. *Geophys. Res. Lett.* **45**, 11,798-11,805 (2018).

71. Chen, F. *et al.* Annual 30-meter Dataset for Glacial Lakes in High Mountain Asia from 2008 to 2017. *Earth Syst. Sci. Data Discuss.* **30**, 1–29 (2020).

72. Zhang, Q. *et al.* Glacier change in the Gangdise Mountains, southern Tibet, since the Little Ice Age. *Geomorphology* **306**, 51–63 (2018).
